# The association between populist-aligned views and engagement with public health interventions: a systematic review of longitudinal studies

**DOI:** 10.1186/s12889-026-28020-w

**Published:** 2026-06-16

**Authors:** Kaitlin Conway-Moore, Jack M. Birch, Alison R. McKinlay, Fiona Graham, Emily J. Oliver, Clare Bambra, Michael P. Kelly, Chris Bonell

**Affiliations:** 1 NIHR Policy Research Unit Behavioural and Social Sciences, Department of Public Health, Environments and Society, London School of Hygiene and Tropical Medicine, London, WC1H 9SH UK; 2 NIHR Policy Research Unit Behavioural and Social Sciences, Population Health Sciences Institute, Faculty of Medical Sciences, Newcastle University, Newcastle Upon Tyne, UK; 3 NIHR Policy Research Unit Behavioural and Social Sciences, Centre for Behaviour Change, Department of Clinical, Educational and Health Psychology, University College London, London, UK; 4 Department of Public Health and Primary Care, Cambridge Public Health, University of Cambridge, Cambridge, UK

**Keywords:** Populism, Populist Attitudes, Public Health Interventions, Systematic Review, Quantitative Evidence, Longitudinal Evidence

## Abstract

**Background:**

Population-level engagement with public health interventions is needed to achieve impact. Recently, however, there has been evidence of increasing resistance to government-led public health interventions in areas such as vaccination, climate change mitigation, sexual and reproductive healthcare, and non-pharmaceutical-based infection control measures. The rise in populist attitudes that has taken place in many countries over the last two decades may be one potential explanation for this phenomenon, given the importance placed on individual freedom and national sovereignty, and a distrust of scientific, government and other elites within much populist discourse.

**Methods:**

To understand how populist-aligned views might subsequently influence the receipt of public health interventions, we systematically reviewed quantitative, longitudinal evidence across thirteen bibliographic databases and relevant websites, published between 2008 and 2024. All studies were set in Organisation for Economic Co-operation and Development (OECD) countries and results were synthesised narratively.

**Results:**

Across 16 included studies, much of the evidence focused on COVID-19. We found evidence that prior populist-aligned attitudes have a negative impact on subsequent receipt of public health interventions, such as the COVID-19 vaccine and non-pharmaceutical-based prevention measures against COVID-19 (i.e., engaging in masking and social distancing) through both existing and increasing distrust in elite institutions and actors over time. We also found preliminary evidence about the potentially negative role of populist-aligned attitudes on the receipt of other vaccinations before and during the COVID-19 pandemic.

**Conclusions:**

From a policy perspective, the findings from this review suggest the need for proactive efforts by political, scientific and medical establishments to build and maintain trust, thereby strengthening the reputation of both the public health leaders and institutions that engage with those more likely to be resistant to public health interventions to increase overall acceptance and uptake.

**Trial registration:**

PROSPERO registration number CRD4202451312.

**Supplementary Information:**

The online version contains supplementary material available at 10.1186/s12889-026-28020-w.

## Background

In 2019, the World Health Organization (WHO) declared vaccine hesitancy among the top ten threats to health globally [1]. With this announcement, attention was drawn to a growing body of evidence on how the resistance to government-led public health interventions occurring in countries around the world could affect their impact [2]. Along with vaccination, other types of public health intervention that have elicited a negative public reaction include efforts to mitigate the health effects of climate change, expanded access to sexual and reproductive healthcare, and the implementation of non-pharmaceutical infection control measures [3–7].

Among the theorised potential drivers of backlash against these government-led public health interventions is the rise in populist politics and attitudes that has taken place in many countries in recent years [3]. A highly contested political term that is more often ascribed to rather than claimed by actors, populism can be understood as both a strategy for politicians of varying ideological positions to gain power and influence among their constituents and as a discourse for framing such arguments [8, 9]. Populism can also be understood as a more diffuse constellation of populist-aligned views held by citizens influenced by populist political discourses, where each citizen will not necessarily hold all the beliefs associated with populism or even explicitly label their beliefs as populist [9].

Key aspects of populist discourse that might inform and/or be representative of such individually held, populist-aligned views include: feelings of dissatisfaction with and/or distrust of mainstream government and institutions; the idea of a virtuous or pure ‘people’ in moral struggle with a corrupt or out-of-touch ‘elite’ comprising government, business, science, professionals and the media; and the view that such elites are depriving the people of their individual freedom and/or collective sovereignty, or are pushing for unwelcome, progressive social change [8, 10–12]. While who constitutes ‘the people’ and who ‘the elite’ varies over time and across the political spectrum, what matters in populist discourse is the clear distinction of ‘us’ versus ‘them.’ In left-wing populist discourse, this distinction is largely binary, with those economically in the middle and bottom of society viewed as let down by those at the top. In right-wing populist discourse, the distinction is more often triadic, with ‘the people’ seen in opposition to one or more ‘elite’ groups, as well as various ‘othered’ or ‘out groups,’ such as women, migrants, and racial, ethnic, sexual, gender, linguistic or religious minorities [13, 14].

In this paper, we focus on an individual-level, broad conceptualisation of populism, i.e., views which align with key aspects of populist discourse and may significantly influence behaviour while not necessarily forming part of an explicit, coherent or conscious populist ideology. Research on science communication suggests that people’s beliefs about scientific issues, including health interventions, can be influenced by their cultural values, social identities and group affiliations [15]. As such, populist-aligned views could be an important component of how citizens’ views affect engagement with public health. With a growing body of research in this area now emerging, we sought to examine how people’s engagement with public health interventions might be affected by prior views which align with populist discourse.

Specifically, we report a synthesis of quantitative, longitudinal evidence to address the following research question: what are the patterns of association between populist-aligned views and subsequent attitudes towards and/or engagement with public health interventions among people living in Organisation for Economic Co-operation and Development (OECD) countries? We limited our synthesis to this subset of countries with economic similarities based on the fact that populist politics are widely regarded as having different causes and manifestations across different settings, for example due to different experiences of globalisation and economic conditions since the global financial crisis of 2008 [9, 16]. This synthesis was conducted as part of a larger systematic review of both qualitative and quantitative evidence on the ways populist-aligned views are linked to attitudes towards or engagement with public health interventions [17, 18] In the present paper, we focus on synthesising longitudinal quantitative evidence given the ability of this type of study design, and specifically its within-person measurements over time, to allow for inferences to be made regarding the temporality and directionality of associations between populist-aligned views and how public health interventions are viewed or received [19]. To our knowledge, this is both the first synthesis of longitudinal evidence on this topic, and the first that examines the potential public health implications of a broad array of individual-level populist-aligned views. As such, this work allows for novel contributions to be made to our understanding of this important area for public health, while also building on our prior syntheses of qualitative and cross-sectional quantitative evidence, which have been reported separately [17, 18].

## Methods

This systematic review is reported using the PRISMA 2020 Checklist (Appendix 1) [20]. The review protocol was prospectively registered with PROSPERO (registration number CRD42024513124) and published in 2024 [21].

### Inclusion and exclusion criteria

The findings from quantitative, longitudinal studies to be presented are part of a wider systematic review on the ways that populist-aligned attitudes are associated or linked with the receipt of public health interventions. As such, we report here inclusion criteria for the overall review as well as specifically for quantitative, longitudinal evidence. As mentioned above, included studies were limited to those involving adult participants living in member countries of the Organisation for Economic Co-operation and Development (OECD). Given the focus on populist-aligned views as our exposure measure of interest, we also limited our review to literature published since 2008, the year of the global financial crisis, from which most of the contemporary literature on populism dates [22].

To be included in this review, studies first needed to focus on population views commonly described in the research literature as rooted in populism (even if not explicitly defined as such by study authors) in terms of being hostile towards or distrustful of: 1) elites (e.g., government, business, medical and other health professionals, mainstream media, science, and the wealthy); 2) out-groups (e.g., women, migrants, minoritised ethnic, racial or religious groups or gender/sexual minorities); 3) checks on popular sovereignty (e.g., legal rights, personal freedoms, and other government-imposed regulations); or 4) social change (including moves towards greater social pluralism for example via promotion of diversity, equality and inclusion (DEI), state intervention or market regulation). This decision was made, firstly, because our conceptualisation of populism in this study was views aligning with populist belief that can be influential on behaviour even when not held as part of an explicit or coherent populist worldview and, secondly, because populism is a highly contested socio-political construct, use of which is often associated with a critical perspective on those holding such views [9]. Therefore, to provide a consistent basis for inclusion and avoid biasing inclusion towards literature taking a particular perspective on the topic, we based inclusion on specific attitudes rather than on how authors conceptualised these attitudes.

Second, included studies had to report on receipt of at least one existing (i.e., non-hypothetical) public health intervention, such as: vaccination; disease screening; non-pharmaceutical-based infection control; sexual/reproductive health care; increased access to health care; climate change mitigation; road safety; anti-pollution measures; water fluoridation; gun control; mental health care; promotion of healthy diet and exercise; and interventions related to gambling, tobacco, alcohol or drug use, among others.

Third, included studies needed to report how populist-aligned attitudes were associated with attitudes towards, adherence to and/or uptake of these public health interventions.

### Search strategy and study selection

Searches for eligible studies were executed in thirteen bibliographic databases relevant to medical, psychological, economic and social scientific research: CINAHL; Dissertation Abstracts; Econlit; EMBASE; Global Health; Global Index Medicus; International Bibliography of the Social Sciences; Ovid MEDLINE; PsycINFO; Scopus; Social Policy and Practice; Sociological Research Online; and Web of Science (including Science Citation Index Expanded, Social Sciences Citation Index, Arts & Humanities Citation Index, and Emerging Sources Citation Index). Our search was not limited by language or publication type. Given the nature of our research question, these searches mainly included the use of free-text terms rather than controlled vocabularies such as medical subject headings (MeSH). [22].

Three concepts were taken from our inclusion criteria to develop a search string: populist attitude AND public health intervention AND intervention receipt/views. For each of these concepts, relevant free-text and, where applicable, controlled-vocabulary search terms were linked by’OR’. Final search terms were reviewed by a librarian at the London School of Hygiene and Tropical Medicine prior to execution. Appendix 2 contains the search strategy used in each of our included databases.

In addition to database searches, searches were run on the following websites, based on their relevance to our topic: Centers for Disease Control and Prevention; Community Research and Development Information Service; Drug and Alcohol Findings Effectiveness Bank; European Centre for Disease Prevention and Control; Google; Google Scholar; Intergovernmental Panel on Climate Change; International Planned Parenthood Federation; Marie Stopes International; The Campbell Library; Open Library; United Nations Environment Programme; and the World Health Organization.

Reference lists of all studies included in the wider systematic review were hand-searched for additional studies that met our inclusion criteria. Finally, we contacted subject experts for studies meeting our inclusion criteria that had not already been retrieved by our searches.

Results obtained from our searches were downloaded into EPPI-Reviewer 6, at which point duplicates were removed [23]. Pilot screening of titles and abstracts began among pairs of reviewers (comprising KCM together with either FG or CBo) who screened successive batches of 50 records, meeting to discuss any disagreements and consulting a third reviewer where necessary. Once a batch-level agreement of 90% was achieved, remaining titles and abstracts were divided among the group and screened for potential inclusion by one reviewer. The process for screening full texts was to use a similar approach. However, as a 90% batch agreement was never achieved, all full texts were screened by pairs of reviewers (comprising KCM together with either AMK or CBo), who met regularly to discuss disagreements, consulting a third reviewer where necessary.

### Data extraction and quality assessment

Details of all included studies were extracted by one reviewer (KCM) using Microsoft Excel, with cross-checking by a second reviewer (JB) [24].

Data extraction from longitudinal studies was carried out by one reviewer (KCM) using Microsoft Excel, with cross-checking by a second reviewer (JB) [24]. This extraction followed the review protocol by reporting: basic study details (first author, publication date, study location, and duration); study methods (data collection, sampling and sample size, participant characteristics, populist-aligned attitudes examined, intervention receipt descriptors, timing of outcome measurements, reliability of measures, control for confounding, attrition, and analytical approach); and results (metric of association, estimate of association and p-value/confidence interval).

The Cambridge Quality Checklists were used to assess the methodological quality of each included longitudinal study, with the three checklists considering: correlates (i.e., examining associations between variables in a given population); risk factors (i.e., variables that are associated with a particular outcome); and causal risk factors (i.e., variables that can change and, when changed, cause a change in risk for the given outcome) [25, 26]. Specifically: the correlate checklist includes questions related to sampling, sample size, response rate, measure of correlate and measure of outcome (for a total correlate score out of 5); the risk-factor checklist assesses the use of cross-sectional, retrospective or prospective data (for a total risk factor score out of 3); and the causal-risk-factor checklist assesses variation in the risk factor, risk factor balancing and analysis of change (for a total causal risk factor score of 7) [25]. Quality assessments were carried out independently by two reviewers (KCM and JB) who met to compare their assessments and discuss any disagreements before reaching an agreed appraisal score for study quality.

The strength of the evidence presented in our review was assessed using the Grading of Recommendations, Assessment, Development and Evaluations (GRADE) approach, which is a method developed for assessing pooled effect estimates or narrative synthesis [27, 28]. As per the GRADE guidelines, when assessing the included observational (i.e., non-randomised) studies, our assumed baseline level of certainty was “low” (as opposed to “high” for randomised studies). From this starting point, the certainty of the evidence presented was further lowered if there was evidence of: 1) risk of bias in study design or execution; 2) inconsistency in the results across included studies; 3) indirectness in the applicability or generalisability of results in relation to the research question; 4) imprecision in measurement (increasing the risk of random error); or 5) publication bias. Conversely, the certainty of evidence was raised if there was evidence of: 1) a large observed effect; 2) a dose–response gradient; or 3) opposite residual confounding [29]. Given the degree of heterogeneity across our included studies, the outcomes assessed via the GRADE framework were summarised into three categories (i.e., attitudes towards or uptake of the COVID-19 vaccine; COVID-19 prevention measures; and other vaccinations), rather than focusing on the many different iterations of these outcomes. Initial GRADE assessments were made by one reviewer (KCM), with cross-checking for accuracy by a second reviewer (JB).

### Data analysis and synthesis

Given the large volume of included studies, including many poor-quality, cross-sectional studies focused on COVID-19, we made the decision to deviate from the protocol by conducting a basic mapping of all included studies (i.e., describing basic study details, methods, sample and outcome measures) and then synthesising only the findings from 1) all qualitative studies, 2) all longitudinal, quantitative studies and 3) cross-sectional studies focused on public health interventions other than those related to COVID-19, the second of which are reported in this paper. This enabled the synthesis reported in this paper to focus on those quantitative studies that can best examine the temporality of association between populist-aligned views and receipt of public health interventions.

Due to major heterogeneity in terms of country context, pandemic phase context (for studies focused on COVID-19), population sub-group (e.g., age, ethnicity), measures of populist-aligned views (e.g., distrust or trust with different institutions or groups), distinctive measures and public health interventions, statistical meta-analysis was not appropriate, nor were subgroup meta-analyses or meta-regressions because of the relatively small number of included studies. Instead, findings were synthesised narratively, ordered by public health topic (i.e., COVID-19 vaccination, non-pharmaceutical based COVID-19 preventative measures and other vaccinations) and type of populist-aligned view (i.e., trust in various groups/institutions). [15].

### Patient and public involvement

The review benefited from the involvement of a designated member of the NIHR Policy Research Unit Behavioural and Social Sciences’ Patient and Public Involvement and Engagement (PPIE) Strategy Group. This person was regularly updated on the progress of the review. He provided plain language copy edits for our written outputs relating to our methods and emerging findings. These were then incorporated into final versions of our outputs to ensure that our work would be accessible and relevant to a wide audience both within and outside the health policy research community.

## Results

### Overview of included studies

**Fig. 1:**
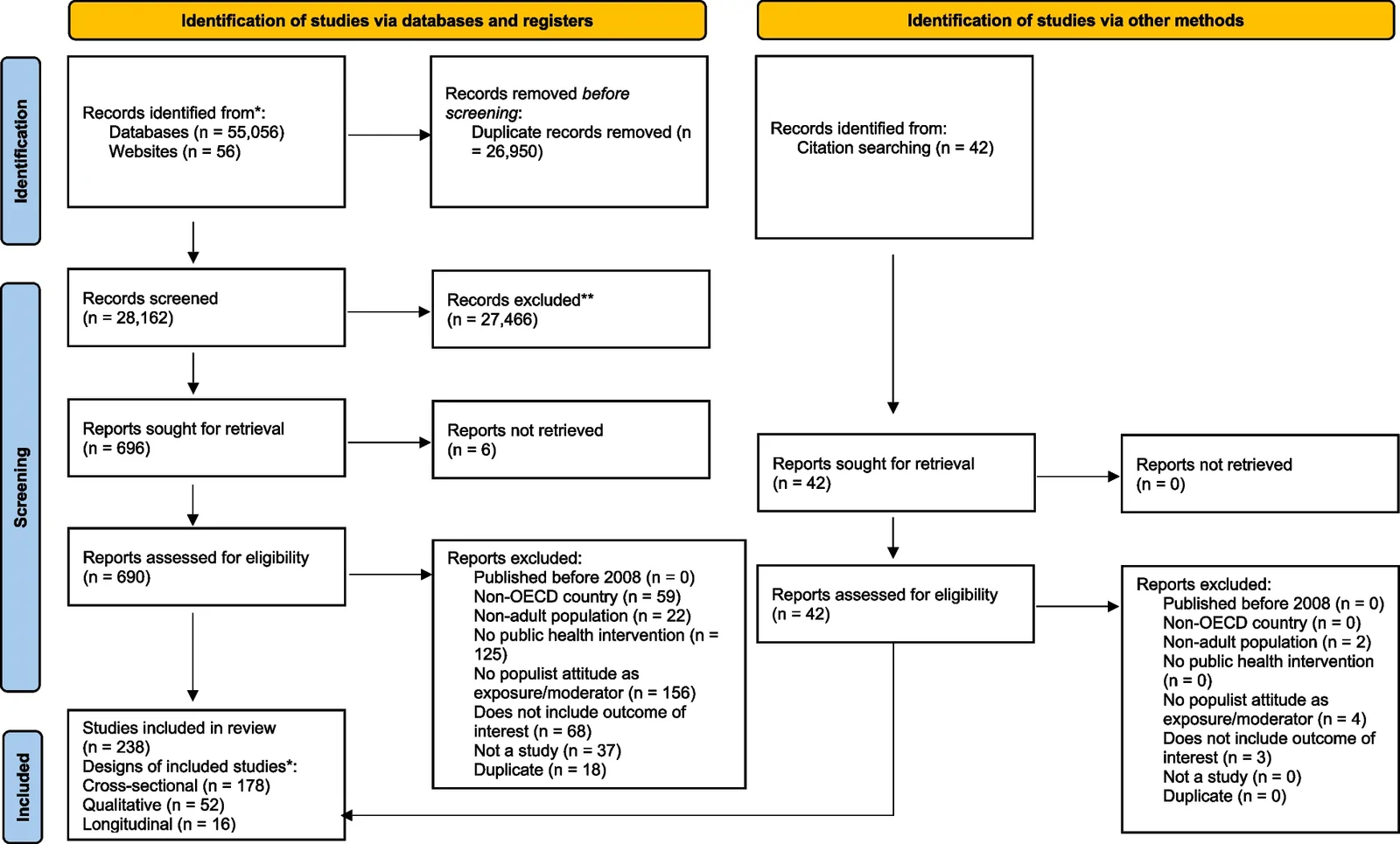
Preferred Reporting Items for Systematic Reviews and Meta-Analyses (PRISMA) flow diagram [20]. *The breakdown of included studies by study design totals to n = 246 rather than n = 238 as 8 studies included mixed methods

Figure 1 presents the results of our search strategy. We identified 55,056 references from databases and 56 references from websites, for a total of 55,112 references. De-duplication resulted in 28,162 unique references, and, after title and abstract screening, 690 references were marked for full-text screening. An additional six references were eligible for full-text screening but could not be retrieved. We identified 205 references for inclusion based on full-text screening, with an additional 33 references added based on hand-searching the reference lists of these included studies. Expert consultation identified no additional studies. Overall, this process resulted in 238 references, of which 16 are included in the present review, which is limited to only those studies that undertook longitudinal data analysis.

### Study characteristics

For an overview of the study characteristics of all 239 studies included in the wider systematic review, see Appendix 3. All included longitudinal studies were published between 2011 and 2024, with most from 2022 onwards (Table 1). Study settings ranged geographically and included the United States of America (USA) (*n* = 8), Poland (*n* = 4), United Kingdom (UK) (*n* = 3), France (*n* = 2), Germany (*n* = 2), Australia (*n* = 1), Austria (*n* = 1), Italy (*n* = 1), New Zealand (*n* = 1), Sweden (*n* = 1) and Switzerland (*n* = 1). All studies used survey-based data-collection methods with a minimum of two survey rounds, and sampling methods included convenience sampling (*n* = 11) and nationally or sub-nationally representative sampling (*n* = 4 and *n* = 1, respectively). Sample sizes varied from 73 participants to 80,906, while also including a range of participant characteristics, such as specific linguistic sub-populations (*n* = 1), adults experiencing homelessness (*n* = 1), essential workers (*n* = 1), parents of children under 16 (*n* = 1), veterans (*n* = 1), vaccinated adults (*n* = 1), registered voters (*n* = 1), and adult members of the general population of the study setting (*n* = 11). The timing from baseline to follow-up ranged from two to 34 months.

**Table 1:** Included longitudinal study characteristics and results (*n*= 16)

Study author (year)	Study Country (City or Region) and Duration	Sampling Method, Sample Size and Participant Characteristics	Timing of outcome measurement	Control of Confounding	Analysis & Metric of Association	Variable	Estimate of association	P value/confidence interval for estimate of association
						Total effect predictor of attitudes preventative measures: Populist attitudes	-0.2	p = 0.007
						Direct effect predictor of attitudes preventative measures: Populist attitudes	-0.04	p = 0.640
						Direct effect predictor of attitudes preventative measures: Trust in alternative news media	-0.09	p = 0.262
						Direct effect predictor of attitudes preventative measures: Trust in mainstream news media	-0.09	p = 0.420
						Direct effect predictor of attitudes preventative measures: Trust in political institutions	0.34	p = 0.009
						Direct effect predictor of attitudes preventative measures: Trust in scientific institutions	0.27	p = 0.001
						Indirect effect mediator of attitudes preventative measures: Trust in alternative news media	-0.022	Boot SE: 0.020, 95% CI: (–0.060, 0.017)
						Indirect effect mediator of attitudes preventative measures: Trust in mainstream news media	0.029	Boot SE: 0.036, 95% CI: (–0.039, 0.107)
						Indirect effect mediator: of attitudes preventative measures Trust in political institutions	-0.119	Boot SE: 0.049, 95% CI: (–0.222, –0.030)
Ehrke (2023) [39]	Germany and Poland; April-June 2021	Convenience sample; n= 216 adult participants; n =143 from Germany and n =73 from Poland	2 months after baseline	Controls for interpersonal trust, age, gender, education and income security	Multiple linear regression; Beta Coefficient	Indirect effect mediator of attitudes preventative measures: Trust in scientific institutions	-0.049	Boot SE: 0.020, 95% CI: (–0.091, –0.013)
						Total effect predictor of attitudes preventative measures: Populist attitudes	-0.2	p = 0.007
						Direct effect predictor of attitudes preventative measures: Populist attitudes	-0.04	p = 0.640
						Direct effect predictor of attitudes preventative measures: Trust in alternative news media	-0.09	p = 0.262
						Total effect predictor: Populist attitudes	-0.09	p = 0.215
						Direct effect predictor of adherence to preventative measures: Populist attitudes	0.04	0.643
						Direct effect predictor of adherence to preventative measures: Trust in alternative news Media	-0.07	0.397
						Direct effect predictor of adherence to preventative measures: Trust in mainstream news media	0.1	p = 0.43
						Direct effect predictor: Trust in political institutions	0.02	p = 0.013
						Direct effect predictor of adherence to preventative measures: Trust in scientific institutions	0.35	*p* <0.001
						Indirect effect mediator of adherence to preventative measures: Trust in mainstream news media	-0.034	Boot SE: 0.042, 95% CI: (–0.116, 0.052)
						Indirect effect mediator of adherence to preventative measures: Trust in political institutions	-0.008	Boot SE: 0.051, 95% CI: (–0.115, 0.088)
						Indirect effect mediator of adherence to preventative measures: Trust in scientific institutions	-0.063	Boot SE: 0.025, 95% CI: (–0.116, –0.018)
						Relationship between trust in scientists and vaccine intentions: All countries	0.127	SE: 0.01, *p* < 0.01
						Relationship between trust in scientists and vaccine intentions: EU countries (Austria, France, Germany, Italy and Sweden)	0.118	SE: 0.012, *p* < 0.01
						Relationship between trust in scientists and vaccine intentions: UK and US	0.175	SE: 0.030, *p* < 0.01
						Relationship between trust in scientists and vaccine intentions: Australia and New Zealand	0.121	SE: 0.030, *p* < 0.01
						Relationship between trust in scientists and anti-vaccine intentions: All countries	-0.156	SE: 0.016, *p* < 0.01
						Relationship between trust in scientists and hard anti-vaccine intentions: All countries	-0.132	SE: 0.013, *p* < 0.01
						Relationship between trust in scientists and soft anti-vaccine intentions: All countries	-0.024	SE: 0.012, *p* <0.10
						Relationship between trust in scientists and anti-vaccine intentions: EU countries (Austria, France, Germany, Italy and Sweden)	-0.152	SE: 0.019, *p* < 0.01
Galasso (2022) [36]	Australia, Austria, France, Germany, Italy, NewZealand, Sweden, the UK and the USA; December 2020-July 2021	Nationally representative samples; n=6,379 adult participants; n=343 in Australia, n=324 in Austria, n=850 in France, n= 1,481 in Germany, n= 710 in Italy, n= 639 in New Zealand, n= 693 in Sweden, n= 697 in the UK, and n= 642 in the US	7 months after baseline	Controls for age, education, occupation status and gender	Linear regression; Regression coefficient	Relationship between trust in scientists and anti-vaccine intentions: UK and US	-0.203	SE: 0.041, *p* < 0.01
						Relationship between trust in scientists and anti-vaccine intentions: Australia and New Zealand	-0.128	SE: 0.056, *p* < 0.05
						Relationship between trust in scientists and vaccine uptake: All countries	0.033	SE: 0.020, *p* < 0.10
						Relationship between trust in scientists and vaccine uptake: EU countries (Austria, France, Germany, Italy and Sweden)	0.008	SE: 0.025
						Relationship between trust in scientists and vaccine uptake: UK and US	0.089	SE: 0.023, *p* < 0.01
						Relationship between trust in scientists and vaccine uptake: Australia and New Zealand	0.076	SE: 0.035, *p* < 0.05
						Relationship between trust in scientists and vaccine uptake among those who had anti-vax intentions at Time 1: All countries	0.064	SE: 0.029, *p* < 0.05
						Relationship between trust in scientists and vaccine uptake among those who had anti-vax intentions at Time 1: EU countries (Austria, France, Germany, Italy and Sweden)	0.039	SE: 0.034
						Relationship between trust in scientists and vaccine uptake among those who had anti-vax intentions at Time 1: UK and US	0.119	SE: 0.101
						Relationship between trust in scientists and vaccine uptake among those who had anti-vax intentions at Time 1: Australia and New Zealand	0.113	SE: 0.042, *p* < 0.05
					Logistical hierarchical regression; Beta coefficient	Trust in medical organisations on vaccine uptake	0.76	SE: 0.21; *p*<0.001
						Trust in medical organisations on vaccine uptake	0.30	SE: 0.08; *p*<0.001
Gilles (2011) [42]	Switzerland; March 2009-December 2009	Convenience sample; n= 601 French-speaking Swiss adults	6 months after baseline	Controls for age, gender, residential area, education,income, and number of children	Hierarchical linear regression; Beta coefficient	Trust in medical organisations on perceived efficacy of washing hands	0.17	SE: 0.06; *p*<0.01
						Trust in medical organisations on perceived efficacy of wearing a mask	0.22	SE: 0.08; *p*<0.01
						Trust in COVID-19 information from official sources among those not vaccine hesitant	0.71, 95% CI: 0.56-0.83	p=0.06
						Trust in COVID-19 information from official sources among those vaccine hesitant	0.51, 95% CI: 0.36-0.66	
					Chi-square tests; Mean	Trust in COVID-19 information from official sources among total sample	0.62, 95% CI: 0.51-0.71	
Kuhn (2021) [30]	USA (Los Angeles); December 2020-February 2021	Convenience sample; n= 90 adults experiencing homelessness in Los Angeles	2 months after baseline	Controls for age, sex/gender and race/ethnicity		Trust in COVID-19 information from traditional media among those not vaccine hesitant	0.64, 95% CI: 0.49-0.77	p=0.09
						Trust in COVID-19 information from official sources among those vaccine hesitant	0.46, 95% CI: 0.32-0.62	
						Trust in COVID-19 information from official sources among total sample	0.56, 95% CI: 0.45-0.66	
						Trust in COVID-19 info from official sources on vaccine hesitancy	OR: 0.41	95% CI: (0.17, 0.99), p=0.05
					Logistic regression; Odds ratio	Trust in COVID-19 info from official sources on vaccine hesitancy	AOR: 0.37	95% CI:(0.12, 1.11), p=0 .08
						Trust in COVID-19 info from traditional media on vaccine hesitancy	OR: 0.50	95% CI: (0.21, 1.19), p=0.12
						Trust in COVID-19 info from traditional media on vaccine hesitancy	AOR: 0.52	95% CI:(0.19, 1.41), p=0 .20
						Trust in information source Wave 6 on COVID-19 vaccine uptake: CDC	4.05	95% CI: (2.52, 6.53)
						Trust in information source Wave 6 on COVID-19 vaccine uptake: White House	0.41	95% CI: (0.27, 0.62)
						Trust in information source Wave 6 on COVID-19 vaccine uptake: Johns Hopkins University	4.34	95% CI: (2.58, 7.30)
					Logistic regression; Odds ratio	Trust in information source Wave 6 on COVID-19 vaccine uptake: State Health Departments	3.53	95% CI: (2.29, 5.46)
						Trust in information source Wave 6 on COVID-19 vaccine uptake: Major News Outlets	3.43	95% CI: (2.28, 5.16)
						Trust in information source Wave 7 on COVID-19 vaccine uptake: CDC	4.32	95% CI: (2.47, 7.56)
						Trust in information source Wave 7 on COVID-19 vaccine uptake: White House	0.28	95% CI: (0.17, 0.47)
						Trust in information source Wave 7 on COVID-19 vaccine uptake: Johns Hopkins University	5.19	95% CI: (2.81, 9.57)
						Trust in information source Wave 7 on COVID-19 vaccine uptake: State Health Departments	3.11	95% CI: (1.85, 5.23)
Latkin (2023) [27]	USA; March 2020-November 2021	Convenience sample; n= 492 US adult participants at Time 1 and Time 2, n= 390 US adult participants at Time 3	15 months and 20 months after baseline	Controls for race/ethnicity, age, gender, household income, education		Trust in information source Wave 7 on COVID-19 vaccine uptake: Major News Outlets	5.76	95% CI: (3.27, 10.13)
						Trust in information source Wave 6 on COVID-19 vaccine uptake: CDC	1.76	95% CI: (0.91, 3.40)
						Trust in information source Wave 6 on COVID-19 vaccine uptake: White House	0.42	95% CI: (0.24, 0.74)
						Trust in information source Wave 6 on COVID-19 vaccine uptake: Johns Hopkins University	1.6	95% CI: (0.80, 3.20)
					Logistic regression; Adjusted odds ratio	Trust in information source Wave 6 on COVID-19 vaccine uptake: State Health Departments	2.41	95% CI: (1.37, 4.25)
						Trust in information source Wave 6 on COVID-19 vaccine uptake: Major News Outlets	2.03	95% CI: (1.26, 3.27)
						Trust in information source Wave 7 on COVID-19 vaccine uptake: CDC	2.69	95% CI: (1.12, 6.49)
						Trust in information source Wave 7 on COVID-19 vaccine uptake: White House	0.24	95% CI: (0.11, 0.51)
						Trust in information source Wave 7 on COVID-19 vaccine uptake: Johns Hopkins University	1.85	95% CI: (0.78, 4.35)
						Trust in information source Wave 7 on COVID-19 vaccine uptake: State Health Departments	1.52	95% CI: (0.71, 3.26)
						Trust in information source Wave 7 on COVID-19 vaccine uptake: Major News Outlets	3.54	95% CI: (1.82, 6.92)
					Ordinal logistic regression; Unadjusted odds ratio	Trust in government among those vaccinated during the study (compared to those not vaccinated)	4.4	95% CI: (3.87, 5.00)
					Ordinal logistic regression; Unadjusted odds ratio	Trust in government among those considered Reluctant to get the vaccine (compared to those who are Endorsers)	0.2	95% CI: (0.17, 0.23)
					Ordinal logistic regression; Adjusted odds ratio	Trust in government among those considered Reluctant to get the vaccine (compared to those who are Endorsers)	0.43	95% CI: (0.30, 0.61)
Lutrick (2022) [28]	USA; December 2020-May 2021	Convenience sample;n= 4,803 essential workers (health care personnel, frontline workers and first responders)	6 months from baseline	Controls for gender, age, race, ethnicity, education, householdincome, study site, occupation and occupational setting, participant health status, and COVID-19 vaccination status	Ordinal logistic regression; Unadjusted odds ratio	Trust in government among those considered Reachable to get the vaccine (compared to those who are Endorsers)	0.58	95% CI: ( 0.51, 0.67)
					First-differences analysis; Percent increase	Change in likelihood of vaccination status at Time 2 according to trust in government: Overall	9.4	SE: 0.00546, *p*<0.01
						Change in likelihood of vaccination status at Time 2 according to trust in government: Endorser	8.1	SE: 0.00748, *p*<0.01
						Change in likelihood of vaccination status at Time 2 according to trust in government: Reluctant & Reachable	6.3	SE: 0.00815, *p*<0.01
					Repeated measures ANOVA and multiple linear regression; ANOVA F statistic	ANOVA for Trust in Science between those unwilling, willing and undecided about vaccination at Time 1	F(2,872) = 29.182	*p* < 0.001, η2 = 0.063
						Trust in science among those unwilling to vaccinate at Time 1	M = 3.49, SD = 0.82	*p* < 0.001
						Trust in science among those willing to vaccinate at Time 1	M = 3.97, SD = 0.64	
					Repeated measures ANOVA and multiple linear regression; Pairwise comparisons (Bonferroni adjusted)	Trust in science among those undecided at Time 1	M = 3.61, SD = 0.68	*p* < 0.001
						Trust in science among those willing to vaccinate at Time 1	M = 3.97, SD = 0.64	
						Trust in science among those unwilling to vaccinate at Time 1	M = 3.49, SD = 0.82	*p* = 0.14
						Trust in science among those undecided at Time 1	M = 3.61, SD = 0.68	
					Repeated measures ANOVA and multiple linear regression; ANOVA F statistic	ANOVA for Trust in Science between those unvaccinated, vaccinated and undecided at Time 2	F(2,915) = 41.268	*p* < 0.001, η2 = 0.083
						Trust in science among the unvaccinated at Time 2	M = 3.453, SD = 0.88	*p* < 0.001
						Trust in science among the vaccinated at Time 2	M = 3.898, SD = 0.69	
						Trust in science among the vaccine undecided at Time 2	M = 3.574, SD = 0.68	*p* < 0.001
Maciuszek (2023) [38]	Poland; February-August 2021	Nationally representative sample; n= 918 Polish adult participants	6 months from baseline	Controls for sex, age and place of residence	Repeated measures ANOVA and multiple linear regression; Pairwise comparisons (Bonferroni adjusted)	Trust in science among the vaccinated at Time 2	M = 3.898, SD = 0.69	
						Trust in science among the unvaccinated at Time 2	M = 3.453, SD = 0.88	p = 0.454
						Trust in science among the vaccine undecided at Time 2	M = 3.574, SD = 0.68	
					Repeated measures ANOVA and multiple linear regression; ANOVA F statistic	ANOVA for Trust in Science (Time 2) between 7 groups (Consistently Pro-vaccine; Consistently Anti-vaccine; Consistently Undecided; Anti-vaccine turned Pro-vaccine; Anti-vaccine turned Undecided; Undecided turned Pro-vaccine; and Undecided turned Anti-vaccine) for differences in vaccine attitudes and vaccine willingness between Time 1 and Time 2	F(6,854) = 8.633	*p* < 0.001, η2 = 0.106
					Repeated measures ANOVA and multiple linear regression; Pairwise comparisons (Bonferroni adjusted)	Trust in science among those who were Consistently Pro-vaccine at Time 1 and 2	M = 4.03, SD = 0.67	Highest p = 0.005
						Trust in science among all other groups at Time 1 and Time 2	Consistently Anti-vaccine: M = 3.43, SD = 0.91;Anti-vaccine turned Undecided: M = 3.58, SD = 0.81; Anti-vaccine turned Pro-vaccine: M = 3.58, SD = 0.57; Undecided turned Anti-vaccine: M = 3.53, SD = 0.80; Consistently Undecided: M = 3.54, SD = 0.60; Undecided turned Pro-vaccine: M = 3.66, SD = 0.69	
						Trust in science at Time 1 for Undecided to Anti-vaccine Group	M = 3.70, SD = 0.76	t(29) = 2.430, p = 0.022
						Trust in science at Time 2 for Undecided to Anti-vaccine Group	M = 3.53, SD = 0.80	
					Repeated measures ANOVA and multiple linear regression; Paired-sample T test	Trust in science at Time 1 for Consistently Pro-vaccine Group	M = 3.98, SD = 0.65	t(378) = 1.990, p = 0.047
						Trust in science at Time 2 for Consistently Pro-vaccine Group	M = 4.03, SD = 0.67	
						Within-Subject Relationship between Governmental Conspiracy and Preventative Behaviours	0.003	SE: 0.013, p = 0.822
						Within-Subject Relationship between Preventative Behaviours and Governmental Conspiracy	0.118	SE: 0.103, p = 0.251
						4 Wave Between-Subject Correlation between Governmental Conspiracy and Preventative Behaviours	0.029	SE: 0.028, p = 0.305
Oleksy (44)	Poland; May-December 2020	Nationally representative sample; n= 1,130 Polish adult participants at Time 1, n= 971 at Time 2, n= 818 at Time 3, n= 688 at Time 4	8 months from baseline	Controls for age, sex and education level	Random-intercept cross-lagged panel models; Beta coefficient	Within-Subject Relationship between Threat of Authoritarianism and Preventative Behaviours	-0.074	SE: 0.02, *p* <0.001
						Within-Subject Relationship between Preventative Behaviours and Threat of Authoritarianism	-0.235	SE: 0.085, p = 0.006
						4 Wave Between-Subject Correlation between Threat of Authoritarianism and Preventative Behaviours	0.003	SE: 0.027, p = 0.922
						Direct effect of low confidence in the central UK government (ref high) on COVID-19 vaccine refusal	0.94	95% CI: (0.16, 5.45)
						Direct effect of low confidence in the UK health service (ref high) on COVID-19 vaccine refusal	7.51	95% CI: (2.05, 27.43)
Paul (2022) [32]	UK; July 2020-June 2021	Convenience sample;n= 633 UK adult participants from the COVID-19 Social Study	6-12 months from baseline	Controls for gender, education level, age, long-term physical health condition, having been infected with COVID-19	Structural equation modelling with logistic regression; Odds ratio	Indirect effect of racial/ethnic discrimination on COVID-19 vaccine refusal through lowconfidence in the central UK government to handle the pandemic	0.98	95% CI: (0.58, 1.67)
						Indirect effect of racial/ethnic discrimination on COVID-19 vaccine refusal through lowconfidence in the UK health service to handle the pandemic	2.46	*p* <0.05, 95% CI: (1.12, 5.39)
						Total effect of racial/ethnic discrimination on COVID-19 vaccine refusal (direct effect ofracial/ethnic discrimination plus indirect effects of low confidence in governmentand the health system)	3.91	*p* <0.05, 95% CI: (1.40, 10.92)
						Children aged 12+ Trust in government information about the vaccine on uptake: Strongly or mildly agree (Ref: Neutral)	7.2	95% CI: (4.28, 12.12)
						Children aged 12+: Trust in government information about the vaccine on uptake: Strongly or mildly disagree (Ref: Neutral)	0.51	95% CI: (0.30, 0.87)
					Multivariable logistic regression; Odds ratio	Children aged 5-11: Trust in government information about the vaccine on uptake: Strongly or mildly agree (Ref: Neutral)	8.43	95% CI: (5.75, 12.34)
						Children aged 5-11: Trust in government information about the vaccine on uptake: Strongly or mildly disagree (Ref: Neutral)	1.44	95% CI: (0.92, 2.23)
						Children aged <5: Trust in government information about the vaccine on uptake: Strongly or mildly agree (Ref: Neutral)	7.31	95% CI: (3.68, 14.53)
						Children aged <5: Trust in government information about the vaccine on uptake: Strongly or mildly disagree (Ref: Neutral)	3.27	95% CI: (1.37, 7.83)
						Children aged 12+ Trust in government information about the vaccine on uptake: Strongly or mildly disagree (Ref: Neutral)	0.34	95% CI: (0.18, 0.67)
					Multivariable logistic regression; Adjusted odds ratio	Children aged 5-11: Trust in government information about the vaccine on uptake: Strongly or mildly agree (Ref: Neutral)	7.43	95% CI: (4.65, 11.88)
						Children aged 5-11: Trust in government information about the vaccine on uptake: Strongly or mildly disagree (Ref: Neutral)	1.59	95% CI: (0.95, 2.65)
						Change in parental attitude towards trust in government information about vaccine between July 2021 and October 2022 among parents of vaccinated children: negative to positive	9.5%	<0.0001
						Change in parental attitude towards trust in government information about vaccine between July 2021 and October 2022 among parents of vaccinated children: positive to negative	25.2%	
						Change in parental attitude towards trust in government information about vaccine between July 2021 and October 2022 among parents of unvaccinated children: negative to positive	12.3%	<0.0006
Rivers (2024) [29]	USA (Arizona, Florida, Texas and Utah); July 2021-October 2022	Convenience sample; n= 1,121-2,562 US children aged 6 months to 17 years enrolled in the Pediatric Research Observing Trends and Exposures in COVID-19 Timelines (PROTECT) study (data collected from children's parents, sample size is outcome dependent)	15 months from baseline	Controls for recruitment city, child's age at enrolment, child's sex at birth, child's age/ethnicity, COVID-19 infection prior to enrolment, parents' health insurance status and annual household income	Multivariable logistic regression and McNemar's test for paired samples	Change in parental attitude towards trust in government information about vaccine between July 2021 and October 2022 among parents of unvaccinated children: positive to negative	23.2%	
						Change in parental attitude towards trust in government information about vaccine between July 2021 and October 2022 among parents unlikely to vaccinate at baseline: negative to positive	20.7%	0.211
						Change in parental attitude towards trust in government information about vaccine between July 2021 and October 2022 among parents unlikely to vaccinate at baseline: positive to negative	13.2%	
						Change in parental attitude towards trust in government information about vaccine between July 2021 and October 2022 among parents undecided about vaccination at baseline: negative to positive	8.6%	0.011
						Change in parental attitude towards trust in government information about vaccine between July 2021 and October 2022 among parents undecided about vaccination at baseline: positive to negative	19.8%	
						Change in parental attitude towards trust in government information about vaccine between July 2021 and October 2022 among parents likely to vaccinate at baseline: negative to positive	9.2%	<0.0001
						Change in parental attitude towards trust in government information about vaccine between July 2021 and October 2022 among parents likely to vaccinate at baseline: positive to negative	26.7%	
						Pathway 1: Relationship between Trust in Authorities and Attitude towards COVID-19 Vaccination of Child	0.31	99% CI: (0.23, 0.40)
						Pathway 1: Relationship between Conspiracy Mindset and Trust in Authorities	-0.74	99% CI: (-0.78, -0.70)
						Pathway 2: Relationship between Trust in Authorities and COVID-19 Risk	0.32	99% CI: (0.19, 0.45)
						Pathway 2: Relationship between COVID-19 Risk and Attitude towards COVID-19 Vaccination of Child	0.28	99% CI: (0.23, 0.33)
						Continued to trust scientists: Undecided	1.83	95% CI: (1.46, 2.3)
						Began to trust scientists: Vaccine Undecided	1.91	95% CI: (1.51, 2.43)
						Lost trust in scientists: Vaccine Undecided	0.84	95% CI: (0.64, 1.09)
						Continued to trust government: Vaccine Undecided	1.71	95% CI: (1.5, 1.95)
						Began to trust government: Vaccine Undecided	1.69	95% CI: (1.46, 1.94)
						Continued to trust scientists: Undecided	0.82	95% CI: (0.71, 0.94)
						Began to trust scientists: Vaccine Undecided	2.06	95% CI: (1.84, 2.31)
						Lost trust in scientists: Vaccine Undecided	1.93	95% CI: (1.7, 2.17)
						Continued to trust government: Vaccine Undecided	0.92	95% CI: (0.8, 1.07)
Spire (2023) [35]	France; November 2020-July 2021	Nationally representative sample; n= 80,906 French adult participants from the Epidemiology andLiving Conditions (EpiCoV) study	8 months from baseline	Controls for age, gender, race/ethnicity), social class (based on current/last occupation), standard of living (based on decileof household income per consumption unit) and education	Logistic regression; Odds ratio	Began to trust government: Vaccine Undecided	2.12	95% CI: (1.94, 2.33)
						Continued to trust scientists: Undecided	2.14	95% CI: (1.94, 2.36)
						Began to trust scientists: Vaccine Undecided	0.88	95% CI: (0.8, 0.97)
						Lost trust in scientists: Vaccine Undecided	0.46	95% CI: (0.39, 0.54)
						Continued to trust government: Vaccine Undecided	0.54	95% CI: (0.45, 0.65)
						Began to trust government: Vaccine Undecided	1.08	95% CI: (0.88, 1.32)
						Continued to trust scientists: Undecided	0.55	95% CI: (0.51, 0.60)
						Began to trust scientists: Vaccine Undecided	0.56	95% CI: (0.51, 0.61)
						Lost trust in scientists: Vaccine Undecided	1.14	95% CI: (1.04, 1.25)
						Trust in physicians on attitudes towards vaccination (in general) in 2018	0.228	SE: 0.061; *p*< 0.001
						Trust in physicians on attitudes towards vaccination (in general) in 2020	0.367	SE: 0.054; *p*< 0.001
						Trust in science on attitudes towards vaccination (in general) in 2018	0.3	SE: 0.053; *p*< 0.001
						Trust in science on attitudes towards vaccination (in general) in 2020	0.362	SE: 0.051; *p*< 0.001
Stasiuk (2021) [37]	Poland; February 2018-December 2020	Convenience sample; n=400 Polish adults	34 months from baseline	Controls for sex, age, place of residence	Multiple linear regression; Beta coefficient	Change in trust in physicians between 2018 and 2020 on attitudes towards vaccination (in general) in 2020	0.244	SE: 0.059; *p*< 0.001
						Change in trust in scientists between 2018 and 2020 on attitudes towards vaccination (in general) in 2020	0.288	SE: 0.058; *p*< 0.001
						Trust in physicians as a predictor of attitudes towards COVID-19 vaccination in 2020	0.23	SE: 0.042; *p*<0.05
						Trust in scientists as a predictor of attitudes towards COVID-19 vaccination in 2020	0.095	SE:0.040
Viskupič (2023a) [33]	USA (South Dakota); May 2022-February 2023	Convenience sample; n = 243 US adult participants who had been fully vaccinated against COVID-19	9 months from baseline	Controls for age, gender, income and political affiliation	Ordered logistic regression; Beta coefficient	Change in trust in government on attitude towards uptake of an annual COVID-19 vaccine booster	0.418	*p* < 0.05, SE: 0.179
					Logistic regression; Odds ratio	Association of trust in government with COVID-19 booster uptake	1.92	95% CI: (0.7,4.81); p=0.164
						Association of trust in physicians with COVID-19 booster uptake	1.28	95% CI: (1.07,1.54); p=0.008
						Predicted probability of receiving a booster based on a trust in physicians score of 10/25	0.693	
Viskupič (2023b) [34]	USA (South Dakota); April 2021-May 2022	Convenience sample; n= 211 South Dakota registered voters aged 65 or older	12-13 months from baseline	Controls for education, gender, political party identification, receipt of influenza vaccine, evangelical identity and a recent positive COVID-19 test		Predicted probability of receiving a booster based on a trust in physicians score of 15/25	0.849	
					Predicted probability; Percent	Predicted probability of receiving a booster based on a trust in physicians score of 20/25	0.939	
						Predicted probability of receiving a booster based on a trust in physicians score of 25/25	0.979	
						Trust healthcare provider a great deal vs. not on uptake of COVID-19 vaccine: Non-Hispanic Black Detroiters	2.65	95% CI: (2.61,2.70)
						Trust healthcare provider a great deal vs. not on uptake of COVID-19 vaccine: Non-Hispanic White Detroiters	1.45	95% CI: (1.40, 1.49)
Wagner (2022) [31]	USA (Detroit); October 2020-June 2021	Representative sample of the city of Detroit; n = 856 US adult participants in the Detroit Metro Area Communities Study (DMACS)	9 months from baseline	Controls for gender, age, yearly income and education	Multivariable logistic regression; Odds ratio	Trust government health officials a great deal vs. not on uptake of COVID-19 vaccine: Non-Hispanic Black Detroiters	2.84	95% CI: (2.79, 2.89)
						Trust government health officials a great deal vs. not on uptake of COVID-19 vaccine: Non-Hispanic White Detroiters	0.74	95% CI: (0.72, 0.77)
						Association between trust in government and compliance with preventative measures	0.021	95% CI: (0.007, 0.035)
						Association between trust in the healthcare system and compliance with preventative measures	0.003	95% CI: (-0.013, 0.018)
Wright (2021) [40]	UK; March-June 2020	Convenience sample; n= 51,600 UK adults	3 months from baseline	Not reported	Random intercept-cross lagged panel models; Beta coefficient	Association between an increase in trust in government and compliance with preventative measures	0.235	95% CI: (0.209, 0.261)
						Association between an increase in trust in the healthcare system and compliance with preventative measures	0.239	95% CI: (0.207, 0.271)

All but one study included a focus on public health interventions related to the COVID-19 pandemic (*n* = 15), such as attitudes towards/views on and adherence to/uptake of the COVID-19 vaccine (n = 12) and non-pharmaceutical-based prevention measures such as stay-at-home orders, social distancing and masking (*n* = 3). Alongside measuring attitudes towards/uptake of the COVID-19 vaccine specifically, one study also examined attitudes towards/uptake of vaccines in general. The remaining study exclusively examined attitudes towards/uptake of the H1N1 vaccine and adherence to preventative measures during the H1N1 outbreak in 2009.

### Quality of included studies and certainty of evidence

Based on the Cambridge Quality Checklists, the overall quality of the 16 included studies was relatively high [25]. Out of a possible score of 15 points (combining a correlate score out of 5, a risk factor score out of 3, and a causal risk factor score out of 7), five studies scored in the medium-quality range with 8 points (*n* = 1) and 10 points (*n* = 4), respectively, and 11 studies scored on the higher-quality end with 11 (*n* = 9), 12 (*n* = 1) and 13 (*n* = 1) points, respectively. A full breakdown of the quality of each study can be found in Appendix 4. Based on the GRADE approach for certainty of evidence, the confidence in the findings of this review can be considered variable based on the outcome of interest. Of our three outcomes reported below (i.e., attitudes towards/uptake of 1) the COVID-19 vaccine; 2) COVID-19 preventative measures and 3) non-COVID vaccination) certainty in the evidence was determined to be low, low and very low, respectively. A full breakdown of the certainty of evidence for each of our outcomes can be found in Appendix 5.

### Narrative synthesis findings

**Fig. 2:**
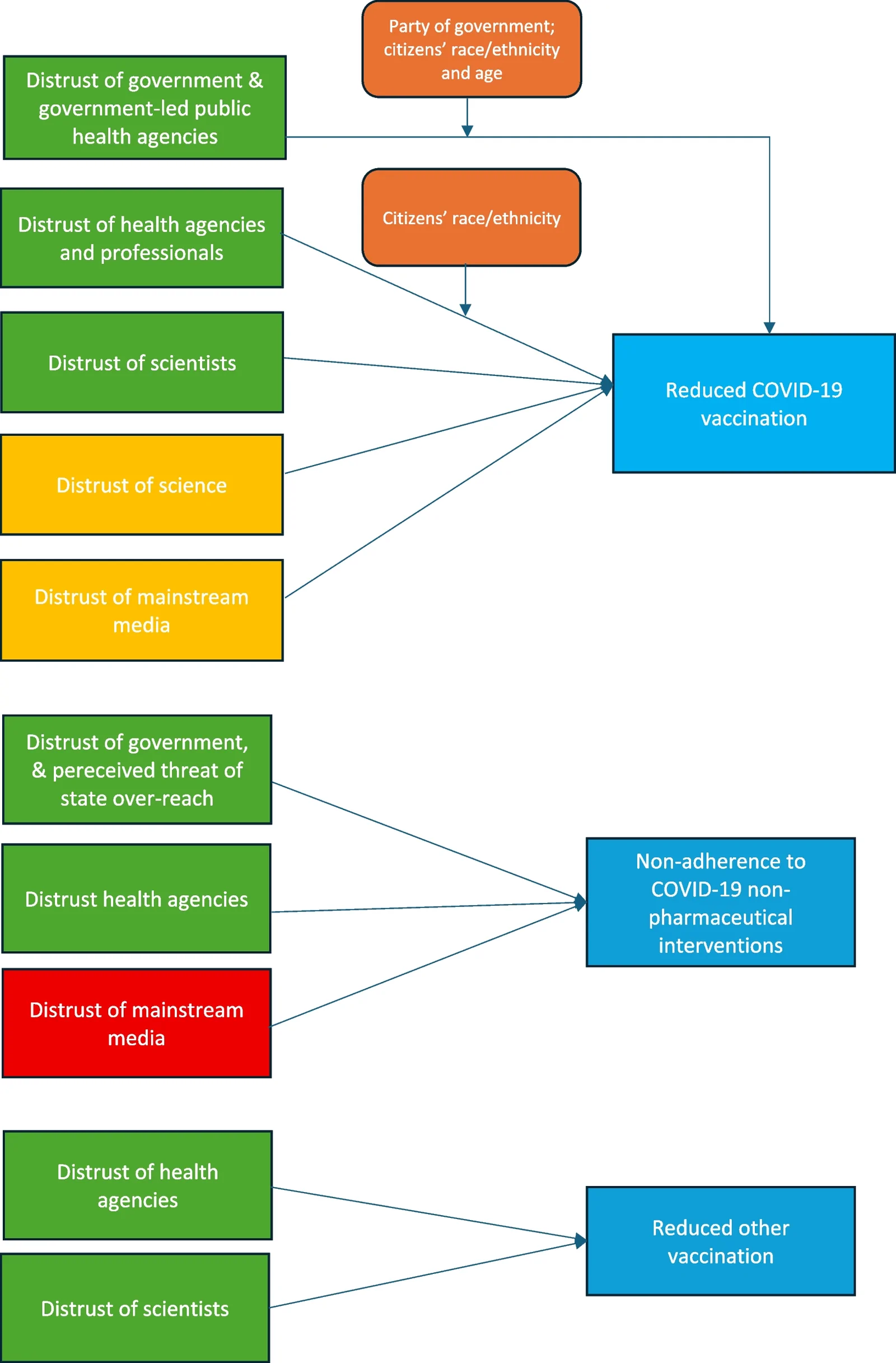
Logic model. Key: green = populist-aligned view with consistent evidence supporting association with outcome; amber: populist-aligned view with mixed evidence supporting association with outcome; red: populist-aligned view with no evidence supporting association with outcome; orange = possible contextual moderator; blue = engagement with public health intervention

The following is a narrative synthesis of the 16 included longitudinal studies. These findings have been organised according to health topic and then the specific populist-aligned view being measured. Within each section, we describe the overall pattern of association presented in the evidence and what this tells us about how populist-aligned views relate to engagement with public health interventions. This is also summarised at the start of our discussion section and, diagrammatically, as a logic model in Fig. 2, which was constructed inductively based on the overall evidence synthesis. Where studies report on more than one populist-aligned view, they are reported in more than one sub-section.

### COVID-19 vaccination

Twelve studies examined the relationship between holding populist-aligned views and later attitudes towards and/or uptake of the COVID-19 vaccine. Among these, several studies examined populist-aligned views in terms of the role that distrust of government, politicians, scientists, medical/health professionals and mass media had on vaccine outcomes, with distrust of at least one of these bodies (government, scientists, mainstream media, or health agencies and professionals) emerging as an important factor in reduced vaccination engagement in all studies.

### Distrust of government and government-led public health institutions

The majority of studies reported temporal associations between populist-aligned views of distrust in government and later negative engagement with COVID-19 vaccination interventions, even among vastly different sample populations. For example, in a high-quality study of 4,803 essential workers in the US by Lutrick et al., increased trust in government in December 2020 was associated with increased odds of COVID-19 vaccination in May 2021 across the whole sample, as compared to not being vaccinated (OR: 4.40, 95% CI: 3.87–5.00) [30]. However, when looking at the sample broken down into one of three groups based on vaccine intentions at baseline, both the ‘reluctant’ and intermediate ‘reachable’ groups were less likely to trust the government (OR: 0.2, 95% CI: 0.17- 0.23; and OR: 0.58, 95% CI: 0.51–0.67, respectively) compared to the vaccine’endorser’ group. It was also found that, for each point increase in trust in government between the December 2020 and May 2021 surveys, there was a 9.4% increase in the likelihood of vaccination across the entire sample (SE: 0.00546, p < 0.01). However, when the sample was broken down by vaccine intention, the effect of an increase in trust in government was higher among the’endorser’ group compared to the’reluctant’ and’reachable’ groups (8.1%, SE: 0.00748, p < 0.01 vs. 6.3%, SE: 0.00815, p < 0.01, respectively). Similar to these findings among essential workers, another high-quality study of 2,562 parents of US children by Rivers et al. found that, for parents of children ages 5–11, compared to being neutral, trust in government in July 2021 was a predictor of COVID-19 vaccination nine months later (AOR: 7.43, 95% CI: 4.65, 11.88) [31]. For parents of children aged 12 and older, again, compared to being neutral, trust in government was also a predictor of COVID-19 vaccination (AOR: 4.74, 95% CI: 2.61, 8.61), while a lack of trust in government was a predictor of no vaccine uptake (AOR: 0.34, 95% CI: 0.18, 0.67). Finally, in a medium-quality study of 90 unhoused adults living in Los Angeles by Kuhn et al., it was found that trust in government sources of COVID-19 information in late December 2020/early January 2021 was associated with less vaccine hesitancy by late February 2021 (AOR: 0.37, 95% CI: 0.12–1.11, *p* = 0 0.08) [32].

Interestingly, in addition to these three studies, for several other studies conducted in the US, there is some evidence to suggest that the direction of the association between trust in government and a subsequent lack of engagement with the COVID-19 vaccine could be affected by whether the party of government was itself populist. For example, in a medium-quality study of 492 US adults by Latkin et al., a high level of trust in state health departments in March 2020 was a predictor of vaccination at the first follow-up survey in June 2021 (AOR: 2.41, 95% CI: 1.37–4.25), while a high level of trust in the Trump White House was associated with reduced odds of vaccination (AOR: 0.42, 95% CI: 0.24- 0.74) [33]. By the second follow-up survey in November 2021, a high level of trust in the Centers for Disease Control (CDC) was a predictor of vaccination (AOR: 2.69, 95% CI: 1.12–6.49), while a high level of trust in the Trump White House was again associated with no vaccination (AOR: 0.24, 95% CI: 0.11–0.51).

Across different study settings, there were also some divergent findings worth noting in terms of the reported associations between distrust in government and reduced engagement with COVID-19 vaccination, contingent on the social identities of participants, including their race/ethnicity and age. For example, in a high-quality study of a representative sample of 856 non-Hispanic White and non-Hispanic Black adults from Detroit, Wagner et al. found that trust in government health officials in October 2020 (versus not trusting these officials) was associated with vaccine uptake among Black participants in June 2021 (OR: 2.84, 95% CI: 2.79–2.89), but associated with decreased uptake among White participants (OR: 0.74, 95% CI: 0.72–0.77) with the interaction of race and the main effect being statistically significant (p < 0.0001) [34]. Mediation analysis found that trust explained 3% of the difference in vaccine uptake between White and Black participants; increasing the level of Black participants’ trust in government health officials to that of White participants was estimated to enable the elimination of 18% of the difference in vaccine uptake. In contrast to Wagner et al.’s finding of an association for Black but not White participants in the US, a medium-quality study among 633 UK ethnic-minority adults by Paul et al. found that there was no significant difference in vaccine refusal in June 2021 among participants expressing low confidence in the UK government in December 2020, which might point to the role of political or other contexts in these relationships [35].

When it came to the influence of age, in a high-quality study by Viskupic et al. of 211 senior voters in South Dakota, the authors measured the relationship between trust in government in April 2021 and uptake of the COVID-19 booster in April/May 2022 [36]. Here, trust in government was not associated with booster uptake, though it is noted that this may be reflective of older people’s greater motivation to receive boosters given their generally greater susceptibility to severe COVID-19 symptoms.

The association between changes in trust in government over time was explored in relation to attitudes towards/uptake of COVID-19 vaccines in two studies, providing evidence of the negative role of ongoing or increasing distrust in the government over time. In the first, a medium-quality study of 243 fully vaccinated US adults conducted by Viskupic et al., positive changes in individuals’ trust in government between May 2022 and February 2023 were associated with willingness to receive an annual COVID-19 booster in February 2023 (β = 0.418, SE: 0.179, *p* < 0.05) [37]. In the second, a high-quality study of a representative sample of 80,906 French adults by Spire et al. (2023), the authors measured the effect of a change in trust in government between November 2020 and July 2021 on the probability of being vaccinated by July 2021 [38]. Participants at baseline were categorised into one of three groups:’undecided’,’vaccine reluctant’, and’vaccine acceptant’. Among the’undecided’ group, compared to those who continued not to trust the government, those who continued to trust the government or began to trust the government were more likely to be vaccinated at follow-up (OR: 1.71, 95% CI: 1.5, 1.95; and OR: 1.69, 95% CI: 1.46, 1.94, respectively), while those who lost trust in the government were less likely to be vaccinated (OR: 0.82, 95% CI: 0.71, 0.94). Among the ‘vaccine-reluctant’ group, compared to those who continued not to trust the government, those who continued to trust the government or began trusting the government were more likely to be vaccinated at follow-up (OR: 2.12, 95% CI: 1.94, 2.33; and OR: 2.14, 95% CI: 1.94, 2.36, respectively), while those who lost trust in government were less likely to be vaccinated (OR: 0.88, 95% CI: 0.8, 0.97). Finally, among the ‘vaccine-acceptant’ group, compared to those who continued not trust the government, those who lost trust in government were more likely not to be vaccinated (OR: 1.14, 95% CI: 1.04, 1.25), while those who either continued to trust the government or began to trust the government were less likely not to be vaccinated (OR: 0.55, 95% CI: 0.51, 0.60; and OR: 0.56, 95% CI: 0.51, 0.61, respectively).

### Distrust of scientists and science

We now turn to the role of populist-aligned views concerned with prior distrust of scientists and science in the subsequent receipt of the COVID-19 vaccine. Findings here were more mixed and appeared to be more dependent on country context, including evidence that distrust of scientists and/or science was more strongly associated with reduced vaccine engagement in countries with sharper socio-economic inequalities and less social cohesion. For example, in a high-quality study by Galasso et al. [53] using representative samples of adults in Australia, Austria, France, Germany, Italy, New Zealand, Sweden, UK and USA, overall, increased trust in scientists was associated with increased vaccination intentions in December 2020 and actual vaccine uptake in June/July 2021 (0.127, SE: 0.01, *p* < 0.01; and 0.033, SE: 0.020, *p* < 0.10, respectively) [39]. At the country level, the UK and USA showed the strongest associations between trust and vaccination intentions (0.175, SE: 0.030, *p* < 0.01), followed by Australia and New Zealand (0.121, SE: 0.030, *p* < 0.01), and finally EU countries (0.118, SE: 0.012, *p* < 0.01). Regarding the relationship between increased trust in scientists in December 2020 and actual vaccine uptake in June/July 20,201, however, only the UK, USA, Australia and New Zealand had higher trust as a predictor of uptake (0.089, SE: 0.023, *p* < 0.01; and 0.076, SE: 0.035, *p* < 0.05, respectively).

The study by Galasso et al. [53] also used participants’ vaccine-intention scores in December 2020 to identify those in each population exhibiting anti-vaccine attitudes [39]. They then isolated this sub-sample in a regression analysis to unpack the drivers of anti-vaccine intentions at baseline, where they found that less trust in scientists was associated with lower vaccination intentions in December 2020 for those categorised as ‘anti-vaccine’ across all countries (−0.156, SE: 0.016, *p* < 0.01), though this negative effect was more pronounced among those with ‘hard’ versus ‘soft’ anti-vaccine views (−0.132, SE: 0.013, *p* < 0.01; and −0.024, SE: 0.012, *p* < 0.10, respectively). The negative association between lower trust in scientists and vaccination intentions in December 2020 among individuals identified as having anti-vaccine attitudes was also seen at the individual country level, with the UK and USA showing the strongest association (−0.203, SE: 0.041, p < 0.01), followed by EU countries (−0.152, SE: 0.019, *p* < 0.01), and finally Australia and New Zealand (−0.128, SE: 0.020, *p* < 0.10). Lastly, the authors found that greater trust in scientists among those with anti-vaccine attitudes was associated with greater vaccine uptake when all countries were pooled together, (0.064, SE: 0.029, *p* < 0.05), though at the individual country level this was only true for Australia and New Zealand (0.113, SE: 0.042, *p* < 0.05).

The importance of trust in scientific bodies for future vaccine uptake found in Galasso et al.’s multi-country study was partially supported in a previously mentioned study of 492 US adults by Latkin et al., where it was found that March 2020 levels of trust in a leading public health research institution were associated with COVID-19 vaccination in June and November 2021 in unadjusted models (OR: 4.34, 95% CI: 2.58, 7.30; and OR: 5.19, 95% CI: 2.81, 9.57, respectively). However, this significant association disappeared in adjusted models [33]. Elsewhere, in a medium-quality study of 400 Polish adult internet users by Stasiuk et al., the authors examined the association of trust in science in February 2018 on attitudes towards COVID-19 vaccination in December 2020 [40], finding no significant association between trust in science at the pre-pandemic baseline and attitudes towards COVID-19 vaccination two years later.

Two studies examined the role of changes in trust in scientists over time and receipt of the COVID-19 vaccine, where, as was seen with government trust, it was generally found that ongoing and increasing distrust over time had a negative influence on vaccine uptake. In the first study, using a representative sample of 918 Polish adults, Maciuszek et al. measured differences in group means in terms of intention to vaccinate in February 2021 and actual vaccination in August 2021, based on trust in science at each timepoint [41]. Trust in science was highest among those who were both willing to vaccinate in February 2021 and vaccinated in August 2021, and lowest among those who were unwilling to vaccinate in February 2021 and unvaccinated in August 2021 (M = 4.03, standard deviation (SD) = 0.67 versus highest M = 3.43, SD = 0.9; *p* = 0.005). There was an increase in trust in science between February and August among those consistently willing to be vaccinated (M = 3.98, SD = 0.65 vs. M = 4.03, SD = 0.67, t(378) = 1.990, *p* = 0.047), and a decrease in trust in science among those who moved from being undecided in February to being unvaccinated in August (M = 3.70, SD = 0.76 vs. M = 3.53, SD = 0.80, t(29) = 2.430, *p* = 0.022). In the second study, based on a previously mentioned nationally representative sample of 80,906 French adults, Spire et al. similarly measured the effect of a change in trust in scientists between November 2020 and July 2021 on the probability of being vaccinated by July 2021 [38]. Among those who reported being undecided about the vaccine at baseline, compared to those who continued not to trust scientists between baseline and follow-up, both those who continued to trust scientists and those who began to trust scientists were more likely to be vaccinated (OR: 1.83, 95% CI: 1.46, 2.3; and OR: 1.91, 95% CI: 1.51, 2.43, respectively). Among those who identified as being reluctant to be vaccinated at baseline, compared to those who continued not to trust scientists, both those who continued to trust scientists and those who began to trust scientists were more likely to be vaccinated (OR: 2.06, 95% CI: 1.84, 2.31; and OR: 1.93, 95% CI: 1.7, 2.17, respectively). Finally, among the group who identified as vaccine acceptant at baseline, compared to those who continued not to trust scientists, those who either continued to trust scientists or began to trust scientists between baseline and follow-up were less likely to be unvaccinated (OR: 0.46, 95% CI: 0.39, 0.54; and OR: 0.54, 95% CI: 0.45, 0.65, respectively).

### Distrust of mainstream media

Only two of the included studies evaluated the role of populist-aligned views related to prior distrust of mainstream media as a source of information on COVID-19 and subsequent receipt of the COVID-19 vaccine, reporting mixed results. In the first, a previously mentioned study of 492 US adults by Latkin et al., trust in major news outlets in March 2020 predicted vaccination by June 2021 (AOR: 2.03, 95% CI: 1.26–3.27) and by November 2021 (AOR: 3.54, 95% CI: 1.82–6.92) [33]. In the second, another previously mentioned study of 90 unhoused adults living in Los Angeles by Kuhn et al., there was no association between trust in traditional media for COVID-19 information as measured in late December 2020/early January 2021 and vaccine hesitancy by late February 2021 [32]. This divergent finding might be explained by reduced access to media among unhoused people so that distrust of mainstream media is associated with reduced vaccination but only among those groups with access to these media.

### Distrust of medical/health professionals

The final aspect of populist-aligned views related to distrust of elite institutions, which was examined in included studies in terms of the role of distrust of medical and/or health professionals with regard to COVID-19 vaccination. Across different study populations and settings, there was evidence to support temporal associations between distrust in these professionals and reduced engagement with vaccination, particularly among those with minoritised group identities and older adults. For example, in a previously mentioned study of 633 UK adults belonging to ethnic-minority groups, Paul et al. found that, compared to those with high confidence in the health service, those with low confidence were more likely to refuse the COVID-19 vaccine from December 2020 through to June 2021 (OR: 7.51, 95% CI: 2.05–27.43) [35]. Similarly, in another previously mentioned study based on a representative sample of 856 non-Hispanic White and non-Hispanic Black adults from Detroit, Wagner et al. found that greater trust in healthcare providers (compared to having no trust) in October 2020 was associated with higher vaccine uptake in June 2021 among both White and Black participants, although this association was stronger among Black compared to White participants (OR: 2.65, 95% CI: 2.61–2.70 versus OR: 1.45 95% CI: 1.40–1.49, respectively), with the interaction being significant (*p* < 0.0001) [34]. Furthermore, mediation analysis revealed that trust in healthcare providers explained 6% of the difference in vaccine uptake between these groups, with 23% of the difference in uptake being potentially eliminated if Black participants were to have the same level of trust as White participants. In another previously mentioned study of 211 senior voters in South Dakota by Viskupic et al., higher levels of trust in physicians in April 2021 increased the likelihood of having a COVID-19 booster vaccine in May 2022 (OR: 1.28, 95% CI: 1.07,1.54, *p* = 0.008) [36]. The authors also found that higher trust in physicians was associated with higher probabilities of booster uptake (score of 10/25 = 0.693; 15/25 = 0.849; 20/25 = 0.939; and 25/25 = 0.979). Finally, in the aforementioned study of 400 Polish internet-using adults by Stasiuk et al., it was found that trust in physicians in 2018 was associated with more positive attitudes towards vaccination in late 2020 (β = 0.23, SE: 0.042; *p* < 0.05) [40].

## Non-pharmaceutical-based COVID-19 preventative measures

### Distrust of government, healthcare, scientists and media

Three studies assessed the role of populist-aligned attitudes on subsequent perceptions of, and adherence to, non-pharmaceutical-based preventative measures during the pandemic, with findings generally suggestive of an association between populist-aligned views in the form of distrust of government and science, as well as concerns about government authoritarianism, and less support for non-pharmaceutical interventions.

Among these were two studies examining the role of distrust of the government, scientists and the media on engagement with these preventative measures. In the first, a high-quality study of 214 German and Polish adults by Ehrke et al., the authors found that trust in either mainstream or alternative media at baseline (May 2020) had no significant association with attitudes towards or adherence to preventative measures in June 2020, while the total effect of holding populist views in April 2020 was associated with a decrease in positive subsequent attitudes towards prevention (β = −0.2, *p* = 0.007), and increased trust in political and scientific institutions was associated with more positive subsequent attitudes towards prevention measures (β = 0.34, *p* = 0.009; 0.27, β = 0.27, *p* = 0.001, respectively) [42]. When measures of trust in political and scientific institutions were added as mediators in the relationship between populist views and attitudes towards preventative measures, however, this resulted in less positive attitudes towards preventative measures (β = −0.119, Boot SE: 0.049, 95% CI: –0.222, –0.030; β = −0.049, Boot SE: 0.020, 95% CI: –0.091, –0.013, respectively). Finally, in terms of adherence to preventative measures, while populist attitudes did not have a direct association with this outcome, trust in political and scientific institutions were both associated with higher adherence (β = 0.02, *p* = 0.013; β = 0.35, *p* < 0.001, respectively), while only trust in scientific institutions was negatively associated with adherence when used as a mediator in the relationship between holding populist views and this outcome (β = −0.063, Boot SE: 0.025, 95% CI: –0.116, –0.018). In the second, a similarly high-quality study of 51,600 UK adults by Wright et al., the authors measured the association of trust in government and the healthcare system in March 2020 with self-reported adherence to COVID-19 guidelines in June 2020, finding small yet significant associations (β = 0.021, 95% CI: 0.007- 0.035; and β = 0.003, 95% CI: −0.013—0.018, respectively) [43]. They also found an association between an increase in trust in government between baseline and follow-up and compliance with preventative measures at follow-up (β = 0.235, 95% CI: 0.209—0.261), though no such association was found for an increase in trust in the healthcare system and compliance.

Finally, although limited to one study, there was some evidence to suggest that perceptions of state over-reach were associated with subsequent views on and adherence to preventative measures. Specifically, in a high-quality study of 1,130 Polish adults by Oleksy et al., while the authors found no significant association between trust in government and adherence to preventative behaviours using data from May to December 2020, they did find a reciprocal, bidirectional association between a fear that pandemic restrictions to citizens’ rights and freedoms would be made permanent and preventative behaviours (within-subject relationship between these fears and preventative behaviours: β = −0.074, SE: 0.02, *p* < 0.001; within-subject relationship between preventative behaviours and these fears: β = −0.235, SE: 0.085, p = 0.006) [44]. This finding indicates that changes in adherence over time were negatively predicted by perceptions about perceived threat of authoritarianism, and vice versa.

### Other vaccination

Only two studies included an examination of the role of populist-aligned attitudes in the receipt of interventions not related to COVID-19, both finding associations between holding populist-aligned views related to distrust of elite institutions and/or actors and negative engagement with vaccination other than COVID-19. In the first, a high-quality, two-wave longitudinal survey of 601 French-speaking Swiss adults conducted during the 2009 H1N1 outbreak[45], trust in medical organisations was found to be associated with H1N1 vaccination six months later (β = 0.76, SE: SE: 0.21, p < 0.001), as well as having positive perceptions about the efficacy of preventative measures such as the H1N1 vaccine (β = 0.3, SE: 0.08, *p* < 0.001), handwashing (β = 0.17, SE: 0.06, *p* < 0.01) and wearing a mask (β = 0.22, SE: 0.08, p < 0.01) [45]. In the second, a previously mentioned study of 400 Polish internet-using adults by Stasiuk et al., it was found that changes in trust in both physicians and science between 2018 and 2020 were associated with similar changes in attitudes towards vaccination generally, meaning as trust in either physicians or science decreased, so did positive attitudes towards vaccination, while increases in trust in either entity meant similar increases in positive attitudes towards vaccination (change in trust in physicians: β = 0.244, SE: SE: 0.059; p < 0.001; change in trust in science: β = 0.288, SE: 0.058; p < 0.001) [40].

## Discussion

### Summary of key findings

To our knowledge, this study is the first to synthesise longitudinal evidence related to the temporal relationship between the prior holding of populist-aligned views and subsequent engagement with public health interventions, as well as the first to explore the potential public health implications of an array of individual-level views that might not be part of an explicit populist identity or worldview, but generally align with contemporary populist discourse. In line with previous findings from our syntheses of qualitative and cross-sectional evidence, the findings from this synthesis of quantitative, longitudinal evidence broadly support the idea that among the populist-aligned views examined, but especially those related to distrust of government, scientists and medical and/or health professionals, there is a negative relationship between holding these views and engagement with public health interventions addressing COVID-19 [17, 18]. Our review also provides preliminary indications that these associations involve populist-aligned attitudes being temporally prior to intervention receipt. These associations, along with potential moderators/mediators, are summarised for each of the public health interventions for which there is currently longitudinal evidence as a logic model in Fig. 2. Note that rather than a definitive view of what matters in terms of engagement with these public health interventions, this logic model should be viewed simply as a depiction of the current state of evidence.

In terms of the influence on specific populist-aligned views, when it comes to distrust of government and COVID-19 vaccine uptake, the evidence generally seems to be particularly strong related to an association with reduced engagement with vaccination. However, in some cases, the direction of this association appears to be dependent on the party in government and whether the leader could themselves be considered populist. For example, in US studies, when trust was measured with President Trump in office, the association between this and later vaccine acceptance and/or uptake was negative, while in studies conducted when President Biden was in office, the association was positive [31, 33, 37, 46].

Several experimental studies support this finding related to the influence of President Trump in particular on vaccine uptake. For example, in a set of pre-pandemic studies conducted among Trump voters by Hornsey et al., not only were these individuals more worried about vaccination than other Americans, but exposing participants to President Trump’s anti-vaccine social media posts significantly increased their vaccine concerns compared to a control group exposed to his posts about golf [47]. The authors conclude from these findings that there is potential for leaders such as President Trump to not only represent the attitudes and opinions of their supporters, but also to change these attitudes and opinions, something that is supported by later studies on the positive impact of President Trump’s COVID-19 vaccine endorsement among Republicans in 2021 [48, 49].

Our review also found preliminary, though limited, evidence that the association between populist-aligned distrust of government and reduced engagement with vaccination may be contingent on citizens’ social identities, and particularly related to their race/ethnicity and age. Unfortunately, the pattern found with the former could not be generalised across settings given that one study suggested distrust eroded vaccine engagement among Black participants in the US while another had no such finding among ethnic-minority participants in the UK [34, 35]. Similarly, regarding the role of age, it is difficult to make generalisations based on only one study that looked at this characteristic, where it was found that distrust was not associated with reduced vaccine engagement among older participants, perhaps because of greater overall motivation to be vaccinated in this group [36].

Across most of the studies that explored the association between populist-aligned attitudes and COVID-19 vaccination, evidence suggests that individually-held views related to distrust of scientists, as well as health agencies and professionals before and throughout vaccine roll-out were important in vaccine hesitancy and reduced uptake, with the influence of distrust in health agencies and professionals especially salient among minoritised groups [33–36, 38–40, 46]. Regarding trust in scientists, research conducted across multiple regions by Galasso et al. lends preliminary support to the idea that such populist-aligned attitudes could be more likely to undermine vaccine uptake in countries where there is greater socioeconomic inequality and less social cohesion [39, 50]. One potential explanation for this is that it could reflect more polarised or anti-elite cultural values in more fractured polities [15]. More research is needed to examine this theory, however, and in particular the ways in which trust in scientists applies to the vaccine intentions of individuals who are already explicitly anti-vaccine in settings with greater socioeconomic inequality.

Other areas that proved inconclusive and thus requiring of further research include the role of populist-aligned distrust in science overall, as well as distrust in mainstream media and subsequent COVID-19 vaccine uptake. Regarding trust in science overall, current evidence is limited to the contradictory findings of two Polish studies, the first finding an association between trust in science at baseline and COVID-19 vaccine uptake and the second finding no such association [40, 41].Regarding trust in mainstream media and orientation to COVID-19 vaccination, there were mixed findings with evidence of an association between trust and vaccine uptake among a general US population but no findings of an association with vaccine hesitancy among an unhoused US population. This latter divergence might be explained by reduced media access among unhoused people.

Related to the role of populist-aligned attitudes in the receipt of non-pharmaceutical-based COVID-19 preventative measures, while only three studies examined this association, the limited available evidence generally supports that distrust of government, the healthcare system and scientific institutions are all associated with reduced acceptance of, and adherence to, preventative measures [42–44]. There is also some indication that increasing levels of trust over time may also have a positive influence on adherence, while distrust in science, anti-elite views and perceptions of government overreach are associated with lower levels of adherence [42–44]. Although this latter point is supported by cross-sectional studies carried out during the pandemic in western Europe, this is another area that will require further research in order to confirm temporally [51, 52]. As with COVID-19 vaccination, there was no conclusive evidence that trust in mainstream media was associated with more positive attitudes and adherence to preventative measures.

Finally, in the two studies that included non-COVID-19 related vaccination, while the limited available evidence points to the importance of existing and increasing levels of trust in public health organisations, pharmaceutical companies, medical professionals and science in general in promoting later acceptance and uptake of the H1N1 vaccine and vaccines in general, more research is needed to reach a firm conclusion beyond these two studies [40, 45].

### Limitations

This synthesis of longitudinal evidence should be viewed in light of several important limitations. First, there was a strong focus on COVID-19-related interventions among the available evidence, as well as on populist-aligned views related to distrust in elite institutions and/or actors, and to a much lesser extent, checks on popular sovereignty. Although we think it is plausible that some of the temporal associations between these populist-aligned views and reduced engagement with public health interventions might be generalisable to other health topics, this is not certain given the evidence we present. Further research is required that expands our understanding of the temporal associations between populist-aligned views and other health topics. Within this, additional research is also needed that includes measurement of other key aspects of populism and populist discourse, such as hostility towards elites viewed as promoting checks on popular sovereignty, unwelcome social change, or the interests of out-groups, as well as hostility to these out-groups themselves [9–12].

A second potential limitation that might be raised is conceptual, concerning how we defined the views that we examined, but is one that we would reject. We decided to include studies based on the measurement of specific views, which, informed by the broader literature on populism, could reasonably be conceived as aligning with populist discourses. We hypothesised that, even though each person might not hold all such views and might not themselves identify them as populist, these views nonetheless all resonate with, and are likely influenced byl broader populist discourse, and all reduce engagement with public health interventions [9]. Focusing on these specific views regardless of whether they were labelled as populist by study participants or authors also provided a consistent standard for inclusion and avoided biasing selection towards studies taking a particular perspective on populism [15]. While this might mean that not all of the views included in this synthesis perfectly represent a populist worldview, our overall finding that various measures aligning with different aspects of populism tended to be associated with reduced engagement with public health interventions does suggest that it was useful to bring together the evidence in this way.

A third limitation lies in the types of evidence we could synthesise. Studies were highly heterogeneous in terms of what populations were involved, what phase in the COVID-19 pandemic data collection occurred in, what populist-aligned views were measured and what aspects of intervention receipt measures were assessed. This together with only 16 studies being included precluded statistical meta-analysis or meta-regression. There was also an added limitation in the quality of the evidence which meant that although we could explore the temporality of associations, we could not determine whether these were causal.

## Conclusions

### Implications for research and policy

In line with the above limitations, future research on this topic should aim to expand the current understanding of the influence of a variety of populist-aligned attitudes on receipt of public health interventions beyond those related to COVID-19. In the next phase of our research, we will conduct primary qualitative research exploring how populist-aligned views are implicated in accounts of engagement with public health interventions beyond those related to COVID-19. Furthermore, given the lack of conclusive evidence on the association of trust in elite bodies such as the mainstream media and science more broadly with intervention engagement, future research is also needed to unpack how this shapes engagement with public health interventions among different populations. Lastly, further research is needed to examine the causal relationship between holding populist-aligned views and a reduction in positive response to specific public health interventions, and to identify the mechanisms that can be leveraged to increase acceptability and uptake among those holding populist-aligned views, ideally through experimental studies.

In terms of implications for policy, despite most of our evidence relating to COVID-19 interventions, our findings suggest the importance of building citizens’ trust in the political, scientific and medical establishments prior to and throughout the implementation of any public health intervention (53). For political and scientific actors, this could be done through engagement with populations that are more likely to have feelings of distrust, as well as targeted messaging and communications across various forms of media aimed at overcoming distrust and assuaging potential fears about government overreach based on government-led public health policies. For medical and health professionals, this could be done through increased training in methods such as motivational interviewing (54). This is a behaviour change approach rooted in an understanding that individuals can be at different stages of motivation in terms of modifying their behaviour and, as such, efforts need to be made to help the individual resolve any existing uncertainties in order to increase their motivation to participate in a public health intervention (55). Although any application of our findings to other areas of public health is of course speculative, we suggest that such actions would be prudent given the manifest prevalence and influence of populist views among much of the population. This is particularly the case for those citizens with entrenched distrust of government or other’elite’-led public health interventions. Though our evidence supports the role of trust particularly in the context of public health emergencies such as the COVID-19 pandemic, by creating space for dialogue and relationship-building between elite groups and the wider public, it may be possible to tackle some of the core components of populist-aligned grievances that manifest as in opposition towards public health interventions.

## Supplementary Information

Supplementary Material 1.

## Data Availability

The datasets used and/or analysed during the current study are available from the corresponding author on reasonable request.

## Abbreviations

AMK: Alison McKinlay
AOR: Adjusted Odds Ratio
BeSST: Behavioural and Social Sciences Team
CBo: Chris Bonell
CDC: Centers for Disease Control and Prevention
DEI: Diversity, Equity and Inclusion
DHSC: Department of Health and Social Care
FG: Fiona Graham
GRADE: Grading of Recommendations, Assessment, Development, and Evaluations
JB: Jack Birch
KCM: Kaitlin Conway-Moore
MESH: Medical Subject Headings
NIHR: National Institute for Health and Care Research
OECD: Organisation for Economic Co-operation and Development
OHID: Office for Health Improvement and Disparities
OR: Odds Ratio
PPIE: Patient and Public Involvement and Engagement
PRISMA: Preferred Reporting Items for Systematic Reviews and Meta-Analyses
SD: Standard Deviation
SE: Standard Error
USA: United States of America
WHO: World Health Organization
