# Supplementary material for: The association between populist-aligned views and engagement with public health interventions: a systematic review of longitudinal studies
Source: BMC Public Health. 2026 Jun 16;26:2421. doi: 10.1186/s12889-026-28020-w (PMC13501592; doi:10.1186/s12889-026-28020-w)
Supplement: Supplementary file 1 [file 12889_2026_28020_MOESM1_ESM.docx]

# Appendix 1: PRISMA 2020 Checklist^112^

| **Section and Topic** | **Item #** | **Checklist item** | **Location where item is reported** |
| --- | --- | --- | --- |
| **TITLE** | | |  |
| Title | 1 | Identify the report as a systematic review. | 1 |
| **ABSTRACT** | | |  |
| Abstract | 2 | See the PRISMA 2020 for Abstracts checklist. | 2 |
| **INTRODUCTION** | | |  |
| Rationale | 3 | Describe the rationale for the review in the context of existing knowledge. | 6 |
| Objectives | 4 | Provide an explicit statement of the objective(s) or question(s) the review addresses. | 6 |
| **METHODS** | | |  |
| Eligibility criteria | 5 | Specify the inclusion and exclusion criteria for the review and how studies were grouped for the syntheses. | 7-8 |
| Information sources | 6 | Specify all databases, registers, websites, organisations, reference lists and other sources searched or consulted to identify studies. Specify the date when each source was last searched or consulted. | 8-10 |
| Search strategy | 7 | Present the full search strategies for all databases, registers and websites, including any filters and limits used. | 8-10, 4-21 in Suppl Material |
| Selection process | 8 | Specify the methods used to decide whether a study met the inclusion criteria of the review, including how many reviewers screened each record and each report retrieved, whether they worked independently, and if applicable, details of automation tools used in the process. | 10 |
| Data collection process | 9 | Specify the methods used to collect data from reports, including how many reviewers collected data from each report, whether they worked independently, any processes for obtaining or confirming data from study investigators, and if applicable, details of automation tools used in the process. | 10-11 |
| Data items | 10a | List and define all outcomes for which data were sought. Specify whether all results that were compatible with each outcome domain in each study were sought (e.g. for all measures, time points, analyses), and if not, the methods used to decide which results to collect. | 10-11 |
|  | 10b | List and define all other variables for which data were sought (e.g. participant and intervention characteristics, funding sources). Describe any assumptions made about any missing or unclear information. | 10-11 |
| Study risk of bias assessment | 11 | Specify the methods used to assess risk of bias in the included studies, including details of the tool(s) used, how many reviewers assessed each study and whether they worked independently, and if applicable, details of automation tools used in the process. | 11-12 |
| Effect measures | 12 | Specify for each outcome the effect measure(s) (e.g. risk ratio, mean difference) used in the synthesis or presentation of results. | 12-13 |
| Synthesis methods | 13a | Describe the processes used to decide which studies were eligible for each synthesis (e.g. tabulating the study intervention characteristics and comparing against the planned groups for each synthesis (item #5)). | N/A |
|  | 13b | Describe any methods required to prepare the data for presentation or synthesis, such as handling of missing summary statistics, or data conversions. | N/A |
|  | 13c | Describe any methods used to tabulate or visually display results of individual studies and syntheses. | N/A |
|  | 13d | Describe any methods used to synthesize results and provide a rationale for the choice(s). If meta-analysis was performed, describe the model(s), method(s) to identify the presence and extent of statistical heterogeneity, and software package(s) used. | N/A |
|  | 13e | Describe any methods used to explore possible causes of heterogeneity among study results (e.g. subgroup analysis, meta-regression). | N/A |
|  | 13f | Describe any sensitivity analyses conducted to assess robustness of the synthesized results. | N/A |
| Reporting bias assessment | 14 | Describe any methods used to assess risk of bias due to missing results in a synthesis (arising from reporting biases). | N/A |
| Certainty assessment | 15 | Describe any methods used to assess certainty (or confidence) in the body of evidence for an outcome. | 11-12 |
| **RESULTS** | | |  |
| Study selection | 16a | Describe the results of the search and selection process, from the number of records identified in the search to the number of studies included in the review, ideally using a flow diagram. | 14 |
|  | 16b | Cite studies that might appear to meet the inclusion criteria, but which were excluded, and explain why they were excluded. | 22-58 in Suppl Material |
| Study characteristics | 17 | Cite each included study and present its characteristics. | 14-15; 41-57 |
| Risk of bias in studies | 18 | Present assessments of risk of bias for each included study. | 59-60 in Suppl Material |
| Results of individual studies | 19 | For all outcomes, present, for each study: (a) summary statistics for each group (where appropriate) and (b) an effect estimate and its precision (e.g. confidence/credible interval), ideally using structured tables or plots. | 14-28; 41-57 |
| Results of syntheses | 20a | For each synthesis, briefly summarise the characteristics and risk of bias among contributing studies. | 15-16 |
|  | 20b | Present results of all statistical syntheses conducted. If meta-analysis was done, present for each the summary estimate and its precision (e.g. confidence/credible interval) and measures of statistical heterogeneity. If comparing groups, describe the direction of the effect. | N/A |
|  | 20c | Present results of all investigations of possible causes of heterogeneity among study results. | N/A |
|  | 20d | Present results of all sensitivity analyses conducted to assess the robustness of the synthesized results. | N/A |
| Reporting biases | 21 | Present assessments of risk of bias due to missing results (arising from reporting biases) for each synthesis assessed. | N/A |
| Certainty of evidence | 22 | Present assessments of certainty (or confidence) in the body of evidence for each outcome assessed. | 61-62 in Suppl Material |
| **DISCUSSION** | | |  |
| Discussion | 23a | Provide a general interpretation of the results in the context of other evidence. | 29-32 |
|  | 23b | Discuss any limitations of the evidence included in the review. | 33-34 |
|  | 23c | Discuss any limitations of the review processes used. | 33-34 |
|  | 23d | Discuss implications of the results for practice, policy, and future research. | 34-36 |
| **OTHER INFORMATION** | | |  |
| Registration and protocol | 24a | Provide registration information for the review, including register name and registration number, or state that the review was not registered. | 7 |
|  | 24b | Indicate where the review protocol can be accessed, or state that a protocol was not prepared. | 7 |
|  | 24c | Describe and explain any amendments to information provided at registration or in the protocol. | N/A |
| Support | 25 | Describe sources of financial or non-financial support for the review, and the role of the funders or sponsors in the review. | 37 |
| Competing interests | 26 | Declare any competing interests of review authors. | 37 |
| Availability of data, code and other materials | 27 | Report which of the following are publicly available and where they can be found: template data collection forms; data extracted from included studies; data used for all analyses; analytic code; any other materials used in the review. | 37 |

PRISMA=Preferred Reporting Items for Systematic Reviews and Meta-Analyses.

# Appendix 2: Database Search Strategies

## CINAHL

| **Database name** | CINAHL |
| --- | --- |
| **Database platform** | https://www.ebsco.com/products/research-databases/cinahl-database |
| **Dates of database coverage** | 1937-present |
| **Date searched** | 19 February 2024 |
| **Number of results** | 4,438 |

| **#** | **Query** |
| --- | --- |
| S1 | Populis* |
| S2 | Illiberal* |
| S3 | Anti-liberal* |
| S4 | Antiliberal* |
| S5 | Anti-right* |
| S6 | Antiright* |
| S7 | Hostil* N2 right* |
| S8 | Hostil* N2 liberal* |
| S9 | Anti-EDI* |
| S10 | Hostil* N2 EDI* |
| S11 | Anti-equalit* |
| S12 | Antiequalit* |
| S13 | Hostil* N2 equalit* |
| S14 | Anti-divers* |
| S15 | Antidivers* |
| S16 | Hostil* N2 divers* |
| S17 | Anti-inclu* |
| S18 | Anti-intervent* |
| S19 | Hostil* N2 intervent* |
| S20 | Anti-regulat* |
| S21 | Antiregulat* |
| S22 | Hostil* N2 regulat* |
| S23 | Hostil* N2 inclu* |
| S24 | Racism |
| S25 | Racis* |
| S26 | Scapegoating |
| S27 | Xenophobia |
| S28 | Sexism |
| S29 | Elitis* |
| S30 | "Sovereignty" |
| S31 | Nationalis* |
| S32 | Nativis* |
| S33 | Pro-nativis* |
| S34 | Pronativis* |
| S35 | Anti-immigra* |
| S36 | Hostil* N2 immigra* |
| S37 | Anti-migra* |
| S38 | Antimigra* |
| S39 | Hostil* N2 migra* |
| S40 | Anti-minorit* |
| S41 | Antiminorit* |
| S42 | Hostil* N2 minorit* |
| S43 | Anti-ethnic* |
| S44 | Antiethnic* |
| S45 | Hostil* N2 ethnic* |
| S46 | Hostil* N2 raci* |
| S47 | Homophobia |
| S48 | Homophobi* |
| S49 | Transphobi* |
| S50 | Anti-globali* |
| S51 | Antiglobali* |
| S52 | Hostil* N2 globali* |
| S53 | Hostil* N2 wealth* |
| S54 | "Anti-cosmopolitan" |
| S55 | "Anticosmopolitan" |
| S56 | "Far-right" |
| S57 | "Anti-establishment" |
| S58 | "Antiestablishment" |
| S59 | Hostil* N2 establishment* |
| S60 | Trust* N2 establishment* |
| S61 | "Brexit" |
| S62 | Anti-expert* |
| S63 | Antiexpert* |
| S64 | Hostil* N2 expert* |
| S65 | Trust* N2 expert* |
| S66 | Anti-scien* |
| S67 | Antiscien* |
| S68 | Hostil* N2 scien* |
| S69 | Trust* N2 scient* |
| S70 | Hostil* N2 research* |
| S71 | Trust* N2 research* |
| S72 | Anti-business* |
| S73 | Antibusiness* |
| S74 | Hostil* N2 "business" |
| S75 | Trust* N2 "business" |
| S76 | "Anti-government" |
| S77 | "Antigovernment" |
| S78 | Hostil* N2 "government" |
| S79 | Trust* N2 "government" |
| S80 | Culture war* |
| S81 | "Woke" |
| S82 | "Conspiracy" |
| S83 | "Mainstream media" |
| S84 | "Antifa" |
| S85 | Antifacist* |
| S86 | "Social populism" |
| S87 | "Far-left" |
| S88 | "Farleft" |
| S89 | Anti-corporat* |
| S90 | Anticorporat* |
| S91 | Anti-left* |
| S92 | Antileft* |
| S93 | Anti-conservative* |
| S94 | Anticonservative* |
| S95 | Hostil* N2 left* |
| S96 | Hostil* N2 conservative* |
| S97 | S1 OR S2 OR S3 OR S4OR S5 OR S6 OR S7 ORS8 OR S9 OR S10 ORS11 OR S12 OR S13 ORS14 OR S15 OR S16 ORS17 OR S18 OR S19 ORS20 OR S21 OR S22 ORS23 OR S24 OR S25 ORS26 OR S27 OR S28 ORS29 OR S30 OR S31 ORS32 OR S33 OR S34 ORS35 OR S36 OR S37 ORS38 OR S39 OR S40 ORS41 OR S42 OR S43 ORS44 OR S45 OR S46 OR  S47 OR S48 OR S49 ORS50 OR S51 OR S52 ORS53 OR S54 OR S55 ORS56 OR S57 OR S58 ORS59 OR S60 OR S61 ORS62 OR S63 OR S64 ORS65 OR S66 OR S67 ORS68 OR S69 OR S70 ORS71 OR S72 OR S73 ORS74 OR S75 OR S76 ORS77 OR S78 OR S79 ORS80 OR S81 OR S82 ORS83 OR S84 OR S85 ORS86 OR S87 OR S88 ORS89 OR S90 OR S91 ORS92 OR S93 OR S94 ORS95 OR S96 |
| S98 | "Abortion" |
| S99 | "Sexual health" |
| S100 | "Reproductive health" |
| S101 | Pregnan* |
| S102 | Contracepti* |
| S103 | Covid* |
| S104 | Corona* |
| S105 | "SARS-CoV-2" |
| S106 | "Pandemic" |
| S107 | "Influenza" |
| S108 | "Flu" |
| S109 | "Facemask" |
| S110 | "Face-mask" |
| S111 | "Isolation" |
| S112 | Social distanc* |
| S113 | Non-pharmac* |
| S114 | Nonpharmac* |
| S115 | Infection control* |
| S116 | Vaccin* |
| S117 | Anti-vaccination Movement |
| S118 | Vaccination Refusal |
| S119 | Vaccination Hesitancy |
| S120 | Immunis* |
| S121 | Immuniz* |
| S122 | "MMR" |
| S123 | "Measles" |
| S124 | "Mumps" |
| S125 | "Rubella" |
| S126 | "Climate changeprevention" |
| S127 | "Environmentalprotection" |
| S128 | "Pollution" |
| S129 | "Emissions" |
| S130 | 15-minute cit* |
| S131 | fifteen-minute cit* |
| S132 | "road safety" |
| S133 | speed camera* |
| S134 | enforcement camera* |
| S135 | "mental health" |
| S136 | mental illness* |
| S137 | "anxiety" |
| S138 | depress* |
| S139 | physical* active* |
| S140 | physical* exercise* |
| S141 | "diet" |
| S142 | "nutrition" |
| S143 | obes* |
| S144 | over-weight* |
| S145 | overweight* |
| S146 | cigarette* |
| S147 | "tobacco" |
| S148 | "vaping" |
| S149 | vape* |
| S150 | smok* |
| S151 | "Illegal" N2 drug* |
| S152 | "Illicit" N2 drug* |
| S153 | "Recreational" N2 drug* |
| S154 | substance* |
| S155 | alcohol* |
| S156 | Screening* |
| S157 | "Gun control" |
| S158 | "Firearm ban" |
| S159 | "Gun-violence prevention" |
| S160 | "Gun safety" |
| S161 | "Gambling" |
| S162 | "Betting" |
| S163 | "Fluoridation" |
| S164 | Fluoridi#ation |
| S165 | "Health-care access" |
| S166 | "Healthcare access" |
| S167 | S98 OR S99 OR S100OR S101 OR S102 ORS103 OR S104 OR S105OR S106 OR S107 ORS108 OR S109 OR S110OR S111 OR S112 ORS113 OR S114 OR S115OR S116 OR S117 ORS118 OR S119 OR S120OR S121 OR S122 ORS123 OR S124 OR S125OR S126 OR S127 ORS128 OR S129 OR S130OR S131 OR S132 ORS133 OR S134 OR S135 OR S136 OR S137 ORS138 OR S139 OR S140OR S141 OR S142 ORS143 OR S144 OR S145OR S146 OR S147 ORS148 OR S149 OR S150OR S151 OR S152 ORS153 OR S154 OR S155OR S156 OR S157 ORS158 OR S159 OR S160OR S161 OR S162 ORS163 OR S164 OR S165OR S166 |
| S168 | Attitude* |
| S169 | Belief* |
| S170 | View* |
| S171 | Acceptab* |
| S172 | Adhere* |
| S173 | Effect* or "Effective" |
| S174 | Outcome* |
| S175 | Impact* |
| S176 | Benefit* |
| S177 | S168 OR S169 OR S170OR S171 OR S172 ORS173 OR S174 OR S175OR S176 |
| S178 | S97 AND S167 AND S177 |

## Global Index Medicus

| **Database name** | Global Index Medicus |
| --- | --- |
| **Database platform** | https://www.globalindexmedicus.net/ |
| **Dates of database coverage** | Complete database to search date |
| **Date searched** | 19 February 2024 |
| **Number of results** | 1,652 |

| **#** | **Query** |
| --- | --- |
| 1 | (tw:(Populis*)) OR (tw:(Illiberal*)) OR (tw:(Anti-liberal*)) OR (tw:(Antiliberal*)) OR (tw:(Anti-right*)) OR (tw:(Antiright*)) OR (tw:(Hostil* toward* the right*)) OR (tw:(Hostil* toward* liberal*)) OR (tw:(Anti-EDI*)) OR (tw:(Hostil* toward* EDI*)) OR (tw:(Anti-equalit*)) OR (tw:(Antiequalit*)) OR (tw:(Hostil* toward* equalit*)) OR (tw:(Anti-divers*)) OR (tw:(Antidivers*)) OR (tw:(Hostil* toward* divers*)) OR (tw:(Anti-inclu*)) OR (tw:(Anti-intervent*)) OR (tw:(Hostil* toward* intervent*)) OR (tw:(Anti-regulat*)) OR (tw:(Antiregulat*)) OR (tw:(Hostil* toward* regulat*)) OR (tw:(Hostil* toward* inclu*)) OR (tw:(“Racism”)) OR (tw:(Racis*)) OR (tw:(“Scapegoating”)) OR (tw:(“Xenophobia”)) OR (tw:(“Sexism”)) OR (tw:(Elitis*)) OR (tw:(“Sovereignty”)) OR (tw:(Nationalis*)) OR (tw:(Nativis*)) OR (tw:(Pro-nativis*)) OR (tw:(Pronativis*)) OR (tw:(Anti-immigra*)) OR (tw:(Hostil* toward* immigra*)) OR (tw:(Anti-migra*)) OR (tw:(Antimigra*)) OR (tw:(Hostil* toward* migra*)) OR (tw:(Anti-minorit*)) OR (tw:(Antiminorit*)) OR (tw:(Hostil* toward* minorit*)) OR (tw:(Anti-ethnic*)) OR (tw:(Antiethnic*)) OR (tw:(Hostil* toward* ethnic*)) OR (tw:(Hostil* toward* raci*)) OR (tw:(“Homophobia”)) OR (tw:(Homophobi*)) OR (tw:(Transphobi*)) OR (tw:(Anti-globali*)) OR (tw:(Antiglobali*)) OR (tw:(Hostil* toward* globali*)) OR (tw:(Hostil* toward* the wealth*)) OR (tw:(“Anti-cosmopolitan”)) OR (tw:(“Anticosmopolitan”)) OR (tw:(“Far-right”)) OR (tw:(“Anti-establishment”)) OR (tw:(“Antiestablishment”)) OR (tw:(Hostil* toward* the establishment*)) OR (tw:(Trust* in the establishment*)) OR (tw:(“Brexit”)) OR (tw:(Anti-expert*)) OR (tw:(Antiexpert*)) OR (tw:(Hostil* toward* expert*)) OR (tw:(Trust* in expert*)) OR (tw:(Anti-scien*)) OR (tw:(Antiscien*)) OR (tw:(Hostil* toward* scien*)) OR (tw:(Trust* in scient*)) OR (tw:(Hostil* toward* research*)) OR (tw:(Trust* in research*)) OR (tw:(Anti-business*)) OR (tw:(Antibusiness*)) OR (tw:(Hostil* toward* business)) OR (tw:(Trust* in business)) OR (tw:(“Anti-government”)) OR (tw:(“Antigovernment”)) OR (tw:(Hostil* toward* government)) OR (tw:(Trust* in government)) OR (tw:(Culture war*)) OR (tw:(“Woke”)) OR (tw:(“Conspiracy”)) OR (tw:(“Mainstream media”)) OR (tw:(“Antifa”)) OR (tw:(Antifacist*)) OR (tw:(“Social populism”)) OR (tw:(“Far-left”)) OR (tw:(“Farleft”)) OR (tw:(Anti-corporat*)) OR (tw:(Anticorporat*)) OR (tw:(Anti-left*)) OR (tw:(Antileft*)) OR (tw:(Anti-conservative*)) OR (tw:(Anticonservative*)) OR (tw:(Hostil* toward* the left*)) OR (tw:(Hostil* toward* conservative*)) |
| 2 | (tw:(“Abortion”)) OR (tw:(“Sexual health”)) OR (tw:(“Reproductive health”)) OR (tw:(Pregnan*)) OR (tw:(Contracepti*)) OR (tw:(Covid*)) OR (tw:(Corona*)) OR (tw:(“SARS-CoV-2”)) OR (tw:(“Pandemic”)) OR (tw:(“Influenza”)) OR (tw:(“Flu”)) OR (tw:(“Facemask”)) OR (tw:(“Face-mask”)) OR (tw:(“Isolation”)) OR (tw:(Social distanc*)) OR (tw:(Non-pharmac*)) OR (tw:(Nonpharmac*)) OR (tw:(Infection control*)) OR (tw:(Vaccin*)) OR (tw:(“Anti-Vaccination Movement”)) OR (tw:(“Vaccination Refusal”)) OR (tw:(Immunis*)) OR (tw:(Immuniz*)) OR (tw:(“MMR”)) OR (tw:(“Measles”)) OR (tw:(“Mumps”)) OR (tw:(“Rubella”)) OR (tw:(“Climate change prevention”)) OR (tw:(“Environmental protection”)) OR (tw:(“Pollution”)) OR (tw:(“Emissions”)) OR (tw:(15-minute cit*)) OR (tw:(fifteen-minute cit*)) OR (tw:(“road safety”)) OR (tw:(speed camera*)) OR (tw:(enforcement camera*)) OR (tw:(“mental health”)) OR (tw:(mental illness*)) OR (tw:(“anxiety”)) OR (tw:(depress*)) OR (tw:(physical* active*)) OR (tw:(physical* exercise*)) OR (tw:(“diet”)) OR (tw:(“nutrition”)) OR (tw:(obes*)) OR (tw:(over-weight*)) OR (tw:(overweight*)) OR (tw:(cigarette*)) OR (tw:(“tobacco”)) OR (tw:(“vaping”)) OR (tw:(vape*)) OR (tw:(smok*)) OR (tw:(Illegal drug*)) OR (tw:(Illicit drug*)) OR (tw:(Recreational drug*)) OR (tw:(substance*)) OR (tw:(alcohol*)) OR (tw:(Screening*)) OR (tw:(“Gun control”)) OR (tw:(“Firearm ban”)) OR (tw:(“Gun-violence prevention”)) OR (tw:(“Gun safety”)) OR (tw:(“Gambling”)) OR (tw:(“Betting”)) OR (tw:(“Fluoridation”)) OR (tw:(Fluoridi#ation)) OR (tw:(“Health-care access”)) OR (tw:(“Healthcare access”)) |
| 3 | (tw:(Attitude*)) OR (tw:(Belief*)) OR (tw:(View*)) OR (tw:(Acceptab*)) OR (tw:(Adhere*)) OR (tw:(Effect*)) OR (tw:(Effective*)) OR (tw:(Outcome*)) OR (tw:(Impact*)) OR (tw:(Benefit*)) |
| 4 | 1 and 2 and 3 |

## ProQuest

| **Database name** | ProQuest (International Bibliography of the Social Sciences and Dissertation Abstracts) |
| --- | --- |
| **Database platform** | https://www.proquest.com/ |
| **Dates of database coverage** | Complete database to search date |
| **Date searched** | 19 February 2024 |
| **Number of results** | 2,463 |

| **#** | **Query** |
| --- | --- |
| 1 | (title(Populis* OR Illiberal* OR Anti-liberal* OR Antiliberal* OR Anti-right* OR Antiright* OR Hostil* NEAR/2 right* OR Hostil* NEAR/2 liberal* OR Anti-EDI* OR Hostil* NEAR/2 EDI* OR Anti-equalit* OR Antiequalit* OR Hostil* NEAR/2 equalit* OR Anti-divers* OR Antidivers* OR Hostil* NEAR/2 divers* OR Anti-inclu* OR Anti-intervent* OR Hostil* NEAR/2 intervent* OR Anti-regulat* OR Antiregulat* OR Hostil* NEAR/2 regulat* OR Hostil* NEAR/2 inclu* OR "Racism" OR Racis* OR "Scapegoating" OR "Xenophobia" OR "Sexism" OR Elitis* OR "Sovereignty" OR Nationalis* OR Nativis* OR Pro-nativis* OR Pronativis* OR Anti-immigra* OR Hostil* NEAR/2 immigra* OR Anti-migra* OR Antimigra* OR Hostil* NEAR/2 migra* OR Anti-minorit* OR Antiminorit* OR Hostil* NEAR/2 minorit* OR Anti-ethnic* OR Antiethnic* OR Hostil* NEAR/2 ethnic* OR Hostil* NEAR/2 raci* OR "Homophobia" OR Homophobi* OR Transphobi* OR Anti-globali* OR Antiglobali* OR Hostil* NEAR/2 globali* OR Hostil* NEAR/2 wealth* OR "Anti-cosmopolitan" OR "Anticosmopolitan" OR "Far-right" OR "Anti-establishment" OR "Antiestablishment" OR Hostil* NEAR/2 establishment* OR Trust* NEAR/2 establishment* OR "Brexit" OR Anti-expert* OR Antiexpert* OR Hostil* NEAR/2 expert* OR Trust* NEAR/2 expert* OR Anti-scien* OR Antiscien* OR Hostil* NEAR/2 scien* OR Trust* NEAR/2 scient* OR Hostil* NEAR/2 research* OR Trust* NEAR/2 research* OR Anti-business* OR Antibusiness* OR Hostil* NEAR/2 "business" OR Trust* NEAR/2 "business" OR "Anti-government" OR "Antigovernment" OR Hostil* NEAR/2 "government" OR Trust* NEAR/2 "government" OR Culture war* OR "Woke" OR "Conspiracy" OR "Mainstream media" OR "Antifa" OR Antifacist* OR "Social populism" OR "Far-left" OR "Farleft" OR Anti-corporat* OR Anticorporat* OR Anti-left* OR Antileft* OR Anti-conservative* OR Anticonservative* OR Hostil* NEAR/2 left* OR Hostil* NEAR/2 conservative*) OR abstract(Populis* OR Illiberal* OR Anti-liberal* OR Antiliberal* OR Anti-right* OR Antiright* OR Hostil* NEAR/2 right* OR Hostil* NEAR/2 liberal* OR Anti-EDI* OR Hostil* NEAR/2 EDI* OR Anti-equalit* OR Antiequalit* OR Hostil* NEAR/2 equalit* OR Anti-divers* OR Antidivers* OR Hostil* NEAR/2 divers* OR Anti-inclu* OR Anti-intervent* OR Hostil* NEAR/2 intervent* OR Anti-regulat* OR Antiregulat* OR Hostil* NEAR/2 regulat* OR Hostil* NEAR/2 inclu* OR "Racism" OR Racis* OR "Scapegoating" OR "Xenophobia" OR "Sexism" OR Elitis* OR "Sovereignty" OR Nationalis* OR Nativis* OR Pro-nativis* OR Pronativis* OR Anti-immigra* OR Hostil* NEAR/2 immigra* OR Anti-migra* OR Antimigra* OR Hostil* NEAR/2 migra* OR Anti-minorit* OR Antiminorit* OR Hostil* NEAR/2 minorit* OR Anti-ethnic* OR Antiethnic* OR Hostil* NEAR/2 ethnic* OR Hostil* NEAR/2 raci* OR "Homophobia" OR Homophobi* OR Transphobi* OR Anti-globali* OR Antiglobali* OR Hostil* NEAR/2 globali* OR Hostil* NEAR/2 wealth* OR "Anti-cosmopolitan" OR "Anticosmopolitan" OR "Far-right" OR "Anti-establishment" OR "Antiestablishment" OR Hostil* NEAR/2 establishment* OR Trust* NEAR/2 establishment* OR "Brexit" OR Anti-expert* OR Antiexpert* OR Hostil* NEAR/2 expert* OR Trust* NEAR/2 expert* OR Anti-scien* OR Antiscien* OR Hostil* NEAR/2 scien* OR Trust* NEAR/2 scient* OR Hostil* NEAR/2 research* OR Trust* NEAR/2 research* OR Anti-business* OR Antibusiness* OR Hostil* NEAR/2 "business" OR Trust* NEAR/2 "business" OR "Anti-government" OR "Antigovernment" OR Hostil* NEAR/2 "government" OR Trust* NEAR/2 "government" OR Culture war* OR "Woke" OR "Conspiracy" OR "Mainstream media" OR "Antifa" OR Antifacist* OR "Social populism" OR "Far-left" OR "Farleft" OR Anti-corporat* OR Anticorporat* OR Anti-left* OR Antileft* OR Anti-conservative* OR Anticonservative* OR Hostil* NEAR/2 left* OR Hostil* NEAR/2 conservative*)) AND (title("Abortion" OR "Sexual health" OR "Reproductive health" OR Pregnan* OR Contracepti* OR Covid* OR Corona* OR "SARS-CoV-2" OR "Pandemic" OR "Influenza" OR "Flu" OR "Facemask" OR "Face-mask" OR "Isolation" OR Social distanc* OR Non-pharmac* OR Nonpharmac* OR Infection control* OR Vaccin* OR "Anti-Vaccination Movement" OR "Vaccination Refusal" OR "Vaccination Hesitancy" OR Immunis* OR Immuniz* OR "MMR" OR "Measles" OR "Mumps" OR "Rubella" OR "Climate change prevention" OR "Environmental protection" OR "Pollution" OR "Emissions" OR 15-minute cit* OR fifteen-minute cit* OR "road safety" OR speed camera* OR enforcement camera* OR "mental health" OR mental illness* OR "anxiety" OR depress* OR physical* active* OR physical* exercise* OR "diet" OR "nutrition" OR obes* OR over-weight* OR overweight* OR cigarette* OR "tobacco" OR "vaping" OR vape* OR smok* OR "Illegal" NEAR/2 drug* OR "Illicit" NEAR/2 drug* OR "Recreational" NEAR/2 drug* OR substance* OR alcohol* OR Screening* OR "Gun control" OR "Firearm ban" OR "Gun-violence prevention" OR "Gun safety" OR "Gambling" OR "Betting" OR "Fluoridation" OR Fluoridi?ation OR "Health-care access" OR "Healthcare access") OR abstract("Abortion" OR "Sexual health" OR "Reproductive health" OR Pregnan* OR Contracepti* OR Covid* OR Corona* OR "SARS-CoV-2" OR "Pandemic" OR "Influenza" OR "Flu" OR "Facemask" OR "Face-mask" OR "Isolation" OR Social distanc* OR Non-pharmac* OR Nonpharmac* OR Infection control* OR Vaccin* OR "Anti-Vaccination Movement" OR "Vaccination Refusal" OR "Vaccination Hesitancy" OR Immunis* OR Immuniz* OR "MMR" OR "Measles" OR "Mumps" OR "Rubella" OR "Climate change prevention" OR "Environmental protection" OR "Pollution" OR "Emissions" OR 15-minute cit* OR fifteen-minute cit* OR "road safety" OR speed camera* OR enforcement camera* OR "mental health" OR mental illness* OR "anxiety" OR depress* OR physical* active* OR physical* exercise* OR "diet" OR "nutrition" OR obes* OR over-weight* OR overweight* OR cigarette* OR "tobacco" OR "vaping" OR vape* OR smok* OR "Illegal" NEAR/2 drug* OR "Illicit" NEAR/2 drug* OR "Recreational" NEAR/2 drug* OR substance* OR alcohol* OR Screening* OR "Gun control" OR "Firearm ban" OR "Gun-violence prevention" OR "Gun safety" OR "Gambling" OR "Betting" OR "Fluoridation" OR Fluoridi?ation OR "Health-care access" OR "Healthcare access")) AND (title(Attitude* OR Belief* OR View* OR Acceptab* OR Adhere* OR Effect* OR Effective* OR Outcome* OR Impact* OR Benefit*) OR abstract(Attitude* OR Belief* OR View* OR Acceptab* OR Adhere* OR Effect* OR Effective* OR Outcome* OR Impact* OR Benefit*)) AND pd(20080101-20241231) |

## Ovid MEDLINE

| **Database name** | Ovid MEDLINE (includes Econlit, Embase, Global Health, Ovid MEDLINE (ALL), PsycINFO, Social Policy and Practice) |
| --- | --- |
| **Database platform** | https://www.wolterskluwer.com/en/solutions/ovid/ovid-medline-901 |
| **Dates of database coverage** | 1950-present |
| **Date searched** | 19 February 2024 |
| **Number of results** | 20,776 |

| **#** | **Query** | **Results from 19 February 2024** |
| --- | --- | --- |
| 1 | Populis*.ti,ab. | 3,760 |
| 2 | Illiberal*.ti,ab. | 378 |
| 3 | Anti-liberal*.ti,ab. | 36 |
| 4 | Antiliberal*.ti,ab. | 23 |
| 5 | Anti-right*.ti,ab. | 30 |
| 6 | Antiright*.ti,ab. | 1 |
| 7 | (Hostil* adj2 right*).ti,ab. | 15 |
| 8 | (Hostil* adj2 liberal*).ti,ab. | 6 |
| 9 | Anti-EDI*.ti,ab. | 70 |
| 10 | (Hostil* adj2 EDI*).ti,ab. | 7 |
| 11 | Anti-equalit*.ti,ab. | 14 |
| 12 | Antiequalit*.ti,ab. | 0 |
| 13 | (Hostil* adj2 equalit*).ti,ab. | 7 |
| 14 | Anti-divers*.ti,ab. | 12 |
| 15 | Antidivers*.ti,ab. | 3 |
| 16 | (Hostil* adj2 divers*).ti,ab. | 65 |
| 17 | Anti-inclu*.ti,ab. | 19 |
| 18 | Anti-intervent*.ti,ab. | 20 |
| 19 | (Hostil* adj2 intervent*).ti,ab. | 126 |
| 20 | Anti-regulat*.ti,ab. | 110 |
| 21 | Antiregulat*.ti,ab. | 41 |
| 22 | (Hostil* adj2 regulat*).ti,ab. | 83 |
| 23 | (Hostil* adj2 inclu*).ti,ab. | 653 |
| 24 | Racism/ | 32,224 |
| 25 | Racis*.ti,ab. | 42,933 |
| 26 | Scapegoating/ | 282 |
| 27 | Xenophobia/ | 1,384 |
| 28 | Sexism/ | 11,631 |
| 29 | Elitis*.ti,ab. | 1,725 |
| 30 | Sovereignty.ti,ab. | 6,425 |
| 31 | Nationalis*.ti,ab. | 9,001 |
| 32 | Nativis*.ti,ab. | 1,327 |
| 33 | Pro-nativis*.ti,ab. | 1 |
| 34 | Pronativis*.ti,ab. | 0 |
| 35 | Anti-immigra*.ti,ab. | 1,636 |
| 36 | (Hostil* adj2 immigra*).ti,ab. | 128 |
| 37 | Anti-migra*.ti,ab. | 5,022 |
| 38 | Antimigra*.ti,ab. | 4,184 |
| 39 | (Hostil* adj2 migra*).ti,ab. | 41 |
| 40 | Anti-minorit*.ti,ab. | 61 |
| 41 | Antiminorit*.ti,ab. | 22 |
| 42 | (Hostil* adj2 minorit*).ti,ab. | 33 |
| 43 | Anti-ethnic*.ti,ab. | 10 |
| 44 | Antiethnic*.ti,ab. | 1 |
| 45 | (Hostil* adj2 ethnic*).ti,ab. | 94 |
| 46 | (Hostil* adj2 raci*).ti,ab. | 228 |
| 47 | Homophobia/ | 7,550 |
| 48 | Homophobi*.ti,ab. | 9,221 |
| 49 | Transphobi*.ti,ab. | 1,707 |
| 50 | Anti-globali*.ti,ab. | 203 |
| 51 | Antiglobali*.ti,ab. | 69 |
| 52 | (Hostil* adj2 globali*).ti,ab. | 1 |
| 53 | (Hostil* adj2 wealth*).ti,ab. | 1 |
| 54 | Anti-cosmopolitan.ti,ab. | 6 |
| 55 | Anticosmopolitan.ti,ab. | 0 |
| 56 | Far-right.ti,ab. | 977 |
| 57 | Anti-establishment.ti,ab. | 161 |
| 58 | Antiestablishment.ti,ab. | 61 |
| 59 | (Hostil* adj2 establishment*).ti,ab. | 2 |
| 60 | (Trust* adj2 establishment*).ti,ab. | 326 |
| 61 | Brexit.ti,ab. | 3,836 |
| 62 | Anti-expert*.ti,ab. | 27 |
| 63 | Antiexpert*.ti,ab. | 0 |
| 64 | (Hostil* adj2 expert*).ti,ab. | 4 |
| 65 | (Trust* adj2 expert*).ti,ab. | 1,133 |
| 66 | Anti-scien*.ti,ab. | 378 |
| 67 | Antiscien*.ti,ab. | 262 |
| 68 | (Hostil* adj2 scien*).ti,ab. | 40 |
| 69 | (Trust* adj2 scient*).ti,ab. | 756 |
| 70 | (Hostil* adj2 research*).ti,ab. | 144 |
| 71 | (Trust* adj2 research*).ti,ab. | 3,659 |
| 72 | Anti-business*.ti,ab. | 24 |
| 73 | Antibusiness*.ti,ab. | 17 |
| 74 | (Hostil* adj2 business).ti,ab. | 23 |
| 75 | (Trust* adj2 business).ti,ab. | 313 |
| 76 | Anti-government.ti,ab. | 174 |
| 77 | Antigovernment.ti,ab. | 63 |
| 78 | (Hostil* adj2 government).ti,ab. | 30 |
| 79 | (Trust* adj2 government).ti,ab. | 2,391 |
| 80 | Culture war*.ti,ab. | 614 |
| 81 | Woke.ti,ab. | 2,866 |
| 82 | Conspiracy.ti,ab. | 5,745 |
| 83 | Mainstream media.ti,ab. | 1,957 |
| 84 | Antifa.ti,ab. | 3 |
| 85 | Antifacist*.ti,ab. | 0 |
| 86 | Social populism.ti,ab. | 0 |
| 87 | Far-left.ti,ab. | 332 |
| 88 | Farleft.ti,ab. | 0 |
| 89 | Anti-corporat*.ti,ab. | 57 |
| 90 | Anticorporat*.ti,ab. | 26 |
| 91 | Anti-left*.ti,ab. | 23 |
| 92 | Antileft*.ti,ab. | 1 |
| 93 | Anti-conservative*.ti,ab. | 189 |
| 94 | Anticonservative*.ti,ab. | 139 |
| 95 | (Hostil* adj2 left*).ti,ab. | 9 |
| 96 | (Hostil* adj2 conservative*).ti,ab. | 10 |
| 97 | or/1-96 [POPULIST ATTITUDES] | 139,662 |
| 98 | Abortion.ti,ab. | 139,846 |
| 99 | Sexual health.ti,ab. | 48,617 |
| 100 | Reproductive health.ti,ab. | 60,012 |
| 101 | Pregnan*.ti,ab. | 1,600,411 |
| 102 | Contracepti*.ti,ab. | 196,376 |
| 103 | Covid*.ti,ab. | 947,013 |
| 104 | Corona*.ti,ab. | 1,623,075 |
| 105 | SARS-CoV-2.ti,ab. | 306,164 |
| 106 | Pandemic.ti,ab. | 604,598 |
| 107 | Influenza.ti,ab. | 306,224 |
| 108 | Flu.ti,ab. | 51,962 |
| 109 | Facemask.ti,ab. | 4,578 |
| 110 | Face-mask.ti,ab. | 11,509 |
| 111 | Isolation.ti,ab. | 783,532 |
| 112 | Social distanc*.ti,ab. | 34,998 |
| 113 | Non-pharmac*.ti,ab. | 53,562 |
| 114 | Nonpharmac*.ti,ab. | 31,146 |
| 115 | Infection control*.ti,ab. | 76,614 |
| 116 | Vaccin*.ti,ab. | 1,134,105 |
| 117 | Anti-Vaccination Movement/ | 843 |
| 118 | Vaccination Refusal/ | 3,624 |
| 119 | Vaccination Hesitancy/ | 7,529 |
| 120 | Immunis*.ti,ab. | 38,577 |
| 121 | Immuniz*.ti,ab. | 391,916 |
| 122 | MMR.ti,ab. | 32,803 |
| 123 | Measles.ti,ab. | 70,542 |
| 124 | Mumps.ti,ab. | 24,999 |
| 125 | Rubella.ti,ab. | 36,467 |
| 126 | Climate change prevention.ti,ab. | 29 |
| 127 | Environmental protection.ti,ab. | 43,669 |
| 128 | Pollution.ti,ab. | 339,909 |
| 129 | Emissions.ti,ab. | 193,137 |
| 130 | 15-minute cit*.ti,ab. | 30 |
| 131 | fifteen-minute cit*.ti,ab. | 2 |
| 132 | road safety.ti,ab. | 10,039 |
| 133 | speed camera*.ti,ab. | 4,144 |
| 134 | enforcement camera*.ti,ab. | 71 |
| 135 | mental health.ti,ab. | 810,626 |
| 136 | mental illness*.ti,ab. | 153,894 |
| 137 | anxiety.ti,ab. | 926,472 |
| 138 | depress*.ti,ab. | 1,804,599 |
| 139 | physical* active*.ti,ab. | 36,559 |
| 140 | physical* exercise*.ti,ab. | 61,989 |
| 141 | diet.ti,ab. | 1,257,289 |
| 142 | nutrition.ti,ab. | 648,759 |
| 143 | obes*.ti,ab. | 1,186,981 |
| 144 | over-weight*.ti,ab. | 2,782 |
| 145 | overweight*.ti,ab. | 300,837 |
| 146 | cigarette*.ti,ab. | 247,693 |
| 147 | tobacco.ti,ab. | 333,812 |
| 148 | vaping.ti,ab. | 9,777 |
| 149 | vape*.ti,ab. | 4,754 |
| 150 | smok*.ti,ab. | 1,031,728 |
| 151 | (Illegal adj2 drug*).ti,ab. | 10,063 |
| 152 | (Illicit adj2 drug*).ti,ab. | 46,177 |
| 153 | (Recreational adj2 drug*).ti,ab. | 9,961 |
| 154 | substance*.ti,ab. | 1,049,837 |
| 155 | alcohol*.ti,ab. | 1,233,195 |
| 156 | Screening*.ti,ab. | 1,933,521 |
| 157 | Gun control.ti,ab. | 1,154 |
| 158 | Firearm ban.ti,ab. | 0 |
| 159 | Gun-violence prevention.ti,ab. | 231 |
| 160 | Gun safety.ti,ab. | 403 |
| 161 | Gambling.ti,ab. | 36,229 |
| 162 | Betting.ti,ab. | 3,786 |
| 163 | Fluoridation.ti,ab. | 7,837 |
| 164 | Fluoridi#ation.ti,ab. | 23 |
| 165 | Health-care access.ti,ab. | 11,361 |
| 166 | Healthcare access.ti,ab. | 9,755 |
| 167 | or/98-166 [PUBLIC HEALTH INTERVENTIONS] | 15,938,673 |
| 168 | Attitude*.ti,ab. | 764,403 |
| 169 | Belief*.ti,ab. | 436,984 |
| 170 | View*.ti,ab. | 1,812,063 |
| 171 | Acceptab*.ti,ab. | 681,917 |
| 172 | Adhere*.ti,ab. | 695,082 |
| 173 | Effect*.ti,ab. | 22,972,732 |
| 174 | Effective*.ti,ab. | 7,229,965 |
| 175 | Outcome*.ti,ab. | 6,981,530 |
| 176 | Impact*.ti,ab. | 5,100,904 |
| 177 | Benefit*.ti,ab. | 2,746,378 |
| 178 | or/168-177 [OUTCOME] | 33,192,990 |
| 179 | 97 and 167 and 178 | 23,208 |
| 180 | limit 179 to yr="2008-Current" | 20,776 |

## Scopus

| **Database name** | Scopus |
| --- | --- |
| **Database platform** | www.scopus.com |
| **Dates of database coverage** | 1970-present |
| **Date searched** | 19 February 2024 |
| **Number of results** | 15,717 |

| **#** | **Query** |
| --- | --- |
| 1 | ( ( TITLE-ABS-KEY ( populis* ) OR TITLE-ABS-KEY ( illiberal* ) OR TITLE-ABS-KEY ( anti-liberal* ) OR TITLE-ABS-KEY ( antiliberal* ) OR TITLE-ABS-KEY ( anti-right* ) OR TITLE-ABS-KEY ( antiright* ) OR TITLE-ABS-KEY ( ( "hostil* PRE/2 right*" ) ) OR TITLE-ABS-KEY ( ( "hostil* PRE/2 liberal*" ) ) OR TITLE-ABS-KEY ( anti-edi* ) OR TITLE-ABS-KEY ( ( "hostil* PRE/2 edi*") ) OR TITLE-ABS-KEY ( anti-equalit* ) OR TITLE-ABS-KEY ( antiequalit* ) OR TITLE-ABS-KEY ( ( "hostil* PRE/2 equalit*") ) OR TITLE-ABS-KEY ( anti-divers* ) OR TITLE-ABS-KEY ( antidivers* ) OR TITLE-ABS-KEY ( ( "hostil* PRE/2 divers*") ) OR TITLE-ABS-KEY ( anti-inclu* ) OR TITLE-ABS-KEY ( anti-intervent* ) OR TITLE-ABS-KEY ( ( "hostil* PRE/2 intervent*" ) ) OR TITLE-ABS-KEY ( anti-regulat* ) OR TITLE-ABS-KEY ( antiregulat* ) OR TITLE-ABS-KEY ( ( "hostil* PRE/2 regulat*" ) ) OR TITLE-ABS-KEY ( ( "hostil* PRE/2 inclu*" ) ) OR TITLE-ABS-KEY ( "racism" ) OR TITLE-ABS-KEY ( racis* ) OR TITLE-ABS-KEY ( "scapegoating") OR TITLE-ABS-KEY ( "xenophobia") OR TITLE-ABS-KEY ( "sexism" ) OR TITLE-ABS-KEY ( elitis* ) OR TITLE-ABS-KEY ( "sovereignty" ) OR TITLE-ABS-KEY ( nationalis* ) OR TITLE-ABS-KEY ( nativis* ) OR TITLE-ABS-KEY ( pro-nativis* ) OR TITLE-ABS-KEY ( pronativis* ) OR TITLE-ABS-KEY ( anti-immigra* ) OR TITLE-ABS-KEY ( ("hostil* PRE/2 immigra*") ) OR TITLE-ABS-KEY ( anti-migra* ) OR TITLE-ABS-KEY ( antimigra* ) OR TITLE-ABS-KEY ( ( "hostil* PRE/2 migra*" ) ) OR TITLE-ABS-KEY ( anti-minorit* ) OR TITLE-ABS-KEY ( antiminorit* ) OR TITLE-ABS-KEY ( ( "hostil* PRE/2 minorit*" ) ) OR TITLE-ABS-KEY ( anti-ethnic* ) OR TITLE-ABS-KEY ( antiethnic* ) OR TITLE-ABS-KEY ( ( "hostil* PRE/2 ethnic*" ) ) OR TITLE-ABS-KEY ( ( "hostil* PRE/2 raci*" ) ) OR TITLE-ABS-KEY ( "homophobia" ) OR TITLE-ABS-KEY ( homophobi* ) OR TITLE-ABS-KEY ( transphobi* ) OR TITLE-ABS-KEY ( anti-globali* ) OR TITLE-ABS-KEY ( antiglobali* ) OR TITLE-ABS-KEY ( ( "hostil* PRE/2 globali*" ) ) OR TITLE-ABS-KEY ( ( "hostil* PRE/2 wealth*" ) ) OR TITLE-ABS-KEY ( "anti-cosmopolitan") OR TITLE-ABS-KEY ( "anticosmopolitan" ) OR TITLE-ABS-KEY ("far-right") OR TITLE-ABS-KEY ("anti-establishment") OR TITLE-ABS-KEY ( "antiestablishment") OR TITLE-ABS-KEY ( ( "hostil* PRE/2 establishment*") ) OR TITLE-ABS-KEY ( ( "trust* PRE/2 establishment*") ) OR TITLE-ABS-KEY ("Brexit") OR TITLE-ABS-KEY ( anti-expert* ) OR TITLE-ABS-KEY ( antiexpert* ) OR TITLE-ABS-KEY ( ("hostil* PRE/2 expert*" ) ) OR TITLE-ABS-KEY ( ( "trust* PRE/2 expert*") ) OR TITLE-ABS-KEY ( anti-scien* ) OR TITLE-ABS-KEY ( antiscien* ) OR TITLE-ABS-KEY ( ( "trust* PRE/2 scient*") ) OR TITLE-ABS-KEY ( ( "hostil* PRE/2 research*") ) OR TITLE-ABS-KEY ( ( "trust* PRE/2 research*") ) OR TITLE-ABS-KEY ( anti-business* ) OR TITLE-ABS-KEY ( antibusiness* ) OR TITLE-ABS-KEY ( ( "hostil* PRE/2 business" ) ) OR TITLE-ABS-KEY ( ( "trust* PRE/2 business") ) OR TITLE-ABS-KEY ( "anti-government" ) OR TITLE-ABS-KEY ( "antigovernment") OR TITLE-ABS-KEY ( ( "hostil* PRE/2 government") ) OR TITLE-ABS-KEY ( ( "trust* PRE/2 government") ) OR TITLE-ABS-KEY ( "culture war*") OR TITLE-ABS-KEY ( "woke" ) OR TITLE-ABS-KEY ( "conspiracy" ) OR TITLE-ABS-KEY ( "mainstream media" ) OR TITLE-ABS-KEY ( "antifa" ) OR TITLE-ABS-KEY ( antifacist* ) OR TITLE-ABS-KEY ( "social populism") OR TITLE-ABS-KEY ( "far-left") OR TITLE-ABS-KEY ( "farleft" ) OR TITLE-ABS-KEY ( anti-corporat* ) OR TITLE-ABS-KEY ( anticorporat* ) OR TITLE-ABS-KEY ( anti-left* ) OR TITLE-ABS-KEY ( antileft* ) OR TITLE-ABS-KEY ( anti-conservative* ) OR TITLE-ABS-KEY ( anticonservative* ) OR TITLE-ABS-KEY ( ("hostil* PRE/2 left*") ) OR TITLE-ABS-KEY ( ( "hostil* PRE/2 conservative*") ) ) ) AND ( ( TITLE-ABS-KEY ( "abortion" ) OR TITLE-ABS-KEY ( "sexual health" ) OR TITLE-ABS-KEY ( "reproductive health" ) OR TITLE-ABS-KEY ( pregnan* ) OR TITLE-ABS-KEY ( contracepti* ) OR TITLE-ABS-KEY ( covid* ) OR TITLE-ABS-KEY ( corona* ) OR TITLE-ABS-KEY ( "sars-cov-2" ) OR TITLE-ABS-KEY ( "pandemic" ) OR TITLE-ABS-KEY ( "influenza" ) OR TITLE-ABS-KEY ( "flu" ) OR TITLE-ABS-KEY ( "facemask" ) OR TITLE-ABS-KEY ( "face-mask" ) OR TITLE-ABS-KEY ( "isolation" ) OR TITLE-ABS-KEY ("social distanc*") OR TITLE-ABS-KEY ( non-pharmac* ) OR TITLE-ABS-KEY ( nonpharmac* ) OR TITLE-ABS-KEY ( "infection control*" ) OR TITLE-ABS-KEY ( vaccin* ) OR TITLE-ABS-KEY ( "anti-vaccination movement" ) OR TITLE-ABS-KEY ( "vaccination refusal") OR TITLE-ABS-KEY ( immunis* ) OR TITLE-ABS-KEY ( immuniz* ) OR TITLE-ABS-KEY ( "mmr") OR TITLE-ABS-KEY ( "measles" ) OR TITLE-ABS-KEY ( "mumps" ) OR TITLE-ABS-KEY ( "rubella") OR TITLE-ABS-KEY ( "climate change prevention" ) OR TITLE-ABS-KEY ( "environmental protection" ) OR TITLE-ABS-KEY ( "pollution") OR TITLE-ABS-KEY ( "emissions" ) OR TITLE-ABS-KEY ( "15-minute cit*" ) OR TITLE-ABS-KEY ("fifteen-minute cit*" ) OR TITLE-ABS-KEY ( "road safety" ) OR TITLE-ABS-KEY ( "speed camera*" ) OR TITLE-ABS-KEY ( "enforcement camera*" ) OR TITLE-ABS-KEY ( "mental health" ) OR TITLE-ABS-KEY ("mental illness*") OR TITLE-ABS-KEY ( "anxiety" ) OR TITLE-ABS-KEY ( depress* ) OR TITLE-ABS-KEY ( "physical* active*" ) OR TITLE-ABS-KEY ( "physical* exercise*") OR TITLE-ABS-KEY ( "diet" ) OR TITLE-ABS-KEY ("nutrition") OR TITLE-ABS-KEY ( obes* ) OR TITLE-ABS-KEY ( over-weight* ) OR TITLE-ABS-KEY ( overweight* ) OR TITLE-ABS-KEY ( cigarette* ) OR TITLE-ABS-KEY ( "tobacco") OR TITLE-ABS-KEY ( "vaping") OR TITLE-ABS-KEY ( vape* ) OR TITLE-ABS-KEY ( smok* ) OR TITLE-ABS-KEY ( ( "illegal PRE/2 drug*") ) OR TITLE-ABS-KEY ( ("illicit PRE/2 drug*") ) OR TITLE-ABS-KEY ( ("recreational PRE/2 drug*") ) OR TITLE-ABS-KEY ( substance* ) OR TITLE-ABS-KEY ( alcohol* ) OR TITLE-ABS-KEY ( screening* ) OR TITLE-ABS-KEY ( "gun control") OR TITLE-ABS-KEY ( "firearm ban") OR TITLE-ABS-KEY ( "gun-violence prevention") OR TITLE-ABS-KEY ( "gun safety") OR TITLE-ABS-KEY ( "gambling" ) OR TITLE-ABS-KEY ( "betting") OR TITLE-ABS-KEY ("fluoridation") OR TITLE-ABS-KEY ( fluoridi#ation ) OR TITLE-ABS-KEY ( "health-care access") OR TITLE-ABS-KEY ("healthcare access" ) ) ) AND ( ( TITLE-ABS-KEY ( attitude* ) OR TITLE-ABS-KEY ( belief* ) OR TITLE-ABS-KEY ( view* ) OR TITLE-ABS-KEY ( acceptab* ) OR TITLE-ABS-KEY ( adhere* ) OR TITLE-ABS-KEY ( effect* ) OR TITLE-ABS-KEY ( effect* ) OR TITLE-ABS-KEY ( outcome* ) OR TITLE-ABS-KEY ( impact* ) OR TITLE-ABS-KEY ( benefit* ) ) ) AND PUBYEAR > 2007 AND PUBYEAR < 2025 |

## Sociological Research Online

| **Database name** | Sociological Research Online |
| --- | --- |
| **Database platform** | https://journals.sagepub.com/home/sro |
| **Dates of database coverage** | 1996-present |
| **Date searched** | 19 February 2024 |
| **Number of results** | 286 |

| **#** | **Query** |
| --- | --- |
| 1 | Title, Abstract and Keyword Search: “Populis*” OR “Illiberal*” OR “Anti-liberal*” OR “Antiliberal*” OR “Anti-right*” OR “Antiright*” OR “Hostil* toward* the right*” OR “Hostil* toward* liberal*” OR “Anti-EDI*” OR “Hostil* toward* EDI*” OR “Anti-equalit*” OR “Antiequalit*” OR “Hostil* toward* equalit*” OR “Anti-divers*” OR “Antidivers*” OR “Hostil* toward* divers*” OR “Anti-inclu*” OR “Anti-intervent*” OR “Hostil* toward* intervent*” OR “Anti-regulat*” OR “Antiregulat*” OR “Hostil* toward* regulat*” OR “Hostil* toward* inclu*” OR “Racism” OR “Racis*” OR “Scapegoating” OR “Xenophobia” OR “Sexism” OR “Elitis*” OR “Sovereignty” OR “Nationalis*” OR “Nativis*” OR “Pro-nativis*” OR “Pronativis*” OR “Anti-immigra*” OR “Hostil* toward* immigra*” OR “Anti-migra*” OR “Antimigra*” OR “Hostil* toward* migra*” OR “Anti-minorit*” OR “Antiminorit*” OR “Hostil* toward* minorit*” OR “Anti-ethnic*” OR “Antiethnic*” OR “Hostil* toward* ethnic*” OR “Hostil* toward* raci*” OR “Homophobia” OR “Homophobi*” OR “Transphobi*” OR “Anti-globali*” OR “Antiglobali*” OR “Hostil* toward* globali*” OR “Hostil* toward* the wealth*” OR “Anti-cosmopolitan” OR “Anticosmopolitan” OR “Far-right” OR “Anti-establishment” OR “Antiestablishment” OR “Hostil* toward* the establishment*” OR “Trust* in the establishment*” OR “Brexit” OR “Anti-expert*” OR “Antiexpert*” OR “Hostil* toward* expert*” OR “Trust* in expert*” OR “Anti-scien*” OR “Antiscien*” OR “Hostil* toward* scien*” OR “Trust* in scient*” OR “Hostil* toward* research*” OR “Trust* in research*” OR “Anti-business*” OR “Antibusiness*” OR “Hostil* toward* business)” OR “Trust* in business” OR “Anti-government” OR “Antigovernment” OR “Hostil* toward* government” OR “Trust* in government” OR “Culture war*” OR “Woke” OR “Conspiracy” OR “Mainstream media” OR “Antifa” OR “Antifacist*” OR “Social populism” OR “Far-left” OR “Farleft” OR “Anti-corporat*” OR “Anticorporat*” OR “Anti-left*” OR “Antileft*” OR “Anti-conservative*” OR “Anticonservative*” OR “Hostil* toward* the left*” OR “Hostil* toward* conservative*” AND “Abortion” OR “Sexual health” OR “Reproductive health” OR “Pregnan*” OR “Contracepti*” OR “Covid*” OR “Corona*” OR “SARS-CoV-2” OR “Pandemic” OR “Influenza” OR “Flu” OR “Facemask” OR “Face-mask” OR “Isolation” OR “Social distanc*” OR “Non-pharmac*” OR “Nonpharmac*” OR “Infection control*” OR “Vaccin*” OR “Anti-Vaccination Movement” OR “Vaccination Refusal” OR “Vaccination Hesitancy” OR “Immunis*” OR “Immuniz*” OR “MMR” OR “Measles” OR “Mumps” OR “Rubella” OR “Climate change prevention” OR “Environmental protection” OR “Pollution” OR “Emissions” OR “15-minute cit*” OR “fifteen-minute cit*” OR “road safety” OR “speed camera*” OR “enforcement camera*” OR “mental health” OR “mental illness*” OR “anxiety” OR “depress*” OR “physical* active*” OR “physical* exercise*” OR “diet” OR “nutrition” OR “obes*” OR “over-weight*” OR “overweight*” OR “cigarette*” OR “tobacco” OR “vaping” OR “vape*” OR “smok*” OR “Illegal drug*” OR “Illicit drug*” OR “Recreational drug*” OR “substance*” OR “alcohol*” OR “Screening*” OR “Gun control” OR “Firearm ban” OR “Gun-violence prevention” OR “Gun safety” OR “Gambling” OR “Betting” OR “Fluoridation” OR “Fluoridi?ation” OR “Health-care access” OR “Healthcare access” AND “Attitude*” OR “Belief*” OR “View*” OR “Acceptab*” OR “Adhere*” OR “Effect*” OR “Effective*” OR “Outcome*” OR “Impact*” OR “Benefit*” |

## Web of Science

| **Database name** | Web of Science (including Science Citation Index Expanded, Social Sciences Citation Index, Arts & Humanities Citation Index, and Emerging Sources Citation Index) |
| --- | --- |
| **Database platform** | https://www.webofscience.com |
| **Dates of database coverage** | 1970-present |
| **Date searched** | 19 February 2024 |
| **Number of results** | 9,724 |

| **#** | **Query** | **Results from 19 February 2024** |
| --- | --- | --- |
| 1 | TI = (Populis* or Illiberal* or Anti-liberal* or Antiliberal* or Anti-right* or Antiright* or Hostil* NEAR/2 right* or Hostil* NEAR/2 liberal* or Anti-EDI* or Hostil* NEAR/2 EDI* or Anti-equalit* or Antiequalit* or Hostil* NEAR/2 equalit* or Anti-divers* or Antidivers* or Hostil* NEAR/2 divers* or Anti-inclu* or Anti-intervent* or Hostil* NEAR/2 intervent* or Anti-regulat* or Antiregulat* or Hostil* NEAR/2 regulat* or Hostil* NEAR/2 inclu* or “Racism” or Racis* or “Scapegoating” or “Xenophobia” or “Sexism” or Elitis* or “Sovereignty” or Nationalis* or Nativis* or Pro-nativis* or Pronativis* or Anti-immigra* or Hostil* NEAR/2 immigra* or Anti-migra* or Antimigra* or Hostil* NEAR/2 migra* or Anti-minorit* or Antiminorit* or Hostil* NEAR/2 minorit* or Anti-ethnic* or Antiethnic* or Hostil* NEAR/2 ethnic* or Hostil* NEAR/2 raci* or “Homophobia” or Homophobi* or Transphobi* or Anti-globali* or Antiglobali* or Hostil* NEAR/2 globali* or Hostil* NEAR/2 wealth* or “Anti-cosmopolitan” or “Anticosmopolitan” or “Far-right” or “Anti-establishment” or “Antiestablishment” or Hostil* NEAR/2 establishment* or Trust* NEAR/2 establishment* or “Brexit” or Anti-expert* or Antiexpert* or Hostil* NEAR/2 expert* or Trust* NEAR/2 expert* or Anti-scien* or Antiscien* or Hostil* NEAR/2 scien* or Trust* NEAR/2 scient* or Hostil* NEAR/2 research* or Trust* NEAR/2 research* or Anti-business* or Antibusiness* or Hostil* NEAR/2 “business” or Trust* NEAR/2 “business” or “Anti-government” or “Antigovernment” or Hostil* NEAR/2 “government” or Trust* NEAR/2 “government” or Culture war* or “Woke” or “Conspiracy” or “Mainstream media” or “Antifa” or Antifacist* or “Social populism” or “Far-left” or “Farleft” or Anti-corporat* or Anticorporat* or Anti-left* or Antileft* or Anti-conservative* or Anticonservative* or Hostil* NEAR/2 left* or Hostil* NEAR/2 conservative*) or AB = (Populis* or Illiberal* or Anti-liberal* or Antiliberal* or Anti-right* or Antiright* or Hostil* NEAR/2 right* or Hostil* NEAR/2 liberal* or Anti-EDI* or Hostil* NEAR/2 EDI* or Anti-equalit* or Antiequalit* or Hostil* NEAR/2 equalit* or Anti-divers* or Antidivers* or Hostil* NEAR/2 divers* or Anti-inclu* or Anti-intervent* or Hostil* NEAR/2 intervent* or Anti-regulat* or Antiregulat* or Hostil* NEAR/2 regulat* or Hostil* NEAR/2 inclu* or “Racism” or Racis* or “Scapegoating” or “Xenophobia” or “Sexism” or Elitis* or “Sovereignty” or Nationalis* or Nativis* or Pro-nativis* or Pronativis* or Anti-immigra* or Hostil* NEAR/2 immigra* or Anti-migra* or Antimigra* or Hostil* NEAR/2 migra* or Anti-minorit* or Antiminorit* or Hostil* NEAR/2 minorit* or Anti-ethnic* or Antiethnic* or Hostil* NEAR/2 ethnic* or Hostil* NEAR/2 raci* or “Homophobia” or Homophobi* or Transphobi* or Anti-globali* or Antiglobali* or Hostil* NEAR/2 globali* or Hostil* NEAR/2 wealth* or “Anti-cosmopolitan” or “Anticosmopolitan” or “Far-right” or “Anti-establishment” or “Antiestablishment” or Hostil* NEAR/2 establishment* or Trust* NEAR/2 establishment* or “Brexit” or Anti-expert* or Antiexpert* or Hostil* NEAR/2 expert* or Trust* NEAR/2 expert* or Anti-scien* or Antiscien* or Hostil* NEAR/2 scien* or Trust* NEAR/2 scient* or Hostil* NEAR/2 research* or Trust* NEAR/2 research* or Anti-business* or Antibusiness* or Hostil* NEAR/2 “business” or Trust* NEAR/2 “business” or “Anti-government” or “Antigovernment” or Hostil* NEAR/2 “government” or Trust* NEAR/2 “government” or Culture war* or “Woke” or “Conspiracy” or “Mainstream media” or “Antifa” or Antifacist* or “Social populism” or “Far-left” or “Farleft” or Anti-corporat* or Anticorporat* or Anti-left* or Antileft* or Anti-conservative* or Anticonservative* or Hostil* NEAR/2 left* or Hostil* NEAR/2 conservative*) | 181,011 |
| 2 | TI = (“Abortion” or “Sexual health” or “Reproductive health” or Pregnan* or Contracepti* or Covid* or Corona* or “SARS-CoV-2” or “Pandemic” or “Influenza” or “Flu” or “Facemask” or “Face-mask” or “Isolation” or Social distanc* or Non-pharmac* or Nonpharmac* or Infection control* or Vaccin* or “Anti-Vaccination Movement” or “Vaccination Refusal” or “Vaccination Hesitancy” or Immunis* or Immuniz* or “MMR”or “Measles” or “Mumps” or “Rubella” or “Climate change prevention” or “Environmental protection” or “Pollution” or “Emissions” or 15-minute cit* or fifteen-minute cit* or “road safety” or speed camera* or enforcement camera* or “mental health” or mental illness* or “anxiety” or depress* or physical* active* or physical* exercise* or “diet” or “nutrition” or obes* or over-weight* or overweight* or cigarette* or “tobacco” or “vaping” or vape* or smok* or “Illegal” NEAR/2 drug* or “Illicit” NEAR/2 drug* or “Recreational” NEAR/2 drug* or substance* or alcohol* or Screening* or “Gun control” or “Firearm ban” or “Gun-violence prevention” or “Gun safety” or “Gambling” or “Betting” or “Fluoridation” or Fluoridi?ation or “Health-care access” or “Healthcare access”) or AB = (“Abortion” or “Sexual health” or “Reproductive health” or Pregnan* or Contracepti* or Covid* or Corona* or “SARS-CoV-2” or “Pandemic” or “Influenza” or “Flu” or “Facemask” or “Face-mask” or “Isolation” or Social distanc* or Non-pharmac* or Nonpharmac* or Infection control* or Vaccin* or “Anti-Vaccination Movement” or “Vaccination Refusal” or “Vaccination Hesitancy” or Immunis* or Immuniz* or “MMR”or “Measles” or “Mumps” or “Rubella” or “Climate change prevention” or “Environmental protection” or “Pollution” or “Emissions” or 15-minute cit* or fifteen-minute cit* or “road safety” or speed camera* or enforcement camera* or “mental health” or mental illness* or “anxiety” or depress* or physical* active* or physical* exercise* or “diet” or “nutrition” or obes* or over-weight* or overweight* or cigarette* or “tobacco” or “vaping” or vape* or smok* or “Illegal” NEAR/2 drug* or “Illicit” NEAR/2 drug* or “Recreational” NEAR/2 drug* or substance* or alcohol* or Screening* or “Gun control” or “Firearm ban” or “Gun-violence prevention” or “Gun safety” or “Gambling” or “Betting” or “Fluoridation” or Fluoridi?ation or “Health-care access” or “Healthcare access”) | 7,397,540 |
| 3 | TI = (Attitude* or Belief* or View* or Acceptab* or Adhere* or Effect* or Outcome* or Impact* or Benefit*) or AB = (Attitude* or Belief* or View* or Acceptab* or Adhere* or Effective* or Outcome* or Impact* or Benefit*) | 16,577,605 |
| 4 | #1 AND #2 AND #3 | 9,724 |

# Appendix 3: Study Characteristics – All Included Studies (n=238)

| **Author (Year)** | **Study Country (City or Region)** | **Study Design (Duration)** | **Sample Size and Participant characteristics** | **Focus of Populist Attitude(s)** | **Intervention Type(s)** | **Health Domain** | **Intervention Related Outcomes Reported** |
| --- | --- | --- | --- | --- | --- | --- | --- |
| Abad (2023)^3^ | United States | Qualitative (March-April, 2021) | 59 unvaccinated adults | Elites (Experts) | Vaccination | Infections - COVID-19 | Attitudes on uptake |
| Abadi (2021)^4^ | Germany, the Netherlands, Spain, the United Kingdom | Cross-sectional (April 2020) | 2,301 adults who passed attention check | Elites (Populist Attitudes) | Non-pharmaceutical infection controls | Infections - COVID-19 | Adherence to preventative measures |
| Adams-Clark (2022)^5^ | United States (Pacific Northwest) | Cross-sectional (Study 1: October-December 2020; Study 2: February-March 2021) | 308 undergraduate students in psychology or linguistics | Out-groups (Women) | Non-pharmaceutical infection controls | Infections - COVID-19 | Adherence to preventative measures |
| Adekola (2022)^6^ | Scotland (Glasgow) | Qualitative (March-April 2021) | 8 expert (academics/public health experts) and 18 non-expert members of an ethnic minority community | Elites (Government) | Vaccination | Infections - COVID-19 | Attitudes on uptake |
| Aechtner  (2022)^7^ | Australia | Cross-sectional (2018) | 1,287 adult participants in the Australian Survey of Social Attitudes (AuSSA); 1,003 adult participants in the Wellcome Global Monitor (WGM); both nationally representative | Elites (Government) | Vaccination | Infections - Childhood | Attitudes on uptake |
| Agaku (2023)^8^ | United States | Cross-sectional (December 2021-February 2022) | 211,303 adults from the Household Pulse Survey (HPS) | Elites (Government) | Vaccination | Infections - COVID-19 | Attitudes on uptake |
| Agnese (2022)^9^ | Italy | Cross-sectional (December 2020-March 2021) | 477 adult participants | Elites (Populist Attitudes) | Non-pharmaceutical infection controls | Infections - COVID-19 | Adherence to preventative measures |
| Ahluwalia (2021)^10^ | United States | Cross-sectional (March 2020) | 623 adults | Elites (Experts) | Non-pharmaceutical infection controls | Infections - COVID-19 | Adherence to preventative measures |
| Ahn (2023)^11^ | Republic of Korea | Cross-sectional (August 2020) | 304 adult panellists from the Market Link survey company | Elites (Experts) | Non-pharmaceutical infection controls | Infections - COVID-19 | Adherence to preventative measures |
| Alessandri (2020)^12^ | Italy | Cross-sectional (March-April 2020) | 1,520 adult participants in the Orientation toward Common Good” study (OCG-COVID-19) | Elites (Government) | Non-pharmaceutical infection controls | Infections - COVID-19 | Adherence to preventative measures |
| Allen (2012)^13^ | United States (Boston) | Qualitative (February-May 2008) | 64 White, Black, and Hispanic parents of daughters who were age eligible to receive the HPV vaccine (9–17 years) | Elites (Medical Professionals and Pharmaceutical Companies) | Vaccination | Infections - HPV | Attitudes on uptake |
| Allington (2022)^14^ | United Kingdom | Cross-sectional (April 2021) | 3,223 UK resident adults; nationally representative | Elites (Government & Scientists) | Vaccination | Infections - COVID-19 | Uptake of vaccine |
| Amundson (2022)^15^ | United States and Mexico (Texas-Mexico border) | Cross-sectional (March-May 2021) | 58 adult (predominantly Hispanic) patients in waiting areas of 4 federally qualiﬁed health centre (FQHC) clinics | Elites (Government) | Vaccination | Infections - COVID-19 | Attitudes on uptake |
| Ayalon (2021)^16^ | Israel | Cross-sectional (October 2021) | 376 Israeli adults | Elites (Government, Scientists, Medical Professionals, Healthcare System, Health Experts, Mass Media) | Non-pharmaceutical infection controls | Infections - COVID-19 | Adherence to preventative measures |
| Bacon (2023)^17^ | United States | Cross-sectional (June 2021) | 1,433 adults responding to a web-based survey (note: reporting of sample size seems to vary across the paper) | Elites (Health care system) | Vaccination | Infections - COVID-19 | Uptake of vaccine |
| Baek (2022)^18^ | Republic of Korea | Cross-sectional (April 2020) | 1,207 adults responding to an anonymous online survey | Elites (Government, Medical Professionals and Mass Media) | Non-pharmaceutical infection controls | Infections - COVID-19 | Adherence to preventative measures |
| Bajos (2022)^19^ | France (metropolitan areas) | Cross-sectional (July 2021) | 80,971 adults taking part in the EpiCov population-based cohort study | Elites (Government & Scientists) | Vaccination | Infections - COVID-19 | Uptake of vaccine |
| Ball (2021)^20^ | United States | Cross-sectional (no date given) | 268 American adults | Checks on Popular Sovereignty (Personal Freedoms) | Non-pharmaceutical infection controls | Infections - COVID-19 | Adherence to preventative measures |
| Barbieri (2022)^21^ | Italy (Autonomous Province of Bolzano, South Tyrol) | Cross-sectional (March 2021) | 1,425 adults as part of a probability-based sample | Elites (Government and Health Authorities) | Vaccination | Infections - COVID-19 | Uptake of vaccine |
| Baumann (2022)^22^ | United States (Miami, New Orleans, Boston, Detroit, Durham, Philadelphia, Camden, and Houston) | Cross-sectional (June-August 2021) | 1,298 parents of children aged 3-16 years in one of nine paediatric emergency departments | Elites (Scientists and Mass Media) | Vaccination | Infections - COVID-19 | Uptake of vaccine by parents and children |
| Baumgaertner (2018)^23^ | United States | Cross-sectional (January 2017) | 1,006 adult respondents; nationally representative, sample from Survey Sampling International (SSI) (Note: Sample size is taken from the number of respondents send the survey as this is the only number reported) | Elites (Government, Medical and Health Professionals) | Vaccination | Infections - Pertussis, Measles and Influenza | Attitudes on vaccines |
| Bendetson (2023)^24^ | United States (Southwest Virginia) | Cross-sectional (March 2021) | 2,459 adult employees of a not-for-profit healthcare system in Southwest Virginia | Elites (Scientists) | Vaccination | Infections - COVID-19 | Uptake of vaccine |
| Bianco (2019)^25^ | Italy (Catanzaro and Cosenza regions) | Cross-sectional (April-June 2017) | 575 parents of children aged 1-5 attending kindergarten | Elites (Pharmaceutical Companies) | Vaccination | Infections - Childhood | Attitudes and uptake of childhood vaccines |
| Bickham (2021)^26^ | United States | Cross-sectional (June-July 2020) | 285 adult residents of the US, recruited through Facebook | Elites (Government) | Non-pharmaceutical infection controls | Infections - COVID-19 | Adherence to preventative measures |
| Birmingham Voluntary Service Council Research (2021)^27^ | United Kingdom (Birmingham) | Qualitative (no date reported) | 27 adults identified through local organisations who support people with substance use issues, homelessness, mental ill health and contact with the criminal justice system | Elites (Government, Scientists, Pharmaceutical companies, and Medical Professionals) | Vaccination | Infections - COVID-19 | Views on uptake of the COVID-19 vaccine |
| Blackburn (2023)^28^ | Colombia, Costa Rica, Czech Republic, Denmark, Estonia , Finland, Germany, Ireland, Italy, Japan, New Zealand, Norway , Portugal, Slovakia, Spain, Sweden, Switzerland, Turkey, the United Kingdom and the United States | Cross-sectional (Summer 2021) | Colombia (n=548), Costa Rica (n=270), Czech Republic (n=365), Denmark (n=127), Estonia (n=246), Finland (n=963), Germany (n=152), Ireland (n=401), Italy (n=310), Japan (n=2,133), New Zealand (n=38), Norway (n=376), Portugal (n=484), Slovakia (n=313), Spain (n=575), Sweden (n=134), Switzerland (n=593), Turkey (n=200), United Kingdom (n=134), United States (n=114); all were adult participants of the COVIDiSTRESS II Global Survey who passed an attention check | Elites (Government, Experts and Scientists) | Non-pharmaceutical infection controls | Infections - COVID-19 | Adherence to preventative measures |
| Blanchi (2021)^29^ | France (Le Mans and Paris) and Italy (Cagliari and Pavia) | Cross-sectional (January-March 2021) | 417 adult patients on dialysis at four large dialysis facilities | Elites (Scientists and Authorities) | Vaccination | Infections - COVID-19 | Uptake of vaccine |
| Bogart (2021)^30^ | United States (Los Angeles County) | Cross-sectional (May-July 2020) | 101 HIV-positive Black American adults enrolled in an RCT of a community-based ART adherence intervention | Elites (Government) | Vaccination | Infections - COVID-19 | Attitudes on uptake |
| Borjesson (2014)^31^ | Sweden | Cross-sectional (April-August 2010) | 1,587 Swedish adults | Elites (Government) | Vaccination | Infections - H1N1 | Attitudes on uptake and adherence to preventative measures |
| Boyle (2022)^32^ | United States | Cross-sectional (August 2021) | 1000 US adult respondents from the MFour mobile panel, a U.S. nonprobability market research smartphone panel | Elites (Government, Health Experts and Mass Media) | Vaccination | Infections - COVID-19 | Uptake of vaccine |
| Bozkurt (2023)^33^ | Türkiye | Cross-sectional (no date given) | 4,004 internet users across Türkiye (note: ages not given) | Elites (Government. Scientists, Medical Professionals and Pharmaceutical Companies) | Vaccination | Infections - COVID-19 | Attitudes on vaccine and uptake of vaccine |
| Bruder (2022)^34^ | Germany | Cross-sectional (May 2020) | 1,013 adults from the 11th wave of the German national COVID-19 Snapshot Monitoring (COSMO) survey; nationally representative | Elites (Government) | Non-pharmaceutical infection controls | Infections - COVID-19 | Adherence to preventative measures |
| Burton (2023)^35^ | United Kingdom | Qualitative (May-November 2020) | 116 adults across the UK; purposively selected based on one of the following characteristics: had mental health condition(s) or long-term health condition(s), were parents of young children, or were older (aged 70+), and younger (aged 18–24) adults | Elites (Government) | Non-pharmaceutical infection controls | Infections - COVID-19 | Adherence to preventative measures, particularly social distancing |
| Butler (2022)^36^ | United States (San Francisco) | Qualitative (January-February 2021) | 109 adult residents of San Francisco who identified as Black/African Americans, Latinx or Chinese Americans | Elites (Government and Pharmaceutical Industry and Health Institutions) | Vaccination | Infections - COVID-19 | Uptake of vaccine |
| Bystrom (2014)^37^ | Sweden (Stockholm) | Qualitative (February-May 2013) | 20 parents living in anthroposophic communities | Elites (Healthcare System, Medical Professionals and Experts) | Vaccination | Infections - MMR | Attitudes towards and uptake of vaccine |
| Caceres (2022)^38^ | United States (Los Angeles County) | Qualitative (February 2021) | 22 adult promotoras (Community Health Workers) | Elites (Government) | Vaccination | Infections - COVID-19 | Views on the COVID-19 vaccine |
| Capasso (2022)^39^ | Italy | Cross-sectional (March-May 2021) | 822 unvaccinated Italian adults | Elites (Government & Scientists) | Vaccination | Infections - COVID-19 | Attitudes on uptake |
| Carlson (2022)^40^ | Australia (Perth, Western Australia) | Qualitative (August-October 2021) | 11 interviews and 3 focus groups totally 37 participants, all culturally and linguistically diverse (CALD) adults | Elites (Government and Medical Professionals) | Vaccination | Infections - COVID-19 | Attitudes on uptake |
| Caserotti (2022)^41^ | Italy | Cross-sectional (January-February 2021) | 5,006 Italian adults; nationally representative | Elites (Health Institutions and Mass Media) | Vaccination | Infections - COVID-19 | Attitudes on uptake |
| Chayinska (2022)^42^ | Türkiye and Germany | Cross-sectional (May-June 2020) | 290 adults in Türkiye and 408 adults in Germany; recruited using convenience sampling via posts on Facebook | Elites (Scientists) | Non-pharmaceutical infection controls | Infections - COVID-19 | Adherence to preventative measures, particularly social distancing |
| Chen (2022)^43^ | United States | Cross-sectional (late 2020) | 531 US adults; nationally representative | Elites (Government) | Non-pharmaceutical infection controls | Infections - COVID-19 | Adherence to preventative measures |
| Chen (2022)^44^ | United States | Repeat cross-sectional (January 2021-May 2022) | 250 million US adults participating in the Household Pulse Survey (HPS) for Americans | Elites (Government) | Vaccination | Infections - COVID-19 | Uptake of vaccine |
| Cherniak (2023)^45^ | United States | Cross-sectional (March-April 2020) | 1,141 Jewish American adults, recruited via email lists, social media and websites of Jewish organisations | Elites (Scientists and Medical Professionals) | Non-pharmaceutical infection controls | Infections - COVID-19 | Adherence to preventative measures |
| Choi (2022)^46^ | United States | Repeat Cross-sectional (December 2020-July 2021) | 5,446 US adult participants in the Understanding America Survey; nationally representative | Elites (Government and Health Institutions) | Vaccination | Infections - COVID-19 | Attitudes towards and actual uptake of vaccine |
| Choi (2024)^47^ | Australia, Finland, Italy, South Korea, Sweden, and the United States | Cross-sectional (October-November 2020) | 3,065 adults; Australia (n = 500), Finland (n = 554), Italy (n = 500), South Korea (n = 506), Sweden (n = 505), and the United States (n = 500) | Elites (Government) | Non-pharmaceutical infection controls | Infections - COVID-19 | Adherence intentions |
| Choma (2021)^48^ | Canada, United Kingdom and United States | Cross-sectional (March 2020) | 1,1123 Canadian, US or UK adults (n= 289 in Canada, n= 308 in USA, and n=526 in UK) | Elites (Government, Scientists, Mainstream Media, Academia, Health Experts) | Non-pharmaceutical infection controls | Infections - COVID-19 | Adherence to preventative measures |
| Cizmar (2023)^49^ | United States | Repeat cross-sectional (2012, 2016, 2020) | Between 4.270 and 15,729 US voters (18+) using data from the American National Election Studies (ANES) | Out-groups (Women) | Sexual/reproductive health care | Abortion | Attitudes on abortion |
| Clark (2008)^50^ | United States (Houston) | Cross-sectional (March-December 2005) | 113 HIV+ adult patients (diagnosed in the last three years) in four public health facilities | Elites (Government and Pharmaceutical Industry) | Sexual/reproductive health care | Infections - HIV | Uptake and adherence to HAART |
| Corcoran (2023)^51^ | United States | Cross-sectional (May-June 2021) | 1,734 US adults; nationally representative using the AmeriSpeak® probability-based panel | Elites (Scientists) | Vaccination | Infections - COVID-19 | Attitudes and uptake of vaccine |
| Cross (2023)^52^ | United States (Michigan) | Qualitative (December 2020-June 2021) and Cross-sectional (January-March 2021) | 40 interviews with community members across 4 Michigan counties; 1598 surveys, representative of the Detroit population; all participants are Black or Latinx adult Michiganders | Elites (Scientists and Healthcare Professionals) | Vaccination | Infections - COVID-19 | Uptake of vaccine |
| Cunningham-Erves (2018)^53^ | United States (Alabama) | Qualitative (December 2020-June 2021) | 9 interviews with Black mothers of daughters 9-12 years; convenience sample recruited via community sites and community gatekeepers | Elites (Medical Professionals and Pharmaceutical Companies) | Vaccination | Infections - HPV | Uptake of HPV vaccine (mothers’ intentions to have their daughter vaccinated) |
| Dell'Imperio (2023)^54^ | United States (Michigan, Illinois, South Carolina, Louisiana, Georgia) | Qualitative (May-September 2021) | 15 interviews with Black adults with long COVID symptoms (physical or mental) | Elites (Government) | Vaccination | Infections - COVID-19 | Views on the COVID-19 vaccine and vaccine uptake |
| Denford (2022)^55^ | United Kingdom | Qualitative (September-October 2021) | 70 UK adults , 35 (ages 18-29) who were unvaccinated and 35 (ages 30-49) who had received one dose of the vaccine; recruitment via social media and survey company (M3 Global Research) | Elites (Government) | Vaccination | Infections - COVID-19 | Uptake of vaccine |
| Dennis (2021)^56^ | United Kingdom (Northwest England) | Qualitative (April 2021) | 10 adult care home employees who had been invited to have (but had not received) the COVID-19 vaccine | Elites (Government and Healthcare System) | Vaccination | Infections - COVID-19 | Attitudes on uptake |
| Di Napoli (2023)^57^ | Italy | Cross-sectional (November-December 2020) | 324 Italian adults; convenience sample recruited via social networks | Elites (Scientists, Health Policymakers and Healthcare Institutions) | Vaccination | Infections - COVID-19 | Uptake of COVID-19 and other vaccines for self and children (real and hypothetical) |
| Dohle (2020)^58^ | Germany | Cross-sectional (Study 1: March 2020; Study 2: April-May 2020) | 962 German adults (Study 1 n= 661; Study 2 = 301 ) | Elites (Government & Scientists) | Non-pharmaceutical infection controls | Infections - COVID-19 | Attitudes towards and adherence to preventative measures |
| Dupuis (2021)^59^ | United States | Cross-sectional (no date given) | 516 US adults, recruited via Amazon's Mechanical Turk | Elites (Government and Mass Media) | Vaccination | Infections - COVID-19 | Uptake of vaccine |
| Ebrahimi (2021)^60^ | Norway | Cross-sectional (January-February 2021) | 4,571 Norwegian adults | Elites (Government and Health Authorities) | Vaccination | Infections - COVID-19 | Attitudes on uptake |
| Ehrke (2023)^61^ | Germany and Poland | Cross-sectional (April-May 2020); Longitudinal (April-June 2020) | 1,090 adult participants in cross-sectional study (n =617 in Germany and n =473 in Poland), sub-sample of 216 participants (n =143 in Germany and n =73 in Poland) in longitudinal study; recruited using convenience sampling | Elites (Populist Attitudes, Political Institutions, Scientific Institutions and Mass Media) | Non-pharmaceutical infection controls | Infections - COVID-19 | Acceptance of and adherence to preventative measures |
| Enders (2022)^62^ | United States | Cross-sectional (July-August 2021) | 2,065 US adults; nationally representative to match the 2019 US Census American Community Survey; recruited via Qualtrics | Elites (Scientists) | Vaccination | Infections - COVID-19 | Uptake of vaccine |
| Eraso (2021)^63^ | United Kingdom (North London: Islington, Har- ingey, Camden, Hackney, Barnet and Enfield) | Cross-sectional (May 2020); Qualitative (August-September 2020) | 681 adult survey respondents, recruited via convenience sampling, 30 interview participants, purposively sampled based on relevant socio-demographic groups | Elites (Government) | Non-pharmaceutical infection controls | Infections - COVID-19 | Adherence to preventative measures, specifically social distancing |
| Eshel (2022)^64^ | Israel | Cross-sectional (October 2021) | 2,002 Jewish Israeli adults; sampled from 65,000 potential panellists connected to an online survey company | Elites (Government) | Vaccination | Infections - COVID-19 | Uptake of vaccine |
| Eshel (2022)^65^ | Israel | Cross-sectional (October 2021) | 2,002 Jewish Israeli adults; nationally representative | Elites (Government) | Vaccination | Infections - COVID-19 | Uptake of vaccine |
| Farhart (2022)^66^ | United States | Repeat cross-sectional (October 2021; October-November 2021) | 1,080 adults in Wave 1, 1,140 adults in Wave 2; participants recruited pre- and post-2020 presidential election via Amazon's Mechanical Turk (Wave 1) and Forthright survey company (Wave 2) | Elites (Intellectuals) | Vaccination | Infections - COVID-19 | Attitudes on vaccine |
| Fattorini (2023)^67^ | Italy (Autonomous Province of Trento) | Qualitative (February-May 2018) | 21 parents who are members of *Vaccinare Informati*, an organisation that advocates for freedom of choice in vaccination decisions; identified through snowball sampling | Elites (Scientists) | Vaccination | Infections - Childhood | Attitudes on childhood vaccine |
| Fernández‐Penny (2021)^68^ | United States (Philadelphia) | Cross-sectional (January-May 2021) | 1,068 adult patients with stable clinical status in emergency departments at the Hospital of the University of Pennsylvania (HUP) and Penn Presbyterian Medical Center (PPMC) | Elites (Government and Mass Media) | Vaccination | Infections - COVID-19 | Attitudes on vaccine uptake |
| Fischer (2016)^69^ | United States (Arkansas, Colorado, Maine, and Wisconsin) | Qualitative (June 2013-July 2014) | 25 rural veterans and 11 rural mental health care providers to veterans, representing 4 VA regions (Veterans Integrated Service Networks [VISNs]); all participants were adults, veterans had to live at least 50 miles from their nearest Veterans Affairs Medical Center (VAMC) | Elites (Government (VA System) and Medical Professionals) | Health care | Mental health | Uptake of mental health services |
| Fleming (2017)^70^ | United States and Mexico (San Diego and Tijuana) | Cross-sectional (September 2010-October 2012) | 400 adult male clients of female sex workers (FSWs); participants were enrolled in a sexual risk reduction intervention known as Hombre Seguro (‘‘Safe Men’’), half from San Diego and half from Tijuana; time-location based sampling | Out-groups (Women) | Disease screening | Infections - HIV | Uptake of HIV testing |
| Ford (2013)^71^ | United States (Los Angeles) | Cross-sectional (August 2006-May 2007) | 226 adult participants, aged 50+ and participating in the LA VOICES study, which includes socially vulnerable, racially/ethnically diverse men and women living in Los Angeles | Elites (Government) | Disease screening | Infections - HIV | Uptake of HIV testing |
| Frew (2012)^72^ | United States (Atlanta) | Cross-sectional (September-December 2009) | 503 US adults; recruited based on venue-based sampling, including churches, bookstores, educational forums, community meetings, and special events such as family health fairs | Elites (Government) | Vaccination | Infections - H1N1 and seasonal flu | Attitudes on vaccine uptake |
| Fridman (2020)^73^ | United States | Cross-sectional (April 2020) | 1,243 US adults, representative by age, gender and regions of the US (New England, Mid-Atlantic, East North Central, West North Central, South Atlantic, East South Central, West South Central, Mountain, and Pacific); recruited by Qualtrics Panels | Elites (Government) | Non-pharmaceutical infection controls | Infections - COVID-19 | Adherence to preventative measures, specifically social distancing |
| Frietze (2023)^74^ | United States (El Paso, Texas) | Cross-sectional (June-August 2020) | 602 predominantly Hispanic adults living in the US-Mexico border town of El Paso, Texas | Elites (Government) | Vaccination | Infections - HPV | Acceptance and uptake of HPV vaccine |
| Galasso (2022)^75^ | Australia, Austria, France, Germany, Italy, New Zealand, Sweden, the United Kingdom and the United States | Longitudinal (December 2020-July 2021) | 6,379 adult participants; n=343 in Australia, n=324 in Austria, n=850 in France, n= 1,481 in Germany, n= 710 in Italy, n= 639 in New Zealand, n= 693 in Sweden, n= 697 in the UK, and n= 642 in the US; nationally representative | Elites (Scientists) | Vaccination | Infections - COVID-19 | Attitudes towards and uptake of vaccine |
| Geana (2021)^76^ | United States (Midwest) | Qualitative (March 2021) | 25 adult interview participants, all women recently released from jail | Elites (Government, Pharmaceutical Industry, Medical Establishment) | Vaccination | Infections - COVID-19 | Attitudes towards uptake of vaccine |
| Gehlbach (2022)^77^ | United States (Eastern Coachella Valley of Southern California) | Qualitative (August 2020-January 2021) | 55 adult members of Latinx and Indigenous Mexican farm-working communities | Elites (Government) | Vaccination, disease screening | Infections - COVID-19 | Attitudes towards testing and vaccination |
| Gilles (2011)^78^ | Switzerland | Longitudinal (March 2009-June 2010) | 601 French-speaking Swiss adults | Elites (Government, Pharmaceutical Companies, Health Experts, Medical Professionals) | Vaccination, non-pharmaceutical infection controls | Infections - H1N1 | Uptake of vaccine and attitudes towards preventative measures |
| Goldfinch (2022)^79^ | United States and Japan | Cross-sectional (no date given) | 2,063 adult participants (1044 in the US, 1019 in Japan); "broadly representative" samples, recruited via the survey company Dynata | Elites (Government) | Non-pharmaceutical infection controls | Infections - COVID-19 | Adherence to preventative measures, including mask wearing, social distancing and uptake of a contact tracing app |
| Goodwin (2022)^80^ | Israel | Cross-sectional (December 2020-January 2021) | 1,011 adult participants; nationally representative, recruited using survey company iPanel | Elites (Government) | Vaccination | Infections - COVID-19 | Acceptance of the vaccine |
| Goren (2022)^81^ | Israel | Repeat cross-sectional (April 2020, October 2020, December 2020, November 2021) | 3,732 adult participants, representing members of the general Jewish majority, Arab community and Ultra-Orthodox community | Elites (Government) | Non-pharmaceutical infection controls | Infections - COVID-19 | Attitudes towards adhering to preventative measures |
| Gothreau (2022)^82^ | United States | Cross-sectional (Winter 2018; Fall 2016) | Study 1: 1,400 US adults; recruited via the survey company Prolific; Study 2: 4,270 adult respondents from the nationally representative 2016 American National Election Study | Out-groups (Women) | Sexual/reproductive health care | Abortion and Birth control | Attitudes towards women's abortion and birth control uptake |
| Gray (2022)^83^ | United States (Montgomery, Alabama) | Cross-sectional (January 2021) | 1,000 adult residents of Montgomery; representative sample, recruited by Consensus Strategies for Partners in Health (PIH-USA) | Elites (Government and Medical Professionals) | Vaccination | Infections - COVID-19 | Attitudes towards uptake of vaccine |
| Grezo (2022)^84^ | Slovakia | Cross-sectional (no date given, assumption is 2021) | 600 adult Slovaks; gender-based representative sample recruited bya survey company | Elites (Government) | Non-pharmaceutical infection controls | Infections - COVID-19 | Adherence to preventative measures |
| Guazzini (2021)^85^ | Italy | Cross-sectional (October-November 2020) | 501 participants; recruited via social media | Elites (Government) | Non-pharmaceutical infection controls | Infections - COVID-19 | Uptake of a contact tracing application |
| Guillon (2020)^86^ | France | Cross-sectional (April-May 2020) | 1,849 adult respondents; recruited via Facebook | Elites (Government) | Non-pharmaceutical infection controls | Infections - COVID-19 | Attitudes towards and uptake of preventative measures during the pandemic, specific focus on quarantining |
| Guillon (2022)^87^ | France | Cross-sectional (November 2020) | 1,042 French adults; representative sample | Elites (Government) | Non-pharmaceutical infection controls | Infections - COVID-19 | Uptake of contact tracing application |
| Hafner-Fink (2021)^88^ | Slovenia | Cross-sectional (April-May 2020) | 2,000 Slovenian adults; representative sample | Elites (Government) | Non-pharmaceutical infection controls | Infections - COVID-19 | Adherence to preventative measures |
| Hamada (2015)^89^ | Japan (Fukuoka prefecture) | Cross-sectional (November 2012-April 2013) | 1,407 mothers of daughters aged 13-16 years in two middle schools and ten high schools in Fukuoka prefecture | Elites (Government) | Vaccination | Infections - HPV | Acceptance of HPV vaccine for adolescent daughters |
| Hartman (2021)^90^ | United Kingdom | Repeat cross-sectional (March 2020 and April-May 2020) | 1,406 UK adults; nationally representative, recruited by Qualtrics | Elites (Scientists) | Non-pharmaceutical infection controls | Infections - COVID-19 | Motivation to adhere to preventative measures, specifically social distancing |
| Hill (2023)^91^ | Switzerland | Cross-sectional (February-June 2021) | 2,328 Swiss adults; nationally representative, data from the Measurement and Observation of Social Attitudes in Switzerland study | Elites (Medical Professionals, Scientists, Mass Media, Government, Business and Industry) | Vaccination, non-pharmaceutical-based infection controls | Infections - COVID-19 | Attitudes towards vaccines and adherence to preventative measures |
| Hills (2021)^92^ | United Kingdom (North London) | Cross-sectional (May 2020) | 681 adult residents of North London (Islington, Haringey, Camden, Hackney, Barnet or Enfield councils) | Elites (Government) | Non-pharmaceutical infection controls | Infections - COVID-19 | Adherence to preventative measures |
| Hong (2023)^93^ | United States (Los Angeles) | Cross-sectional (May-October 2021) | 249 Gay, Bisexual, and Other Men Who Have Sex with Men (GBMSM) with a history of substance abuse | Elites (Government) | Vaccination | Infections - COVID-19 | Uptake of vaccine |
| Hori (2023)^94^ | Japan | Cross-sectional (September-October 2022) | 26,313 Japanese adults from the Japan COVID-19 and Society Internet Survey, which is "close to nationally representative" | Elites (Government) | Vaccination | Infections - mpox | Attitudes on vaccine uptake |
| Hosokawa (2022)^95^ | Japan | Cross-sectional (July-August 2021) | 1,621 pregnant Japanese adults who expected to give birth by December 2021; data from the Japan COVID-19 and Society Internet Survey | Elites (Government) | Vaccination | Infections - COVID-19 | Uptake of vaccine |
| Huang (2022)^96^ | United States (Atlanta) | Qualitative (February-June 2021) | 29 interviews among a majority Black sample of US adults ; purposive sampling of households that declined participation in a national COVID-19 serosurvey | Elites (Government) | Vaccination | Infections - COVID-19 | Attitudes on vaccine uptake |
| Huang (2024)^97^ | United States (Southeastern) | Cross-sectional (March-June 2021) | 224 unvaccinated adult US students attending a Historically Black College and University (HBCU) | Elites (Government, Health and Medical Professionals and the Health System) | Vaccination | Infections - COVID-19 | Attitudes on vaccine uptake |
| Hubble (2022)^98^ | United States (North Carolina) | Cross-sectional (April-May 2021) | 860 adult EMS professionals | Elites (Government) | Vaccination | Infections - COVID-19 | Uptake of vaccine |
| Jach (2023)^99^ | Poland | Cross-sectional (February 2021) | 1,286 Polish adults; representative sample of Polish internet users | Elites (Scientists) | Vaccination, non-pharmaceutical-based infection controls | Infections - COVID-19 | Attitudes towards vaccines and adherence to preventative measures |
| Jamison (2019)^100^ | United States (Maryland and Washington, D.C.) | Qualitative (2012-2014) | 119 Black and white US adults; purposive sampling | Elites (Government, Pharmaceutical Companies and Healthcare System) | Vaccination | Infections - Influenza | Uptake of influenza vaccine |
| Jennings (2021)^101^ | United Kingdom (National; Bristol and Oldham) | Cross-sectional and Qualitative (November-December 2020) | 1,476 UK adults in quantitative survey (nationally representative); 29 focus group participants in Bristol and Oldham | Elites (Government, Health Experts and Health Institutions) | Vaccination | Infections - COVID-19 | Uptake of vaccine |
| Jennings (2023)^102^ | France, Germany and Spain | Cross-sectional (September-November 2021 in France; October-November 2021 in Germany; September 2021 in Spain) | 1,548 in France, 1,558 in Germany, 1,022 in Spain; all nationally representative | Elites (Government) | Vaccination | Infections - COVID-19 | Attitudes on uptake of vaccine |
| Jeong (2024)^103^ | Republic of Korea | Cross-sectional (March 2021) | 600 adult participants; 120 each from the five major metropolitan areas of South Korea | Elites (Government) | Non-pharmaceutical infection controls | Infections - COVID-19 | Adherence to preventative measures, specifically handwashing and mask wearing |
| Jiang (2022)^104^ | United States | Repeat cross-sectional (June and August 2020) | 492 employed adults; representative of 48 states plus the District of Columbia, recruited via Amazon's Mechanical Turk (MTurk) | Elites (Government) | Non-pharmaceutical infection controls | Infections - COVID-19 | Attitudes towards and adherence to preventative measures |
| Jimenez (2021)^105^ | United States (New Jersey: Essex, Middlesex, Passaic, and Union counties) | Qualitative (November 2020-February 2021) | 111 Black and Latinx adult participants; recruited via partnership with 18 community-based organizations and 4 health care organisations; group interviews included participants who worked in health care settings as ancillary or support staff (n=88/111) | Elites (Government) | Disease screening, non-pharmaceutical infection controls | Infections - COVID-19 | Attitudes towards preventative measures (specifically mask wearing) and testing |
| Johnson (2023)^106^ | United States | Cross-sectional (February 2021) | 803 US adults; recruited via Forthright online panel and nationally representative | Elites (Authorities) | Vaccination | Infections - COVID-19 | Uptake of vaccine |
| Jones (2022)^107^ | United States | Cross-sectional (March and June 2021) | Sample sizes not reported; Study 2 included only white US adults; participants were recruited by Qualtrics | Elites (Government) | Vaccination | Infections - COVID-19 | Uptake of vaccine |
| Jung (2022)^108^ | France | Cross-sectional (May-June 2021) | 1,941 French adults aged 65+; broadly representative of the senior citizen population in France | Elites (Government, Pharmaceutical Companies, Medical and Health Professionals, Experts, Mass Media) | Vaccination | Infections - COVID-19 | Uptake of vaccine |
| Karaarslan-Semiz (2023)^109^ | Türkiye | Cross-sectional (April-July 2021) | 434 Turkish pre-service teachers from 19 universities in six geographical regions around the country | Elites (Scientists) | Vaccination | Infections - COVID-19 | Attitudes towards uptake of vaccine |
| Kim (2023)^110^ | United States | Cross-sectional (October 2020) | 1,400 US adults; nationally representative, recruited by Qualtrics | Elites (Government and Public Health Experts) | Non-pharmaceutical infection controls | Infections - COVID-19 | Adherence to preventative measures |
| Kim (2023)^111^ | United States | Cross-sectional (October-November 2020) | 500 US adults; nationally representative, recruited by Qualtrics | Elites (Government) | Non-pharmaceutical infection controls | Infections - COVID-19 | Adherence to preventative measures |
| Kohler (2023)^112^ | Germany and Austria | Cross-sectional (May-June 2021)* Initially reported as 2020, but tables and the fact they are capturing data on people's COVID-19 vaccine status makes me think this is a typo and it's 2021 | 870 adult participants; data collected via Clickworker | Elites (Scientists) | Vaccination | Infections - Various | Uptake of COVID-19, MMR, tick-borne encephalitis (TBE), HPV, seasonal influenza (SI) and meningococcal disease (MD) vaccines |
| Kosic (2024)^113^ | Italy | Cross-sectional (December 2020 - May 2021) | 390 Italian adults; convenience sampling | Elites (Government and Populist Attitudes) | Vaccination, non-pharmaceutical infection controls | Infections - COVID-19 | Attitudes towards vaccines and adherence to preventative measures |
| Koskan (2023)^114^ | United States (Unnamed southwestern state) | Qualitative (2022) | 30 adults living in rural regions of one southwestern US state; recruited via purposive sampling | Elites (Government, Scientists, Health and Medical Professionals, Mass Media) | Vaccination | Infections - COVID-19 | Attitudes towards vaccines |
| Kossowska (2021)^115^ | Poland | Cross-sectional (March 2020, June 2020) | Study 2: 426 Polish adults ; Study 3: 376 Polish adults; both recruited via the Pollster Research Institute; semi-nationally representative | Elites (Scientists) | Vaccination | Infections - Various | Attitudes towards vaccines |
| Kowalski (2022)^116^ | Germany | Cross-sectional (January-April 2021) | 224 home-isolated individuals with acute SARS-CoV-2 infection | Elites (Government) | Vaccination | Infections - COVID-19 | Attitudes towards uptake of vaccine |
| Krastev (2023)^117^ | Canada | Cross-sectional (April-May 2021) | 1,541 Canadian adults; recruited via survey company Dynata or Asking Canadians; nationally representative | Elites (Government, Police, Healthcare and Medical Professionals, Scientists, Pharmaceutical Companies, Mass Media, Healthcare System, Health Experts) | Vaccination | Infections - COVID-19 | Uptake of vaccines (COVID-19 and generally) |
| Krupenkin (2021)^118^ | United States | Cross-sectional (October 2009 and April 2015) | H1N1: 1,004 US adults (October 2009), nationally representative; Measles: 4,570 US adults (April 2015) | Elites (Government) | Vaccination | Infections - Various | Attitudes and uptake of smallpox, H1N1 and measles vaccines |
| Kuhn (2021)^119^ | United States (Los Angeles) | Longitudinal (December 2020-February 2021) | 90 adults experiencing homelessness in Los Angeles | Elites (Government and Mass Media) | Vaccination | Infections - COVID-19 | Attitude towards and/or actual uptake of vaccine |
| Kuroki (2022)^120^ | Japan | Cross-sectional (December 2020) | 1,248 Japanese adults; nationally representative | Elites (Government) | Non-pharmaceutical infection controls | Infections - COVID-19 | Adherence to preventative measures and uptake of contact tracing app |
| Ladini (2023)^121^ | Italy | Cross-sectional (April-July 2020) | 13,944 Italian adults (taken from Table 1) | Elites (Government) | Non-pharmaceutical infection controls | Infections - COVID-19 | Attitudes towards preventative measures |
| Lalot (2023)^122^ | United Kingdom | Cross-sectional (May 2020, July 2020) | Study 1 (July 2020); 2,413 UK adults, NatCen data, nationally representative; Study 2 (May 2020): 1,523 UK adults, recruited by Qualtrics nationally representative by gender and age | Elites (Government) | Non-pharmaceutical infection controls | Infections - COVID-19 | Attitudes towards adherence to preventative measures |
| Latkin (2023)^123^ | United States | Longitudinal (March 2020-November 2021) | 493 US adults in Wave 6, 390 in Wave 7; data from the COVID-19 and Well-Being Study | Elites (Government, Scientists and Mass Media) | Vaccination | Infections - COVID-19 | Uptake of vaccine |
| Lee (2016)^124^ | United States (Colorado, Massachusetts, Missouri and Washington) | Cross-sectional (2002-2003) | 1,253 parents of school children in four US states, who both had and didn't have a non-medical exemption (NME) for vaccinating their children | Elites (Government and Healthcare Professionals) | Vaccination | Infections - Childhood | Attitudes and uptake of vaccines for children |
| Lee (2022)^125^ | United States | Repeat cross-sectional (July and December 2020) | 1,119 US adults in Wav e 1 (July 2020), 543 US adults in Wave 2 (December 2020); recruitment via Dynata, nationally representative | Elites (Government, Scientists, Mass Media and Populist Attitudes) | Non-pharmaceutical infection controls | Infections - COVID-19 | Attitudes towards adherence to preventative measures, specifically mask-wearing |
| Lehr (2021)^126^ | United States | Repeat cross-sectional (December 2018; March, May, June; June and September 2020) | Between 8,298 and 7,235 US adults (survey wave dependent); data from the Pew Research Center’s American Trends Panel (ATP); nationally representative | Elites (Government and Mass Media) | Vaccination | Infections - COVID-19 | Attitudes towards adherence to preventative measures, specifically mask-wearing |
| Lello (2022)^127^ | Italy | Qualitative (December 2020- May 2022) | 67 Italian adults who expressed scepticism or rejection of the COVID-19 vaccine, recruited via snowball sampling; focus group with 4 members of *Movimento 3V* (‘Vaccines We Want the Truth’ Movement) | Elites (Government and Pharmaceutical Companies) | Vaccination | Infections - COVID-19 | Attitudes towards the vaccine |
| Leonard (2015)^128^ | United States | Cross-sectional (no date reported) | 243 parents with at least one 6-year-old child; sample representative of this population | Elites (Government, Scientists, Medical Professionals) | Vaccination | Infections - MMR | Attitudes towards the vaccine for children |
| Lessard (2022)^129^ | Canada (Quebec, Ontario and British Columbia) | Qualitative (March 2021) | 15 incarcerated adults | Elites (Scientists and Medical Professionals) | Vaccination | Infections - COVID-19 | Attitudes towards vaccine uptake |
| Leung (2023)^130^ | Australia, Norway, United Kingdom and United States | Cross-sectional (November 2021-January 2022) | 1,649 adults (Norway n = 242, UK n = 255, USA n = 915, Australia n = 237) | Elites (Scientists) | Vaccination | Infections - COVID-19 | Uptake of vaccine |
| Liu (2022)^131^ | United States (Santa Clara County, California) | Cross-sectional (December 2020-April 2021) | 509 incarcerated adults in Santa Clara County | Elites (Medical Professionals) | Vaccination | Infections - COVID-19 | Attitude towards and/or actual uptake of vaccine |
| Lupton (2023)^132^ | Australia | Qualitative (September-October 2021) | 40 Australian adults | Elites (Scientists) | Vaccination | Infections - COVID-19 | Attitude towards and actual uptake of vaccine |
| Lutrick (2022)^133^ | United States | Longitudinal (July 2020-May 2021) | 4,803 essential workers (health care personnel, frontline workers and first responders) | Elites (Government) | Vaccination | Infections - COVID-19 | Attitude towards and actual uptake of vaccine |
| Machado (2024)^134^ | Austria, Germany, Italy, Mexico and Portugal | Qualitative (August-December 2021) | 182 adults (Austria = 55, Germany = 40, Italy = 24, Mexico = 25, Portugal = 38); data from the wider Solidarity in Times of a Pandemic or SolPan(+) project | Elites (Scientists) | Vaccination | Infections - COVID-19 | Uptake of vaccine |
| Maciuszek (2023)^135^ | Poland | Longitudinal (February-August 2021) | 918 Polish adults; nationally representative | Elites (Scientists) | Vaccination | Infections - COVID-19 | Uptake of vaccine |
| Magee (2022)^136^ | United Kingdom (London) | Qualitative (May-August 2021) | 38 racial/ethnic minority patients registered at four GP practices in London | Elites (Government, Pharmaceutical Companies, Medical Professionals) | Vaccination | Infections - COVID-19 | Attitude towards and actual uptake of vaccine |
| Majee (2023)^137^ | United States (Missouri) | Qualitative (April 2021) | 21 Black adult members of a faith-based wellness program | Elites (Government, Pharmaceutical Companies, Medical Professionals) | Vaccination | Infections - COVID-19 | Attitude towards and actual uptake of vaccine |
| Martinez (2024)^138^ | United States | Repeat cross-sectional (April 2021, September 2021, April/May 2022) | Between 5,490 and 4,432 US adults (Cohort 1: 5,490, Cohort 2: 5,089, Cohort 3: 4,432); data from the 2021 U.S. Census Household Pulse Survey | Elites (Government) | Vaccination | Infections - COVID-19 | Uptake of vaccine |
| Mateo-Canedo (2023)^139^ | Spain | Cross-sectional (June 2021) | 2,120 Spanish adults (aged 18-39) | Elites (Government, Scientists and Medical Professionals) | Vaccination | Infections - COVID-19 | Uptake of vaccine |
| McLamore (2022)^140^ | United States | Cross-sectional (September and December 2020) | Study 2a: 1,672 US adults, Study 2b: 1,431 US adults ; nationally representative | Elites (Scientists) | Non-pharmaceutical infection controls | Infections - COVID-19 | Adherence to preventative measures |
| Mendenhall (2023)^141^ | United States (Northwest Iowa) | Qualitative (Summer 2020) | 86 interviews, informal conversations and public forums held in a small tourist town in northwest Iowa | Elites (Government and Healthcare System) | Non-pharmaceutical infection controls | Infections - COVID-19 | Adherence to preventative measures |
| Mesch (2015)^142^ | United States | Cross-sectional (October 2009) | 968 US adults; representative sample | Elites (Government) | Vaccination | Infections - Influenza | Uptake of vaccine |
| Mizrahi (2023)^143^ | Israel | Cross-sectional (July 2020) | 813 Israeli adults; nationally representative | Elites (Government) | Non-pharmaceutical infection controls | Infections - COVID-19 | Adherence to preventative measures |
| Mlozniak (2023)^144^ | Poland (Mazowieckie, Małopolskie, Świętokrzyskie and Podkarpackie districts) | Qualitative (November 2022-March 2023) | 50 Polish adults; recruited via snowball sampling | Elites (Government , Scientists and Pharmaceutical Companies) | Vaccination, non-pharmaceutical infection controls | Infections - COVID-19 | Attitudes and uptake towards vaccination and preventative measures |
| Morales (2022)^145^ | United States (Southwest) | Qualitative (no date) | 20 vaccine hesitant adults | Elites (Government and Healthcare System) | Vaccination | Infections - COVID-19 | Attitudes towards uptake of COVID-19 vaccine |
| Morelock (2023)^146^ | United States | Cross-sectional (September 2021) | 575 US adults | Elites (Populist Attitudes) | Vaccination | Infections - COVID-19 | Attitudes towards vaccine uptake |
| Morelock (2024)^147^ | United States | Cross-sectional (September 2021) | 575 US adults | Elites (Populist Attitudes) | Vaccination | Infections - COVID-19 | Attitudes towards vaccine uptake |
| Mugaloglu (2022)^148^ | Türkiye | Cross-sectional and Qualitative (February 2021) | 1,233 Turkish unvaccinated (against COVID-19) adults; recruited using convenience sampling | Elites (Government and Scientists) | Non-pharmaceutical infection controls | Infections - COVID-19 | Adherence to preventative measures |
| Musa (2011)^149^ | United States (Allegheny County, Pennsylvania) | Cross-sectional (June 2001-May 2002) | 1,681 Black and White adults aged 65+ who were enrolled in the Medicare Enrollment File (MEF) for Allegheny County | Elites (Medical Professionals and Healthcare System) | Vaccination, disease screening | Preventative care | Uptake of preventative health care, including influenza vaccine, prostate-specific antigen (PSA) test, mammogram and routine check-up |
| Naeim (2022)^150^ | United States | Repeat cross-sectional (October 2020, December 2020, April 2021, June/July 2021, September/October 2021) | October 2020: 14,946 adults, December 2020 = 15,229, April 2021 = 14,557; June/July 2021 = 30,857, and September/October 2021 = 33,088 ; nationally representative | Elites (Government) | Vaccination | Infections - COVID-19 | Uptake of vaccine |
| Naqvi (2022)^151^ | United Kingdom | Qualitative (July-August 2021) | 12 UK adults who were part of an ethnic minority group and expressed concerns about the COVID-19 vaccine; recruited via snowball sampling | Elites (Government) | Vaccination | Infections - COVID-19 | Uptake of vaccine |
| Newman (2024)^152^ | Canada (Greater Toronto and Hamilton Area, Ontario) | Qualitative (March-November 2021) | 40 adults who identify as a sexual and/or gender minority individual | Elites (Government and Medical Professionals) | Vaccination | Infections - COVID-19 | Uptake of vaccine |
| Nomura (2021)^153^ | Japan | Cross-sectional (February 2021) | 30,053 Japanese adults (aged 20+); nationally representative | Elites (Government and Scientists) | Vaccination | Infections - COVID-19 | Attitudes towards vaccine uptake |
| Nurmi (2022)^154^ | Finland (southern, western and central) | Qualitative (2016-2019) | 38 Finnish parents who have refused all or several vaccines for their children; recruited via purposive sampling | Elites (Government, Medical and Health Professionals, Scientists, Pharmaceutical Industry) | Vaccination | Infections - Childhood | Uptake of childhood vaccines |
| Ojikutu (2018)^155^ | United States | Cross-sectional (February-April 2016) | 855 Black adults who were HIV negative; nationally representative of the Black American population | Elites (Government and Medical Professionals) | Health care | Infections - HIV | Attitudes towards PrEP uptake |
| Oleksy (2021)^156^ | Poland | Longitudinal (May-December 2020) | T1 = 1130, T2 = 971, T3 = 818, T4 = 688 Polish adults; nationally representative | Elites (Government) | Non-pharmaceutical infection controls | Infections - COVID-19 | Adherence to preventative measures |
| Oleksy (2021)^157^ | Poland | Cross-sectional (March 2020) | Study 1: 1,046 Polish adults, nationally representative; Study 2: 1,680 Polish adults; | Elites (Government) | Non-pharmaceutical infection controls | Infections - COVID-19 | Adherence to preventative measures |
| Olsen (2020)^158^ | Denmark | Cross-sectional (March 2020) | 1,782 Danish adults | Elites (Government) | Non-pharmaceutical infection controls | Infections - COVID-19 | Adherence to preventative measures |
| Osakwe (2022)^159^ | United States (New York State) | Qualitative (February-March 2021) | 50 Black and Hispanic adults living in the New York metropolitan area | Elites (Government, Pharmaceutical Companies, Medical Professionals and the Healthcare System) | Non-pharmaceutical infection controls | Infections - COVID-19 | Attitudes towards and uptake of COVID-19 vaccine |
| Paul (2022)^160^ | United Kingdom | Longitudinal (July 2020-June 2021) | 633 UK adults; data from the COVID-19 Social Study | Elites (Government and Medical Professionals) | Vaccination | Infections - COVID-19 | Uptake of vaccine |
| Peitz (2021)^161^ | United Kingdom | Cross-sectional (May 2020) | 1,579 UK adults; nationally representative based on gender and age | Elites (Government) | Non-pharmaceutical infection controls | Infections - COVID-19 | Adherence to preventative measures |
| Pivetti (2023)^162^ | Türkiye | Cross-sectional (August-December 2021) | 570 Turkish adults | Elites (Scientists) | Vaccination | Infections - COVID-19 and Various | Attitudes towards uptake of COVID-19 vaccine and vaccines in general |
| Poduval (2023)^163^ | United Kingdom (London and surrounding areas) | Qualitative (December 2021-March 2022) | 22 individuals (14 members of the public from ethnic minority backgrounds, 8 professionals who are working with the public to increase vaccine uptake) | Elites (Government and Medical Professionals) | Vaccination | Infections - COVID-19 | Attitudes on uptake of vaccine |
| Poghosyan (2023)^164^ | United States | Cross-sectional (February-April 2021) | 3,034 community-living adult Medicare beneficiaries; data from the Medicare Current Beneficiary Survey COVID-19 Winter 2021; representative of the 16.4 million Medicare beneficiaries | Elites (Government) | Vaccination | Infections - COVID-19 | Attitudes on uptake of vaccine |
| Powell (2019)^165^ | United States (Michigan, Georgia, California, and North Carolina) | Cross-sectional (2003-2009) | 610 Black adults (aged 20+) attending barbershops and two academic institutions/events; data from the African-American Men’s Health and Social Life study | Elites (Healthcare Institutions) | Health care, disease screening | Preventative care | Uptake of preventative health care, including delays in routine checkups, blood pressure screening and cholesterol screening |
| Power (2023)^166^ | Denmark | Cross-sectional and Qualitative (April-July 2020) | 2,315 and 1,371 Danish adults (outcome dependent), 21 adult interviewees; data from the Danish Corona Diary Study | Elites (Government) | Non-pharmaceutical infection controls | Infections - COVID-19 | Adherence to preventative measures |
| Prati (2011)^167^ | Italy | Cross-sectional (February 2010) | 1,010 Italian adults | Elites (Government, Health Experts, Science, Mass Media) | Vaccination, non-pharmaceutical infection controls | Infections - H1N1 | Adherence to preventative measures and uptake of vaccine for H1N1 |
| Prusaczyk (2019)^168^ | United States | Cross-sectional (March 2017) | 296 US adults; recruited via Amazon Mechanical Turk | Out-groups (Women) | Sexual/reproductive health care | Abortion | Attitudes on abortion |
| Purvis (2022)^169^ | United States | Qualitative (September-October 2021) | 49 US adults; recruited via an online research company | Elites (Government and Medical Professionals) | Vaccination | Infections - COVID-19 | Uptake of vaccine |
| Quinn (2013)^170^ | United States | Cross-sectional (January-February 2010) | 2,042 US adults; nationally representative | Elites (Government) | Vaccination | Infections - H1N1 | Uptake of vaccine |
| Randall (2023)^171^ | United States (New York State) | Cross-sectional and Qualitative (October 2020-June 2021) | 533 adults living in the state of New York | Elites (Government and Health Professionals) | Non-pharmaceutical infection controls | Infections - COVID-19 | Uptake of a contact tracing application |
| Riad (2021)^172^ | Czechia | Cross-sectional (August-October 2021) | 362 pregnant or lactating women attending an outpatient gynaecologic clinic at the University Hospital Brno | Elites (Government, Medical Professionals and Pharmaceutical Companies) | Vaccination | Infections - COVID-19 | Uptake of vaccine |
| Riad (2021)^173^ | Czechia | Cross-sectional (April-June 2021) | 1,351 adult Czech university students | Elites (Pharmaceutical Companies and Medical Professionals) | Vaccination | Infections - COVID-19 | Attitudes towards uptake of COVID-19 vaccine |
| Rieger (2020)^174^ | Germany | Cross-sectional (March 2020) | 250 German university students at the University of Trier and state employees | Elites (Government) | Non-pharmaceutical infection controls | Infections - COVID-19 | Attitudes towards adherence to preventative measures |
| Rinato (2023)^175^ | United States | Cross-sectional (June 2022) | 220 US registered voters who voted in the 2020 election; recruited via convenience sampling | Elites (Scientists) | Non-pharmaceutical infection controls | Infections - COVID-19 | Adherence to preventative measures |
| Rivers (2024)^176^ | United States (Arizona, Florida, Texas and Utah) | Longitudinal (July 2021-October 2022) | 1,121-2,562 US children aged 6 months to 17 years enrolled in the Pediatric Research Observing Trends and Exposures in COVID-19 Timelines (PROTECT) study (data collected from children's parents, sample size is outcome dependent) | Elites (Government) | Vaccination | Infections - COVID-19 | Uptake of vaccine for children by their parents |
| Roccato (2023)^177^ | Italy | Cross-sectional (April 2021) | 1,148 Italian adults | Elites (Populist Attitudes) | Vaccination | Infections - COVID-19 | Uptake of vaccine |
| Rola (2023)^178^ | United Kingdom (London) | Cross-sectional (March-July 2022) | 620 adults with different levels of COVID-19 vaccine coverage | Elites (Medical and Health Professionals) | Vaccination | Infections - COVID-19 | Uptake of vaccine |
| Rosenfeld (2022)^179^ | United States | Cross-sectional (June 2021) | 832 unvaccinated US adults; recruited via Amazon Mechanical Turk | Elites (Scientists and Medical Professionals) | Vaccination | Infections - COVID-19 | Attitudes towards uptake and actual uptake of vaccine |
| Samore (2021)^180^ | United States | Repeat cross-sectional (May 2020, July 2020) | 906 US adults (slightly different demographic make-up in Study 1 and Study 2); recruited via Amazon Mechanical Turk | Elites (Scientists) | Non-pharmaceutical infection controls | Infections - COVID-19 | Adherence to preventative measures |
| Sanchez (2021)^181^ | United States | Cross-sectional (no date reported) | Study 1 = 415 US adults; recruited via Amazon Mechanical Turk and Turk Prime | Elites (Scientists) | Non-pharmaceutical infection controls | Infections - COVID-19 | Adherence to preventative measures |
| Santavicca (2023)^182^ | Canada (Alberta, Ontario and Quebec) | Cross-sectional (May-June 2021) | 4,905 Canadian adults; data from the Leger360 pool of registered members | Elites (Government) | Vaccination | Infections - COVID-19 | Uptake of vaccine |
| Santirocchi (2023)^183^ | Italy | Cross-sectional (March-May 2021) | 750 Italian adults | Elites (Scientists) | Vaccination | Infections - COVID-19 | Attitudes towards uptake of vaccine |
| Scandurra (2021)^184^ | Italy | Cross-sectional (October-November 2020) | 948 Italian adults | Elites (Government) | Non-pharmaceutical infection controls | Infections - COVID-19 | Adherence to preventative measures |
| Schwartz (2023)^185^ | United States (Baltimore and Howard County, Maryland; New Orleans, Louisiana; Fairfax County, Virginia; Harris County, Texas; Glenwood Springs/Garfield County, Colorado; Howard County, Maryland; and Great Plains and the Northern Plains) | Qualitative (December 2020-February 2021) | Wave 1: 232 US adults, Wave 2: 206 US adults; participants were limited to Black, Hispanic and Native Americans | Elites (Government, Pharmaceutical Industry and Healthcare System) | Vaccination | Infections - COVID-19 | Attitudes towards uptake of vaccine |
| Seale (2020)^186^ | Australia | Cross-sectional (March 2020) | 1,420 Australian adults; nationally representative | Elites (Government) | Non-pharmaceutical infection controls | Infections - COVID-19 | Adherence to preventative measures |
| Seddig (2022)^187^ | Germany | Cross-sectional (April 2021) | 5.044 German adults; nationally representative | Elites (Government and Scientists) | Vaccination | Infections - COVID-19 | Attitudes on uptake of vaccine |
| Sehgal (2023)^188^ | United States | Cross-sectional (January-May 2022) | 30,174 US parents with at least one child of COVID-19 vaccine eligible age (5–17 years); nationally representative | Elites (Government and Scientists) | Vaccination | Infections - COVID-19 | Uptake of vaccine for children by their parents |
| Sekimitsu (2022)^189^ | United States (Boston) | Qualitative (March-April, 2021) | 18 Black adults attending the Bethel AME Church in Boston | Elites (Government, Pharmaceutical Industry and Medical Professionals) | Vaccination | Infections - COVID-19 | Attitudes towards uptake of vaccine |
| Selleri (2020)^190^ | Italy (Northern region) | Cross-sectional (October 2016-March 2017) | 972 mothers of pre-school children aged 0-6; recruited via convenience sampling | Elites (Scientists, Pharmaceutical Industry and Healthcare Professionals) | Vaccination | Infections - Childhood | Attitudes towards uptake of vaccine |
| Serrano (2023)^191^ | United States (Southern) | Cross-sectional (no date reported) | 210 undergraduate students enrolled in a psychology course at a predominantly Hispanic academic institution | Elites (Scientists) | Non-pharmaceutical infection controls | Infections - COVID-19 | Adherence to preventative measures |
| Simas (2021)^192^ | Mexico (Mexico City and Toluca) | Qualitative (no date reported) | 54 pregnant women; recruited using purposive sampling | Elites (Government and Healthcare System) | Vaccination | Infections - Various | Uptake of maternal vaccinations |
| Simione (2021)^193^ | Italy | Cross-sectional (April 2020) | 374 Italian adults | Elites (Scientists) | Vaccination | Infections - COVID-19 | Uptake of vaccine |
| Smith (2022)^194^ | United States | Cross-sectional (May 2020) | 963 US adults; recruited via Amazon Mechanical Turk | Elites (Healthcare Institutions) | Non-pharmaceutical infection controls | Infections - COVID-19 | Adherence to preventative measures |
| Sowa (2021)^195^ | Poland | Cross-sectional (March 2021) | 1,000 Polish adults; data from the ‘Rise or fall? Short and long-term health and psychosocial trajectories of COVID-19 pandemics’ project; nationally representative | Elites (Government and Scientists) | Vaccination | Infections - COVID-19 | Uptake of vaccine |
| Spire (2023)^196^ | France | Longitudinal (November 2020-July 2021) | 80,906 French adults; data from the Epidemiology and Living Conditions (EpiCoV) study | Elites (Government and Scientists) | Vaccination | Infections - COVID-19 | Uptake of vaccine |
| Sprengholz (2023)^197^ | Germany | Cross-sectional (October 2021-February 2022) | 29,355 German adults; mainly under-60 population, non-representative sample | Elites (Government) | Non-pharmaceutical infection controls | Infections - COVID-19 | Adherence to preventative measures |
| Stasiuk (2021)^198^ | Poland | Longitudinal (February 2018-December 2020) | 400 Polish adults | Elites (Scientists, Medical Professionals) | Vaccination | Infections - Various; COVID-19 | Attitudes towards vaccines in general and COVID-19 vaccine specificially |
| Stoler (2022)^199^ | United States | Cross-sectional (July-August 2021) | 2,055 US adults; nationally representative | Elites (Scientists) | Vaccination | Infections - COVID-19 | Uptake of vaccine |
| Suhay (2022)^200^ | United States | Repeat cross-sectional (April- October 2020) | 29,671 US adults across all 29 weekly surveys; nationally representative, data from a weekly Axios/Ipsos Coronavirus Poll | Elites (Government and Health Professionals) | Non-pharmaceutical infection controls | Infections - COVID-19 | Adherence to preventative measures |
| Surina (2021)^201^ | Latvia | Cross-sectional (July 2020) | 2,606 Latvian adults; nationally representative | Elites (Government, Mass Media, Healthcare System) | Non-pharmaceutical infection controls | Infections - COVID-19 | Adherence to preventative measures |
| Szczepańska (2022)^202^ | Poland | Cross-sectional (November 2020, December 2020) | Study 1: 994 Polish adults and Study 2: 432 Polish adults; nationally representative | Out-groups (Women, Disabled People) | Policy changes | Abortion | Attitudes on abortion ban |
| Takamatsu (2023)^203^ | Japan | Repeat cross-sectional (September-October 2021 and September-October 2022) | 56,735 Japanese adults (28,118 in 2021 and 28,617 in 2022); nationally representative, data from the COVID-19 and Society Internet Survey (JACSIS) | Elites (Government) | Vaccination | Infections - COVID-19 | Attitude towards uptake of vaccine booster |
| Taylor (2023)^204^ | United States (Southeast Michigan) | Cross-sectional (March-April 2021) | 1,025 adult residents of southeast Michigan | Elites (Government) | Vaccination | Infections - COVID-19 | Attitudes towards uptake of COVID-19 vaccine |
| Teng (2023)^205^ | Japan | Cross-sectional (October 2021) | 3,690 adults residing in Japan (including both foreign-born nationals and Japanese nationals) | Elites (Government) | Vaccination | Infections - COVID-19 | Attitudes towards vaccine uptake |
| Thorpe (2023)^206^ | United States | Repeat cross-sectional (December 2020-March 2021) | 930 US adults, including veterans (n = 584) and non-veterans (n = 346) | Elites (Government, Scientists and Healthcare System) | Non-pharmaceutical infection controls | Infections - COVID-19 | Adherence to preventative measures, specifically mask wearing |
| Tranter (2022)^207^ | Australia (Nationwide and Tasmania) | Cross-sectional (February-June 2021 and September-October 2021) | 890 Australian adults and 1,200 Tasmanian adults; data from the 2020 Australian Survey of Social Attitudes (AuSSA), which is nationally representative, and The Tasmania Project | Elites (Government, Scientists Mass Media, Business and Industry) | Vaccination | Infections - COVID-19 | Attitudes towards and adherence to preventative measures |
| Tzeng (2023)^208^ | United States | Cross-sectional (September 2021) | 1,519 American adults; nationally representative, data from the Kaiser Family Foundation COVID-19 Vaccine Monitor | Elites (Government) | Vaccination | Infections - COVID-19 | Uptake of vaccine |
| Uddin (2021)^209^ | Japan | Cross-sectional (March 2020) | 11,342 Japanese adults; nationally representative | Elites (Government) | Non-pharmaceutical infection controls | Infections - COVID-19 | Adherence to preventative measures |
| Uluşahin (2024)^210^ | United Kingdom (England and Wales) | Cross-sectional (October 2020) | 316 English and Welsh adults | Elites (Government, Scientists and Populist Attitudes) | Non-pharmaceutical infection controls | Infections - COVID-19 | Adherence to preventative measures and uptake of contact tracing app |
| Utych (2022)^211^ | United States | Cross-sectional (February 2021) | 692 White Americans; data from the Political Systems and Attitudes Study (PSAS) | Elites (Government) | Non-pharmaceutical infection controls | Infections - COVID-19 | Attitudes to preventative measures |
| Valasek (2022)^212^ | United States (San Diego County) | Qualitative (September-November 2021) | 28 US adults who inject drugs | Elites (Government and Pharmaceutical Companies) | Vaccination | Infections - COVID-19 | Uptake of vaccine |
| Van Oost (2022)^213^ | Belgium | Cross-sectional (February and April 2021) | 8,264 unvaccinated Belgian adults; T1: 5,008 participants, T2: 3,256 participants | Elites (Government) | Vaccination | Infections - COVID-19 | Attitude towards uptake of vaccine |
| van Prooijen (2022)^214^ | The Netherlands | Cross-sectional (April 2020) | 9,033 Dutch adults; nationally representative | Elites (Government) | Non-pharmaceutical infection controls | Infections - COVID-19 | Adherence to preventative measures |
| Vauclair (2022)^215^ | Portugal | Cross-sectional (March-April 2021) | 377 Portuguese adults; recruited via convenience sampling | Elites (Government) | Vaccination | Infections - COVID-19 | Attitude towards uptake of vaccine |
| Viskupič (2022)^216^ | United States (South Dakota) | Cross-sectional (April 2021) | 3,057 registered South Dakota voters | Elites (Government and Medical Professionals) | Vaccination | Infections - COVID-19 | Uptake of vaccine |
| Viskupič (2023)^217^ | United States (South Dakota) | Longitudinal (May 2022- February 2023) | 243 full vaccinated South Dakotan adults | Elites (Government) | Vaccination | Infections - COVID-19 | Attitude towards uptake of an annual vaccine booster |
| Viskupič^218^ (2023) | United States (South Dakota) | Longitudinal (April 2021-May 2022) | 211 South Dakota registered voters aged 65 or older | Elites (Government and Medical Professionals) | Vaccination | Infections - COVID-19 | COVID-19 vaccine booster uptake |
| Volek (2024)^219^ | Czechia | Cross-sectional (January-February 2021) | 1,163 Czech GPs; representative sample | Elites (Government) | Vaccination, non-pharmaceutical infection controls | Infections - COVID-19 | Attitude towards vaccine uptake and adherence to preventative measures |
| von Wyl (2021)^220^ | Switzerland | Cross-sectional (September-October 2020) | 1,511 Swiss adults; nationally representative, data from the Swiss COVID-19 Social Monitor project | Elites (Government and Scientists) | Non-pharmaceutical infection controls | Infections - COVID-19 | Uptake of a contact tracing application |
| Wagner (2022)^221^ | United States (Detroit) | Longitudinal (October 2020-June 2021) | n = 856 US adult participants in the Detroit Metro Area Communities Study (DMACS) | Elites (Government and Medical Professionals) | Vaccination | Infections - COVID-19 | Uptake of vaccine |
| Wamba (2023)^222^ | France | Cross-sectional (May 2021) | 2,001 French adults | Elites (Government, Medical Professionals, Healthcare System and Mass Media) | Vaccination | Infections - COVID-19 | Attitudes towards and uptake of vaccine |
| Wang (2021)^223^ | Republic of Korea | Cross-sectional (August 2020) | 1,525 Korean adults; nationally representative | Elites (Government, Health Experts, Medical Professionals and Scientists) | Non-pharmaceutical infection controls | Infections - COVID-19 | Adherence to preventative measures |
| Watts (2022)^224^ | Canada (Vancouver) | Cross-sectional (February-June 2021) | 2,393 staff members at three schools in the Greater Vancouver area | Elites (Government, Health Experts, Medical Professionals) | Vaccination | Infections - COVID-19 | Attitudes towards uptake of vaccine |
| Weinstein (2022)^225^ | United States (Greater Miami Area) | Qualitative (December 2020-July 2021) | 187 adult Latino sexual minority men (LSMM) in the Greater Miami Area | Elites (Government, Science, and Public Health Experts) | Vaccination | Infections - COVID-19 | Attitudes towards uptake of COVID-19 vaccine |
| Williams (2021)^226^ | United Kingdom | Qualitative (September-November 2020) | 51 UK adults | Elites (Government) | Non-pharmaceutical infection controls | Infections - COVID-19 | Adherence to preventative measures |
| Williams (2021)^227^ | United Kingdom | Qualitative (March-July 2020) | 21 UK adults | Elites (Scientists and Health Experts) | Vaccination | Infections - COVID-19; Influenza | Attitudes towards uptake of COVID-19 vaccine booster and influenza vaccine |
| Williams (2021)^228^ | United Kingdom | Qualitative (March-April 2021) | 29 UK adults | Elites (Government and Scientists) | Vaccination | Infections - COVID-19 | Attitudes towards uptake of COVID-19 vaccine |
| Wilson (2011)^229^ | France (Provence-Alpes-Côte d’Azur and Occitanie) | Qualitative (November 2016-April 2017) | 19 general practitioners from Provence-Alpes-Côte d’Azur and Occitanie | Elites (Government) | Vaccination | Infections - Various | Attitudes towards vaccines |
| Woelfert (2020)^230^ | United Kingdom | Cross-sectional and Longitudinal (March 2020) | Cross-sectional study: 301 UK adults; Longitudinal study: 268 UK adults | Elites (Government) | Non-pharmaceutical infection controls | Infections - COVID-19 | Adherence to preventative measures |
| Wotring (2022)^231^ | United States (Indiana) | Cross-sectional (no date reported) | 561 students at a Midwestern state university who either did not intend to receive the vaccine and were undecided | Elites (Government and Health Professionals) | Vaccination | Infections - COVID-19 | Attitudes towards uptake of vaccine |
| Wright (2021)^232^ | United Kingdom | Longitudinal (March-June 2020) | 51,600 UK adults | Elites (Government and the Healthcare System) | Non-pharmaceutical infection controls | Infections - COVID-19 | Adherence to preventative measures |
| Wright (2023)^233^ | Australia (Sunshine Coast, Queensland) | Cross-sectional (2020) | 1,050 parents (aged 20-74); recruited via social media | Elites (Government) | Vaccination | Infections - Childhood | Attitudes towards vaccines |
| Yuan (2023)^234^ | United States | Cross-sectional (March 2021) | 363 US adults; nationally representative | Elites (Scientists) | Vaccination | Infections - COVID-19 | Attitudes towards uptake of vaccine |
| Zarbo (2022)^235^ | Italy | Cross-sectional (March-May 2021) | 2,015 Italian adults; nationally representative | Elites (Health Institutions and Mass Media) | Vaccination | Infections - COVID-19 | Attitudes towards uptake and actual uptake of vaccine |
| Zarzeczna (2023)^236^ | United Kingdom | Repeat cross-sectional (December 2020, February 2021 and August 2021) | Study 1: 296 UK adults, Study 2: 289 UK adults, Study 3: 456 UK adults | Elites (Scientists) | Vaccination | Infections - COVID-19 | Attitudes towards uptake and actual uptake of vaccine |
| Zatti (2022)^237^ | Italy, Germany, France, Spain, Austria, Belgium, Czech Republic, Denmark, Finland, Greece, Hungary, Netherlands, Norway, Poland, Portugal, Sweden and the United Kingdom | Cross-sectional (September 2021) | 613 adult participants from 16 European countries | Elites (Government, Health Professionals and Pharmaceutical Companies) | Vaccination | Infections - COVID-19 | Attitudes towards uptake of vaccine |
| Zidkova (2023)^238^ | Czechia | Cross-sectional (April 2021) | 1,401 Czech adults | Elites (Government) | Vaccination | Infections - COVID-19 | Uptake of vaccine |
| Zimmermann (2023)^239^ | Austria, Germany, Italy, Portugal, and Switzerland | Qualitative (Longitudinal: April 2020- November 2021) | 214 European adults: Austria (n = 61), Germany (n = 40), Italy (n = 48), Portugal (n = 38), Switzerland (n = 27) | Elites (Government and Health Authorities) | Vaccination | Infections - COVID-19 | Uptake of vaccine |
| Zysset (2021)^240^ | Switzerland | Cross-sectional (April 2020) | 2,373 students at the Zurich University of Applied Sciences (ZHAW) | Elites (Government) | Non-pharmaceutical infection controls | Infections - COVID-19 | Adherence to preventative measures |

# Appendix 4: Quality Assessment of Included Studies

|  | **Correlate score (out of 5)** | | | | | **Risk factor score**  **(out of 3)** | **Causal risk factor score (out of 7)** | **Total Score (out of 15)** |
| --- | --- | --- | --- | --- | --- | --- | --- | --- |
| **Author (Year)** | Sampling : Total population or random sampling = **1**;  Convenience or case–control sampling = **0** | Response rate: Response and retention rates ≥70% and differential attrition ≤10% = **1**;  Response rate <70% or retention rate <70% or differential attrition >10% = **0** | Study without variation in the risk factor, No analysis of change = **1**; Study with variation in the risk factor but inadequately balanced, No analysis of change = **2**;  Study without variation in the risk factor, With analysis of change = **3**; Study with variation in the risk factor but inadequately balanced, With analysis of change = **4**;  Study with variation in the risk factor and adequately balanced, No analysis of change = **5**;  Study with variation in the risk factor and adequately balanced, With analysis of change = **6**;  Randomised experiment, Targeting a risk factor = **7** | Measure of correlate: Reliability coefficient ≥.75 and reasonable face validity or criterion or convergent validity coefficient ≥.3 or more than one instrument or information source used to assess correlate = **1**; None of the above = **0** | Measure of outcome:  Reliability coefficient ≥.75 and reasonable face validity or criterion or convergent validity coefficient ≥.3 or more than one instrument or information source used to assess correlate = **1**;  None of the above = **0** | Cross-sectional data = **1**; Retrospective data = **2**; Prospective data (or study of fixed risk factor) = **3** | Study without variation in the risk factor, No analysis of change = **1**; Study with variation in the risk factor but inadequately balanced, No analysis of change = **2**;  Study without variation in the risk factor, With analysis of change = **3**; Study with variation in the risk factor but inadequately balanced, With analysis of change = **4**;  Study with variation in the risk factor and adequately balanced, No analysis of change = **5**;  Study with variation in the risk factor and adequately balanced, With analysis of change = **6**;  Randomised experiment, Targeting a risk factor = **7** |  |
| Ehrke (2023)^61^ | 0 | 0 | 0 | 1 | 1 | 3 | 6 | 11 |
| Galasso (2022)^75^ | 1 | 0 | 1 | 0 | 0 | 3 | 6 | 11 |
| Gilles (2011)^78^ | 0 | 0 | 1 | 1 | 1 | 3 | 6 | 12 |
| Kuhn (2021)^119^ | 0 | 1 | 0 | 0 | 0 | 3 | 6 | 10 |
| Latkin (2023)^123^ | 0 | 0 | 0 | 1 | 0 | 3 | 6 | 10 |
| Lutrick (2022)^133^ | 0 | 1 | 1 | 0 | 0 | 3 | 6 | 11 |
| Maciuszek (2023)^135^ | 1 | 0 | 1 | 1 | 0 | 3 | 6 | 12 |
| Oleksy (2021)^157^ | 1 | 0 | 1 | 1 | 1 | 3 | 6 | 13 |
| Paul (2022)^241^ | 0 | 0 | 1 | 0 | 0 | 3 | 4 | 8 |
| Rivers (2024)^242^ | 0 | 1 | 1 | 0 | 0 | 3 | 6 | 11 |
| Spire (2023)^243^ | 1 | 0 | 1 | 0 | 0 | 3 | 6 | 11 |
| Stasiuk (2021)^198^ | 0 | 0 | 1 | 0 | 0 | 3 | 6 | 10 |
| Viskupič (2023)^217^ | 1 | 0 | 0 | 0 | 0 | 3 | 6 | 10 |
| Viskupič (2023)^218^ | 1 | 0 | 0 | 1 | 0 | 3 | 6 | 11 |
| Wagner (2022)^221^ | 1 | 0 | 1 | 0 | 0 | 3 | 6 | 11 |
| Wright (2021)^244^ | 0 | 1 | 1 | 0 | 0 | 3 | 6 | 11 |

# Appendix 5: Summary of Findings (SoF) table using GRADE

| **Receipt of public health interventions among those who do and do not hold populist views** | | | |
| --- | --- | --- | --- |
| **Patients or population:** Adults (18+ years)  **Settings:** OECD Countries  **Intervention:** Uptake of public health interventions among those holding populist views  **Comparison:** Uptake of public health interventions amogn those not holding populist views | | | |
| **Outcomes** | **Impact** | **Number of  participants (Studies)** | **Certainty of the evidence (GRADE)*** |
| **Attitudes towards/uptake of COVID-19 vaccine** | A lack of populist attitudes—evidenced via trust in elite institutions and actors (i.e., government, politicians, scientists, medical/health professionals and mainstream media)—was generally linked to more positive attitudes towards and increased uptake of the COVID-19 vaccine. Conversely, having populist attitudes— evidence via a lack of trust in the aforementioned institutions and/or actors —was generally linked to more negative attitudes towards and lower uptake of the COVID-19 vaccine. There is also evidence to suggest that changes in levels of trust in elite institutions over time are linked to similar changes in COVID-19 vaccine attitudes and uptake. While evidence to support these findings are largely consistent across a wide array of studies and a large number of participants, the broad range of different exposure measures used to capture populist attitudes across these studies leads the certainty of evidence to be rated as low. | 104,661 (12 studies) | ⊕⊕⊖⊖ **Low** |
| **Attitudes towards/uptake of COVID-19 preventative measures** | Harboring populist (i.e., anti-elite/pro-people’s sovereignty) views and a lack of trust in elite institutions and/or actors (i.e., the government, the healthcare system, scientists and the media) were found to have a negative impact on both attitudes towards and uptake of COVID-19 preventative measures such as masking and social distancing. There is also evidence to support the role of changes in the strength of populist views and trust over time and similar changes in attitudes/uptake of preventative measures. Evidence for this outcome comes from only three studies, though they all report similar findings. Given, however, that most participants come from one study with a large sample population, the certainty of evidence has been rated as low. | 52,944 (3 studies) | ⊕⊕⊖⊖ **Low** |
| **Attitudes towards/uptake of other vaccinations** | A sense of trust in elite institutions and actors (including medical organisations, pharmaceutical companies, medical/health professionals and science) was linked to more positive attitudes towards and increased uptake of vaccination, as compared to a lack of trust in these institutions and/or actors. However, evidence is limited to two relatively small studies, and they are not looking at the same type of vaccine, so the certainty of evidence has been rated as very low. | 1,001 (2 studies) | ⊕⊖⊖⊖ **Very low** |
| * GRADE Working Group grades of evidence  **High** = This research provides a very good indication of the likely effect. The likelihood that the effect will be substantially different^†^ is low.  **Moderate** = This research provides a good indication of the likely effect. The likelihood that the effect will be substantially different^†^ is moderate.  **Low** = This research provides some indication of the likely effect. However, the likelihood that it will be substantially different^†^ is high.  **Very low** = This research does not provide a reliable indication of the likely effect. The likelihood that the effect will be substantially different^†^ is very high.  ^†^ Substantially different = a large enough difference that it might affect a decision | | | |

# References

1.	Page MJ, McKenzie JE, Bossuyt PM, et al. The PRISMA 2020 statement: An updated guideline for reporting systematic reviews. *The BMJ*. *BMJ Publishing Group*. 2021;372. doi:10.1136/bmj.n71

2.	Campbell M, McKenzie JE, Sowden A, et al. Synthesis without meta-analysis (SWiM) in systematic reviews: Reporting guideline. *The BMJ*. 2020;368. doi:10.1136/bmj.l6890

3.	Abad N, Messinger SD, Huang Q, et al. A qualitative study of behavioral and social drivers of COVID-19 vaccine confidence and uptake among unvaccinated Americans in the US April-May 2021. *PLoS One*. 2023;18(2 February). doi:10.1371/journal.pone.0281497

4.	Abadi D, Arnaldo I, Fischer A. Anxious and Angry: Emotional Responses to the COVID-19 Threat. *Front Psychol*. 2021;12. doi:10.3389/fpsyg.2021.676116

5.	Adams-Clark AA, Freyd JJ. Undergraduates’ Noncompliance with COVID-19 Regulations Is Associated with Lifetime Sexual Harassment Perpetration and Sexist Beliefs. *J Aggress Maltreat Trauma*. 2022;31(7):851-872. doi:10.1080/10926771.2022.2068395

6.	Adekola J, Fischbacher-Smith D, Okey-Adibe T, Audu J. Strategies to Build Trust and COVID-19 Vaccine Confidence and Engagement among Minority Groups in Scotland. *International Journal of Disaster Risk Science*. 2022;13(6):890-902. doi:10.1007/s13753-022-00458-7

7.	Aechtner T, Farr J. Religion, Trust, and Vaccine Hesitancy in Australia: An Examination of Two Surveys. *Journal for the Academic Study of Religion*. 2022;35(2):218-244. doi:10.1558/jasr.22476

8.	Agaku I, Adeoye C, Krow NAA, Long T. Segmentation analysis of the unvaccinated US adult population 2 years into the COVID-19 pandemic, 1 December 2021 to 7 February 2022. *Fam Med Community Health*. 2023;11(1). doi:10.1136/fmch-2022-001769

9.	Agnese G, Fabio P. “Populist Attitude and Conspiracist beliefs contribution to the overconfidence about the risk of Covid-19: implications for Preventive Health Behaviors.” Preprint posted online January 31, 2022. doi:10.1101/2022.01.30.22269992

10.	Ahluwalia SC, Edelen MO, Qureshi N, Etchegaray JM. Trust in experts, not trust in national leadership, leads to greater uptake of recommended actions during the COVID-19 pandemic. *Risk Hazards Crisis Public Policy*. 2021;12(3):283-302. doi:10.1002/rhc3.12219

11.	Ahn J, Kahlor LA. When Experts Offer Conflicting Information: A Study of Perceived Ambiguity, Information Insufficiency, Trustworthiness and Risk Information Behaviors. *Health Commun*. 2023;38(14):3276-3286.

12.	Alessandri G, Filosa L, Tisak MS, Crocetti E, Crea G, Avanzi L. Moral Disengagement and Generalized Social Trust as Mediators and Moderators of Rule-Respecting Behaviors During the COVID-19 Outbreak. *Front Psychol*. 2020;11. doi:10.3389/fpsyg.2020.02102

13.	Allen JD, De Jesus M, Mars D, Tom L, Cloutier L, Shelton RC. Decision-making about the HPV vaccine among ethnically diverse parents: Implications for health communications. *J Oncol*. Published online 2012. doi:10.1155/2012/401979

14.	Allington D, McAndrew S, Duffy B, Moxham-Hall V. Trust and experiences of National Health Service healthcare do not fully explain demographic disparities in coronavirus vaccination uptake in the UK: A cross-sectional study. *BMJ Open*. 2022;12(3). doi:10.1136/bmjopen-2021-053827

15.	Amundson CJ, Sias JJ, Frietze GA. Perceptions of COVID-19 vaccines in a predominantly Hispanic patient population from the Texas–Mexico border. *Journal of the American Pharmacists Association*. 2022;62(4):S17-S21.e2. doi:10.1016/j.japh.2021.11.003

16.	Ayalon L. Trust and compliance with covid-19 preventive behaviors during the pandemic. *Int J Environ Res Public Health*. 2021;18(5):1-10. doi:10.3390/ijerph18052643

17.	Bacon E, An L, Yang P, Hawley S, Lee Van Horn M, Resnicow K. Novel Psychosocial Correlates of COVID-19 Vaccine Hesitancy: Cross-Sectional Survey. *JMIR Form Res*. 2023;7(1). doi:10.2196/45980

18.	Baek J, Kim KH, Choi JW. Determinants of adherence to personal preventive behaviours based on the health belief model: a cross-sectional study in South Korea during the initial stage of the COVID-19 pandemic. *BMC Public Health*. 2022;22(1). doi:10.1186/s12889-022-13355-x

19.	Bajos N, Spire A, Silberzan L, et al. When Lack of Trust in the Government and in Scientists Reinforces Social Inequalities in Vaccination Against COVID-19. *Front Public Health*. 2022;10. doi:10.3389/fpubh.2022.908152

20.	Ball H, Wozniak TR. Why Do Some Americans Resist COVID-19 Prevention Behavior? An Analysis of Issue Importance, Message Fatigue, and Reactance Regarding COVID-19 Messaging. *Health Commun*. 2022;37(14):1812-1819. doi:10.1080/10410236.2021.1920717

21.	Barbieri V, Wiedermann CJ, Lombardo S, et al. Vaccine Hesitancy during the Coronavirus Pandemic in South Tyrol, Italy: Linguistic Correlates in a Representative Cross-Sectional Survey. *Vaccines (Basel)*. 2022;10(10). doi:10.3390/vaccines10101584

22.	Baumann BM, Rodriguez RM, DeLaroche AM, et al. Factors Associated With Parental Acceptance of COVID-19 Vaccination: A Multicenter Pediatric Emergency Department Cross-Sectional Analysis. *Ann Emerg Med*. 2022;80(2):130-142. doi:10.1016/j.annemergmed.2022.01.040

23.	Baumgaertner B, Carlisle JE, Justwan F. The influence of political ideology and trust on willingness to vaccinate. *PLoS One*. 2018;13(1). doi:10.1371/journal.pone.0191728

24.	Bendetson J, Swann MC, Lozano A, et al. Deepening Our Understanding of COVID-19 Vaccine Decision-Making amongst Healthcare Workers in Southwest Virginia, USA Using Exploratory and Confirmatory Factor Analysis. *Vaccines (Basel)*. 2023;11(3). doi:10.3390/vaccines11030556

25.	Bianco A, Mascaro V, Zucco R, Pavia M. Parent perspectives on childhood vaccination: How to deal with vaccine hesitancy and refusal? *Vaccine*. 2019;37(7):984-990. doi:10.1016/j.vaccine.2018.12.062

26.	Bickham SB, Francis DB. The Public’s Perceptions of Government Officials’ Communication in the Wake of the COVID-19 Pandemic. *Journal of Creative Communications*. 2021;16(2):190-202. doi:10.1177/09732586211003856

27.	Birmingham Voluntary Service Council Research, Revolving Doors, Institute for Community Research and Development (ICRD) U of W. *Vaccine Uptake amongst People with Personal Experience of Multiple Disadvantage in Birmingham: Research Findings*. 2021.

28.	Blackburn AM, Han H, Jeftić A, et al. Predictors of compliance with COVID-19 guidelines across countries: the role of social norms, moral values, trust, stress, and demographic factors. *Current Psychology*. 2024;43(19):17939-17955. doi:10.1007/s12144-023-05281-x

29.	Blanchi S, Torreggiani M, Chatrenet A, et al. COVID-19 Vaccine Hesitancy in Patients on Dialysis in Italy and France. *Kidney Int Rep*. 2021;6(11):2763-2774. doi:10.1016/j.ekir.2021.08.030

30.	Bogart LM, Ojikutu BO, Tyagi K, et al. COVID-19 Related Medical Mistrust, Health Impacts, and Potential Vaccine Hesitancy Among Black Americans Living With HIV. *J Acquir Immune Defic Syndr*. 2021;86(2):200-207.

31.	Börjesson M, Enander A. Perceptions and sociodemographic factors influencing vaccination uptake and precautionary behaviours in response to the A/H1N1 influenza in Sweden. *Scand J Public Health*. 2014;42(2):215-222. doi:10.1177/1403494813510790

32.	Boyle J, Nowak G, Kinder R, Iachan R, Dayton J. Better Understanding Adult COVID-19 Vaccination Hesitancy and Refusal: The Influence of Broader Beliefs about Vaccines. Published online 2022. doi:10.3390/ijerph

33.	Bozkurt V, Bayram Arlı N, İlhan MN, Usta EK, Budak B, Aydemir Dev M. Factors Affecting Negative Attitudes towards COVID-19 Vaccines. *Journal of Humanity and Society (insan & toplum)*. 2023;13(1):53-72. doi:10.12658/m0674

34.	Bruder M, Kunert L. The conspiracy hoax? Testing key hypotheses about the correlates of generic beliefs in conspiracy theories during the COVID-19 pandemic. *International Journal of Psychology*. 2022;57(1):43-48. doi:10.1002/ijop.12769

35.	Burton A, McKinlay A, Dawes J, et al. Understanding Barriers and Facilitators to Compliance with UK Social Distancing Guidelines during the COVID-19 Pandemic: A Qualitative Interview Study. *Behaviour Change*. 2023;40(1):30-50. doi:10.1017/bec.2021.27

36.	Butler JZ, Carson M, Rios-Fetchko F, et al. COVID-19 vaccination readiness among multiple racial and ethnic groups in the San Francisco Bay Area: A qualitative analysis. *PLoS One*. 2022;17(5 May). doi:10.1371/journal.pone.0266397

37.	Byström E, Lindstrand A, Likhite N, Butler R, Emmelin M. Parental attitudes and decision-making regarding MMR vaccination in an anthroposophic community in Sweden - A qualitative study. *Vaccine*. 2014;32(50):6752-6757. doi:10.1016/j.vaccine.2014.10.011

38.	Cáceres NA, Shirazipour CH, Herrera E, Figueiredo JC, Salvy SJ. Exploring Latino Promotores/a de Salud (Community Health Workers) knowledge, attitudes, and perceptions of COVID-19 vaccines. *SSM - Qualitative Research in Health*. 2022;2. doi:10.1016/j.ssmqr.2021.100033

39.	Capasso M, Caso D, Zimet GD. The Mediating Roles of Attitude Toward COVID-19 Vaccination, Trust in Science and Trust in Government in the Relationship Between Anti-vaccine Conspiracy Beliefs and Vaccination Intention. *Front Psychol*. 2022;13. doi:10.3389/fpsyg.2022.936917

40.	Carlson SJ, Edwards G, Blyth CC, Nattabi B, Attwell K. ‘Corona is coming’: COVID-19 vaccination perspectives and experiences amongst Culturally and Linguistically Diverse West Australians. *Health Expectations*. 2022;25(6):3062-3072. doi:10.1111/hex.13613

41.	Caserotti M, Gavaruzzi T, Girardi P, et al. Who is likely to vacillate in their COVID-19 vaccination decision? Free-riding intention and post-positive reluctance. *Prev Med (Baltim)*. 2022;154. doi:10.1016/j.ypmed.2021.106885

42.	Chayinska M, Uluğ ÖM, Ayanian AH, et al. Coronavirus conspiracy beliefs and distrust of science predict risky public health behaviours through optimistically biased risk perceptions in Ukraine, Turkey, and Germany. *Group Processes and Intergroup Relations*. 2022;25(6):1616-1634. doi:10.1177/1368430220978278

43.	Chen R, Fwu BJ, Yang TR, Chen YK, Tran QAN. To mask or not to mask: Debunking the myths of mask-wearing during COVID-19 across cultures. *PLoS One*. 2022;17(9 September). doi:10.1371/journal.pone.0270160

44.	Chen J, Chen A, Shi Y, et al. A Systematic Investigation of American Vaccination Preference via Historical Data. *Processes*. 2022;10(8). doi:10.3390/pr10081665

45.	Cherniak AD, Pirutinsky S, Rosmarin DH. Religious Beliefs, Trust In Public Figures, And Adherence to COVID-19 Health Guidelines among American Orthodox and Non-Orthodox Jews. *J Relig Health*. 2023;62(1):355-372. doi:10.1007/s10943-022-01718-y

46.	Choi Y, Fox AM. Mistrust in public health institutions is a stronger predictor of vaccine hesitancy and uptake than Trust in Trump. *Soc Sci Med*. 2022;314. doi:10.1016/j.socscimed.2022.115440

47.	Choi SI, Kim S, Jin Y, et al. Effects of Individuals’ Cultural Orientations and Trust in Government Health Communication Sources on Behavioral Intentions During a Pandemic: A Cross-Country Study. *Health Commun*. 2024;39(1):107-121. doi:10.1080/10410236.2022.2159975

48.	Choma BL, Hodson G, Sumantry D, Hanoch Y, Gummerum M. Ideological and psychological predictors of covid-19-related collective action, opinions, and health compliance across three nations. *Journal of Social and Political Psychology*. 2021;9(1):123-143. doi:10.5964/jspp.5585

49.	Cizmar A, Kalkan KO. Hostile Sexism and Abortion Attitudes in Contemporary American Public Opinion. *Politics and Gender*. 2023;41(3). doi:10.1017/S1743923X23000260

50.	Clark A, Mayben JK, Hartman C, Kallen MA, Giordano TP. Conspiracy beliefs about HIV infection are common but not associated with delayed diagnosis or adherence to care. *AIDS Patient Care STDS*. 2008;22(9):753-759. doi:10.1089/apc.2007.0249

51.	Corcoran KE, Scheitle CP, DiGregorio BD. Paranormal Beliefs, Vaccine Confidence, and COVID-19 Vaccine Uptake. *Sociology of Religion: A Quarterly Review*. 2023;84(2):111-143. doi:10.1093/socrel/srac024

52.	Cross FL, Wileden L, Buyuktur AG, et al. MICEAL Black and Latinx Perspectives on COVID-19 Vaccination: A Mixed-Methods Examination. *J Racial Ethn Health Disparities*. Published online 2023. doi:10.1007/s40615-023-01815-y

53.	Cunningham-Erves J, Forbes L, Ivankova N, Mayo-Gamble T, Kelly-Taylor K, Deakings J. Black mother’s intention to vaccinate daughters against HPV: A mixed methods approach to identify opportunities for targeted communication. *Gynecol Oncol*. 2018;149(3):506-512. doi:10.1016/j.ygyno.2018.03.047

54.	Dell’Imperio SG, Aboul-Hassan D, Batchelor R, et al. Vaccine perceptions among Black adults with long COVID. *Ethn Health*. 2023;28(6):853-873. doi:10.1080/13557858.2023.2191914

55.	Denford S, Mowbray F, Towler L, et al. Exploration of attitudes regarding uptake of COVID-19 vaccines among vaccine hesitant adults in the UK: a qualitative analysis. *BMC Infect Dis*. 2022;22(1). doi:10.1186/s12879-022-07380-9

56.	Dennis A, Robin C, Jones L, Carter H. Exploring Vaccine Hesitancy in Care Home Employees in North West England: A Qualitative Study. Preprint posted online August 30, 2021. doi:10.1101/2021.08.20.21262101

57.	Di Napoli F, Mari S, Đorđević JM, Kljajić D. Examining the influence of information-related factors on vaccination intentions via confidence: Insights from adult samples in Italy and Serbia during the COVID-19 pandemic. *Soc Personal Psychol Compass*. 2024;18(2). doi:10.1111/spc3.12929

58.	Dohle S, Wingen T, Schreiber M. Acceptance and Adoption of Protective Measures during the COVID-19 Pandemic: The Role of Trust in Politics and Trust in Science. *Social Psychological Bulletin*. 2020;15(4). doi:10.32872/spb.4315

59.	Dupuis M, Chhor K, Ly N. Misinformation and Disinformation in the Era of COVID-19: The Role of Primary Information Sources and the Development of Attitudes Toward Vaccination. In: *SIGITE 2021 - Proceedings of the 22nd Annual Conference on Information Technology Education*. Association for Computing Machinery, Inc; 2021:105-110. doi:10.1145/3450329.3476866

60.	Ebrahimi O V., Johnson MS, Ebling S, et al. Risk, Trust, and Flawed Assumptions: Vaccine Hesitancy During the COVID-19 Pandemic. *Front Public Health*. 2021;9. doi:10.3389/fpubh.2021.700213

61.	Ehrke F, Grommisch G, Busch EP, Kaczmarek MC. Populist Attitudes Predict Compliance-Related Attitudes and Behaviors During the COVID-19 Pandemic Via Trust in Institutions. *Soc Psychol*. 2023;54(1-2):78-94. doi:10.1027/1864-9335/a000500

62.	Enders AM, Uscinski J, Klofstad C, Stoler J. On the relationship between conspiracy theory beliefs, misinformation, and vaccine hesitancy. *PLoS One*. 2022;17(10 October). doi:10.1371/journal.pone.0276082

63.	Eraso Y, Hills S. Intentional and unintentional non-adherence to social distancing measures during COVID-19: A mixed-methods analysis. *PLoS One*. 2021;16(8 August). doi:10.1371/journal.pone.0256495

64.	Eshel Y, Kimhi S, Marciano H, Adini B. Conspiracy claims and secret intentions as predictors of psychological coping and vaccine uptake during the COVID-19 pandemic. *J Psychiatr Res*. 2022;151:311-318. doi:10.1016/j.jpsychires.2022.04.042

65.	Eshel Y, Kimhi S, Marciano H, Adini B. Belonging to Socially Excluded Groups as a Predictor of Vaccine Hesitancy and Rejection. *Front Public Health*. 2022;9. doi:10.3389/fpubh.2021.823795

66.	Farhart CE, Douglas-Durham E, Lunz Trujillo K, Vitriol JA. Vax attacks: How conspiracy theory belief undermines vaccine support. In: *Progress in Molecular Biology and Translational Science*. Vol 188. Elsevier B.V.; 2022:135-169. doi:10.1016/bs.pmbts.2021.11.001

67.	Fattorini E. Rediscussing the Primacy of Scientific Expertise: A Case Study on Vaccine Hesitant Parents in Trentino. *Tecnoscienza*. 2023;14(1):77-103. doi:10.6092/issn.2038-3460/17606

68.	Fernández-Penny FE, Jolkovsky EL, Shofer FS, et al. COVID-19 vaccine hesitancy among patients in two urban emergency departments. *Academic Emergency Medicine*. 2021;28(10):1100-1107. doi:10.1111/acem.14376

69.	Fischer EP, McSweeney JC, Wright P, et al. Overcoming Barriers to Sustained Engagement in Mental Health Care: Perspectives of Rural Veterans and Providers. *J Rural Health*. 2016;32(4):429-438. doi:10.1111/jrh.12203

70.	Fleming PJ, Patterson TL, Chavarin C V., Semple SJ, Magis-Rodriguez C, Pitpitan E V. Behavioral and Psychosocial Correlates of HIV Testing Among Male Clients of Female Sex Workers in Tijuana, Mexico. *AIDS Behav*. 2017;21(8):2322-2331. doi:10.1007/s10461-016-1531-6

71.	Ford CL, Wallace SP, Newman PA, Lee SJ, Cunningham WE. Belief in AIDS-related conspiracy theories and mistrust in the government: Eelationship with HIV testing among at-risk older adults. *Gerontologist*. 2013;53(6):973-984. doi:10.1093/geront/gns192

72.	Frew PM, Painter JE, Hixson B, et al. Factors mediating seasonal and influenza A (H1N1) vaccine acceptance among ethnically diverse populations in the urban south. *Vaccine*. 2012;30(28):4200-4208. doi:10.1016/j.vaccine.2012.04.053

73.	Fridman I, Lucas N, Henke D, Zigler CK. Association between public knowledge about COVID-19, trust in information sources, and adherence to social distancing: Cross-sectional survey. *JMIR Public Health Surveill*. 2020;6(3). doi:10.2196/22060

74.	Frietze G, Padilla M, Cordero J, Gosselink K, Moya E. Human Papillomavirus Vaccine Acceptance (HPV-VA) and Vaccine Uptake (HPV-VU): assessing the impact of theory, culture, and trusted sources of information in a Hispanic community. *BMC Public Health*. 2023;23(1). doi:10.1186/s12889-023-16628-1

75.	Galasso V, Pons V, Profeta P, Becher M, Brouard S, Foucault M. *NBER Working Paper Series: From Anti-Vax Intentions to Vaccination: Panel and Experimental Evidence from Nine Countries*. 2022. http://www.nber.org/papers/w29741

76.	Geana M V., Anderson S, Ramaswamy M. COVID-19 vaccine hesitancy among women leaving jails: A qualitative study. *Public Health Nurs*. 2021;38(5):892-896. doi:10.1111/phn.12922

77.	Gehlbach D, Vázquez E, Ortiz G, et al. Perceptions of the Coronavirus and COVID-19 testing and vaccination in Latinx and Indigenous Mexican immigrant communities in the Eastern Coachella Valley. *BMC Public Health*. 2022;22(1). doi:10.1186/s12889-022-13375-7

78.	Gilles I, Bangerter A, Clémence A, et al. Trust in medical organizations predicts pandemic (H1N1) 2009 vaccination behavior and perceived efficacy of protection measures in the Swiss public. *Eur J Epidemiol*. 2011;26(3):203-210. doi:10.1007/s10654-011-9577-2

79.	Goldfinch S, Taplin R. A cross-sectional international study shows confidence in public health scientists predicts use of COVID-19 non-pharmaceutical interventions. *BMC Public Health*. 2022;22(1). doi:10.1186/s12889-022-13074-3

80.	Goodwin R, Ben-Ezra M, Takahashi M, et al. Psychological factors underpinning vaccine willingness in Israel, Japan and Hungary. *Sci Rep*. 2022;12(1). doi:10.1038/s41598-021-03986-2

81.	Goren T, Vashdi DR, Beeri I. Count on trust: the indirect effect of trust in government on policy compliance with health behavior instructions. *Policy Sci*. 2022;55(4):593-630. doi:10.1007/s11077-022-09481-3

82.	Gothreau C, Arceneaux K, Friesen A. Hostile, Benevolent, Implicit: How Different Shades of Sexism Impact Gendered Policy Attitudes. *Front Polit Sci*. 2022;4. doi:10.3389/fpos.2022.817309

83.	Gray CA, Lesser G, Guo Y, et al. COVID-19 Vaccination Intention and Factors Associated with Hesitance and Resistance in the Deep South: Montgomery, Alabama. *Trop Med Infect Dis*. 2022;7(11). doi:10.3390/tropicalmed7110331

84.	Grežo M, Adamus M. Light and Dark core of personality and the adherence to COVID-19 containment measures: The roles of motivation and trust in government. *Acta Psychol (Amst)*. 2022;223. doi:10.1016/j.actpsy.2021.103483

85.	Guazzini A, Fiorenza M, Panerai G, Duradoni M. What went wrong? Predictors of contact tracing adoption in Italy during COVID-19 pandemic. *Future Internet*. 2021;13(11). doi:10.3390/fi13110286

86.	Guillon M, Kergall P. Attitudes and opinions on quarantine and support for a contact-tracing application in France during the COVID-19 outbreak. *Public Health*. 2020;188:21-31. doi:10.1016/j.puhe.2020.08.026

87.	Guillon M. Digital contact-tracing in France: Uptake by COVID-19 risk factor and by exposure risk. *Journal of Public Health (United Kingdom)*. 2022;44(3):E366-E375. doi:10.1093/pubmed/fdab349

88.	Hafner-Fink M, Uhan S. Life and Attitudes of Slovenians during the COVID-19 Pandemic: The Problem of Trust. *Int J Sociol*. 2021;51(1):76-85. doi:10.1080/00207659.2020.1837480

89.	Hamada Y, Nagamatsu M, Sato T. Factors Influencing Maternal Acceptance of Human Papillomavirus Vaccination for Their School-Aged Daughters in Fukuoka Prefecture, Japan. *Br J Med Med Res*. 2015;7(5):341-354. doi:10.9734/bjmmr/2015/15917

90.	Hartman TK, Marshall M, Stocks TVA, et al. Different Conspiracy Theories Have Different Psychological and Social Determinants: Comparison of Three Theories About the Origins of the COVID-19 Virus in a Representative Sample of the UK Population. *Front Polit Sci*. 2021;3. doi:10.3389/fpos.2021.642510

91.	Hill PL, Allemand M, Burrow AL. Trust in purpose, or trust and purpose?: Institutional trust influences the association between sense of purpose and COVID-19 vaccination. *J Psychosom Res*. 2023;165. doi:10.1016/j.jpsychores.2022.111119

92.	Hills S, Eraso Y. Factors associated with non-adherence to social distancing rules during the COVID-19 pandemic: a logistic regression analysis. *BMC Public Health*. 2021;21(1). doi:10.1186/s12889-021-10379-7

93.	Hong C, Holloway IW, Bednarczyk R, Javanbakht M, Shoptaw S, Gorbach PM. High Vaccine Confidence Is Associated with COVID-19 Vaccine Uptake in Gay, Bisexual, and Other Men Who Have Sex with Men Who Use Substances. *LGBT Health*. 2023;10(6):480-485. doi:10.1089/lgbt.2022.0255

94.	Hori D, Kaneda Y, Ozaki A, Tabuchi T. Sexual orientation was associated with intention to be vaccinated with a smallpox vaccine against mpox: A cross-sectional preliminary survey in Japan. *Vaccine*. 2023;41(27):3954-3959. doi:10.1016/j.vaccine.2023.05.050

95.	Hosokawa Y, Okawa S, Hori A, et al. The Prevalence of COVID-19 Vaccination and Vaccine Hesitancy in Pregnant Women: An Internet-based Cross-sectional Study in Japan. *J Epidemiol*. 2022;32(4):188-194. doi:10.2188/jea.JE20210458

96.	Huang W, Shealey J, Sanchez K, et al. COVID-19 Vaccine Attitudes among a Majority Black Sample in the Southern US: Public Health Implications from a Qualitative Study. Preprint posted online 2022. doi:10.21203/rs.3.rs-1918432/v1

97.	Huang HY, Gerend MA. The role of trust, vaccine information exposure, and Health Belief Model variables in COVID-19 vaccination intentions: Evidence from an HBCU sample. *J Health Psychol*. 2024;29(6):621-632. doi:10.1177/13591053241227388

98.	Hubble MW, Renkiewicz GK, Hunter S, Kearns RD. Predictors of COVID-19 Vaccination among EMS Personnel. *Western Journal of Emergency Medicine*. 2022;23(4):570-578. doi:10.5811/westjem.2022.4.54926

99.	Jach Ł. The scientistic worldview and its relationships with fear of COVID, conspiracy beliefs, preventive behaviors, and attitudes towards vaccines during the COVID-19 pandemic in a Polish sample. *Current Issues in Personality Psychology*. 2023;11(1):11-28. doi:10.5114/cipp.2021.111633

100.	Jamison AM, Quinn SC, Freimuth VS. “You don’t trust a government vaccine”: Narratives of institutional trust and influenza vaccination among African American and white adults. *Soc Sci Med*. 2019;221:87-94. doi:10.1016/j.socscimed.2018.12.020

101.	Jennings W, Stoker G, Bunting H, et al. Lack of trust, conspiracy beliefs, and social media use predict COVID-19 vaccine hesitancy. *Vaccines (Basel)*. 2021;9(6). doi:10.3390/vaccines9060593

102.	Jennings W, Valgarðsson V, McKay L, Stoker G, Mello E, Baniamin HM. Trust and vaccine hesitancy during the COVID-19 pandemic: A cross-national analysis. *Vaccine X*. 2023;14. doi:10.1016/j.jvacx.2023.100299

103.	Jeong JS, Kim SY. Risk Perception and Preventive Behavior During the COVID-19 Pandemic : Testing the Effects of Government Trust and Information Behaviors. *Health Commun*. 2024;39(2):376-387. doi:10.1080/10410236.2023.2166698

104.	Jiang L, Bettac EL, Lee HJ, Probst TM. In Whom Do We Trust? A Multifoci Person-Centered Perspective on Institutional Trust during COVID-19. *Int J Environ Res Public Health*. 2022;19(3). doi:10.3390/ijerph19031815

105.	Jimenez ME, Rivera-Núñez Z, Crabtree BF, et al. Black and Latinx Community Perspectives on COVID-19 Mitigation Behaviors, Testing, and Vaccines. *JAMA Netw Open*. 2021;4(7). doi:10.1001/jamanetworkopen.2021.17074

106.	Johnson BB. Factors in intention to get the COVID-19 vaccine change over time: Evidence from a two-wave U.S. study. *Health Risk Soc*. 2023;25(3-4):151-179. doi:10.1080/13698575.2023.2173727

107.	Jones D, McDermott ML. *Partisanship and the Politics of COVID Vaccine Hesitancy*. 2021. https://academicworks.cuny.edu/bb_pubs/1250Discoveradditionalworksat:https://academicworks.cuny.edu

108.	Jung YJ, Gagneux-Brunon A, Bonneton M, et al. Factors associated with COVID-19 vaccine uptake among French population aged 65 years and older: results from a national online survey. *BMC Geriatr*. 2022;22(1). doi:10.1186/s12877-022-03338-3

109.	Karaarslan-Semiz G, Çakır-Yıldırım B, Tuncay-Yüksel B, Ozturk N, Irmak M. What can be learned from pre-service teachers’ intentions to vaccinate against COVID-19 and relevant factors for future crises? A cross-sectional survey research. *Journal of Turkish Science Education*. 2023;20(3):567-586. doi:10.36681/tused.2023.032

110.	Kim JW, Lee J, Dai Y. Misinformation and the Paradox of Trust during the covid-19 pandemic in the U.S.: pathways to Risk perception and compliance behaviors. *J Risk Res*. 2023;26(5):469-484. doi:10.1080/13669877.2023.2176910

111.	Kim S, Choi SI, Valentini C, Badham M, Jin Y. How Motivation to Reduce Uncertainty Predicts COVID-19 Behavioral Responses: Strategic Health Communication Insights for Managing an Ongoing Pandemic. *American Behavioral Scientist*. Published online 2023. doi:10.1177/00027642231164051

112.	Kohler S, Koinig I. The Effect of Science-Related Populism on Vaccination Attitudes and Decisions. *J Behav Med*. 2023;46(1-2):229-238. doi:10.1007/s10865-022-00333-2

113.	Kosic A, Kana Kenfack CS, Dionisi E. The relationship between populism and attitudes on vaccine against COVID-19: Trust in institutions as a moderation factor. *Analyses of Social Issues and Public Policy*. 2024;24(1):150-169. doi:10.1111/asap.12378

114.	Koskan AM, LoCoco IE, Daniel CL, Teeter BS. Rural Americans’ COVID-19 Vaccine Perceptions and Willingness to Vaccinate against COVID-19 with Their Community Pharmacists: An Exploratory Study. *Vaccines (Basel)*. 2023;11(1). doi:10.3390/vaccines11010171

115.	Kossowska M, Szwed P, Czarnek G. Ideology shapes trust in scientists and attitudes towards vaccines during the COVID-19 pandemic. *Group Processes and Intergroup Relations*. 2021;24(5):720-737. doi:10.1177/13684302211001946

116.	Kowalski E, Stengel A, Schneider A, Goebel-Stengel M, Zipfel S, Graf J. Article How to Motivate SARS-CoV-2 Convalescents to Receive a Booster Vaccination? Influence on Vaccination Willingness. *Vaccines (Basel)*. 2022;10(3). doi:10.3390/vaccines10030455

117.	Krastev S, Krajden O, Vang ZM, et al. Institutional trust is a distinct construct related to vaccine hesitancy and refusal. *BMC Public Health*. 2023;23(1). doi:10.1186/s12889-023-17345-5

118.	Krupenkin M. Does Partisanship Affect Compliance with Government Recommendations? *Polit Behav*. 2021;43(1):451-472. doi:10.1007/s11109-020-09613-6

119.	Kuhn R, Henwood B, Lawton A, et al. COVID-19 vaccine access and attitudes among people experiencing homelessness from pilot mobile phone survey in Los Angeles, CA. Preprint posted online March 26, 2021. doi:10.1101/2021.03.23.21254146

120.	Kuroki M, Yamamoto K, Goldfinch S. Factors Influencing the Adoption of Voluntary Nonpharmaceutical Interventions to Control COVID-19 in Japan: Cross-sectional Study. *JMIR Form Res*. 2022;6(8). doi:10.2196/34268

121.	Ladini R, Maggini N. The role of party preferences in explaining acceptance of freedom restrictions in a pandemic context: the Italian case. *Qual Quant*. 2023;57:99-123. doi:10.1007/s11135-022-01436-3

122.	Lalot F, Abrams D, Jessop C, Curtice J. Compliance in crisis: Concern, trust and distrustful complacency in the COVID-19 pandemic. *Soc Personal Psychol Compass*. 2023;17(7). doi:10.1111/spc3.12752

123.	Latkin C, Dayton L, Miller J, et al. Trusted information sources in the early months of the COVID-19 pandemic predict vaccination uptake over one year later. *Vaccine*. 2023;41(2):573-580. doi:10.1016/j.vaccine.2022.11.076

124.	Lee C, Whetten K, Omer S, Pan W, Salmon D. Hurdles to herd immunity: Distrust of government and vaccine refusal in the US, 2002–2003. *Vaccine*. 2016;34(34):3972-3978. doi:10.1016/j.vaccine.2016.06.048

125.	Lee T, Santillana M, Lacasa-Mas I, Ashe I. Antecedents and consequences of Americans’ COVID-19 conspiracy beliefs: a focus on support for trump, populism, institutional trust, media consumption, and mask-wearing attitudes. *Int J Public Opin Res*. 2022;34(3). doi:10.1093/ijpor/edac019

126.	Lehr M. *An Analysis of Trust and Trustworthiness within Political and Banking Institutions*. George Mason University; 2021.

127.	Lello E, Bertuzzi N, Pedroni M, Raffini L. Vaccine hesitancy and refusal during the Covid-19 pandemic in Italy: Individualistic claims or repoliticisation? *Partecipazione e Conflitto*. 2022;15(3):672-696. doi:10.1285/i20356609v15i3p672

128.	Leonard W. *Parental Confidence in U.S. Government and Medical Authorities, Measles (Rubeloa) Knowledge, and MMR Vaccine Compliance*. Walden University; 2015. https://scholarworks.waldenu.edu/dissertations

129.	Lessard D, Ortiz-Paredes D, Park H, et al. Barriers and facilitators to COVID-19 vaccine acceptability among people incarcerated in Canadian federal prisons: A qualitative study. *Vaccine X*. 2022;10. doi:10.1016/j.jvacx.2022.100150

130.	Leung J, Price D, McClure-Thomas C, et al. Motivation and Hesitancies in Obtaining the COVID-19 Vaccine—A Cross-Sectional Study in Norway, USA, UK, and Australia. *Vaccines (Basel)*. 2023;11(6). doi:10.3390/vaccines11061086

131.	Liu YE, Oto J, Will J, et al. Factors associated with COVID-19 vaccine acceptance and hesitancy among residents of Northern California jails. *Prev Med Rep*. 2022;27. doi:10.1016/j.pmedr.2022.101771

132.	Lupton D. Attitudes to COVID-19 Vaccines among Australians during the Delta Variant Wave: A Qualitative Interview Study. *Health Promot Int*. 2023;38(1). doi:10.1093/heapro/daac192

133.	Lutrick K, Groom H, Fowlkes AL, et al. COVID-19 vaccine perceptions and uptake in a national prospective cohort of essential workers. *Vaccine*. 2022;40(3):494-502. doi:10.1016/j.vaccine.2021.11.094

134.	Machado H, de Freitas C, Fiske A, et al. Performing publics of science in the COVID-19 pandemic: A qualitative study in Austria, Bolivia, Germany, Italy, Mexico, and Portugal. *Public Understanding of Science*. 2024;33(4):466-482. doi:10.1177/09636625231220219

135.	Maciuszek J, Polak M, Stasiuk K, Rosiński J. Declared Intention to Vaccinate against COVID-19 and Actual Vaccination—The Role of Trust in Science, Conspiratorial Thinking and Religiosity. *Vaccines (Basel)*. 2023;11(2). doi:10.3390/vaccines11020262

136.	Magee L, Knights F, McKechnie DGJ, Al-Bedaery R, Razai MS. Facilitators and barriers to COVID-19 vaccination uptake among ethnic minorities: A qualitative study in primary care. *PLoS One*. 2022;17(7 July). doi:10.1371/journal.pone.0270504

137.	Majee W, Anakwe A, Onyeaka K, Harvey IS. The Past Is so Present: Understanding COVID-19 Vaccine Hesitancy Among African American Adults Using Qualitative Data. *J Racial Ethn Health Disparities*. 2023;10(1):462-474. doi:10.1007/s40615-022-01236-3

138.	Martinez DL. *Understanding Attitudes, Subjective Norms, and Intent of COVID-19 Vaccine Hesitancy and Refusal Among African Americans in the United States Using the Theory of Reasoned Action*. The University of Alabama at Birmingham; 2023. https://digitalcommons.library.uab.edu/etd-collection/392

139.	Mateo-Canedo C, Sanabria-Mazo JP, Comendador L, et al. Predictive factors of hesitancy to vaccination against SARS-CoV-2 virus in young adults in Spain: Results from the PSY-COVID study. *Vaccine X*. 2023;14. doi:10.1016/j.jvacx.2023.100301

140.	McLamore Q, Syropoulos S, Leidner B, et al. Trust in scientific information mediates associations between conservatism and coronavirus responses in the U.S., but few other nations. *Sci Rep*. 2022;12(1). doi:10.1038/s41598-022-07508-6

141.	Mendenhall E. Trust, individualism, and the logics of care in middle America during the first year of the COVID-19 pandemic. *Transcult Psychiatry*. Published online 2023. doi:10.1177/13634615231213839

142.	Mesch GS, Schwirian KP. Social and political determinants of vaccine hesitancy: Lessons learned from the H1N1 pandemic of 2009-2010. *Am J Infect Control*. 2015;43(11):1161-1165. doi:10.1016/j.ajic.2015.06.031

143.	Mizrahi S, Cohen N, Vigoda-Gadot E, Krup DN. Compliance with government policies during emergencies: Trust, participation and protective actions. *Governance*. 2023;36(4):1083-1102. doi:10.1111/gove.12716

144.	Młoźniak I, Zwierczyk U, Rzepecka E, Kobryn M, Wilk M, Duplaga M. Manifestation of Health Denialism in Attitudes toward COVID-19 Vaccination: A Qualitative Study. *Vaccines (Basel)*. 2023;11(12). doi:10.3390/vaccines11121822

145.	Morales GI, Lee S, Bradford A, De Camp A, Tandoc EC. Exploring vaccine hesitancy determinants during the COVID-19 pandemic: An in-depth interview study. *SSM - Qualitative Research in Health*. 2022;2. doi:10.1016/j.ssmqr.2022.100045

146.	Morelock J, Michelotti A, Uyen LHM. Vaccine Hesitancy and Attitudes Toward Elite Knowledge in the United States During COVID-19. *Crit Sociol (Eugene)*. 2024;50(2):317-334. doi:10.1177/08969205231180267

147.	Morelock J, Oliveira A, Ly HMU, Ward CL. Populism, moral foundations, and vaccine hesitancy during COVID-19. *Social Theory and Health*. 2024;22(1):71-87. doi:10.1057/s41285-023-00201-2

148.	Muğaloğlu EZ, Kaymaz Z, Mısır ME, Laçin-Şimşek C. Exploring the Role of Trust in Scientists to Explain Health-Related Behaviors in Response to the COVID-19 Pandemic. *Sci Educ (Dordr)*. 2022;31(5):1281-1309. doi:10.1007/s11191-022-00323-5

149.	Musa D, Schulz R, Harris R, Silverman M, Thomas SB. Trust in the health care system and the use of preventive health services by older black and white adults. *Am J Public Health*. 2009;99(7):1293-1299. doi:10.2105/AJPH.2007.123927

150.	Naeim A, Guerin RJ, Baxter-King R, et al. Strategies to increase the intention to get vaccinated against COVID-19: Findings from a nationally representative survey of US adults, October 2020 to October 2021. *Vaccine*. 2022;40(52):7571-7578. doi:10.1016/j.vaccine.2022.09.024

151.	Naqvi M, Li L, Woodrow M, Yadav P, Kostkova P. Understanding COVID-19 Vaccine Hesitancy in Ethnic Minorities Groups in the UK. *Front Public Health*. 2022;10. doi:10.3389/fpubh.2022.917242

152.	Newman PA, Dinh DA, Massaquoi N, et al. “Going vaccine hunting”: Multilevel influences on COVID-19 vaccination among racialized sexual and gender minority adults—a qualitative study. *Hum Vaccin Immunother*. 2024;20(1). doi:10.1080/21645515.2023.2301189

153.	Nomura S, Eguchi A, Yoneoka D, et al. Reasons for being unsure or unwilling regarding intention to take COVID-19 vaccine among Japanese people: A large cross-sectional national survey. *Lancet Reg Health West Pac*. 2021;14. doi:10.1016/j.lanwpc.2021.100223

154.	Nurmi J, Harman B. Why do parents refuse childhood vaccination? Reasons reported in Finland. *Scand J Public Health*. 2022;50(4):490-496. doi:10.1177/14034948211004323

155.	Ojikutu BO, Bogart LM, Higgins-Biddle M, et al. Facilitators and Barriers to Pre-Exposure Prophylaxis (PrEP) Use Among Black Individuals in the United States: Results from the National Survey on HIV in the Black Community (NSHBC). *AIDS Behav*. 2018;22(11):3576-3587. doi:10.1007/s10461-018-2067-8

156.	Oleksy T, Wnuk A, Gambin M, Łyś A. Dynamic relationships between different types of conspiracy theories about COVID-19 and protective behaviour: A four-wave panel study in Poland. *Soc Sci Med*. 2021;280. doi:10.1016/j.socscimed.2021.114028

157.	Oleksy T, Wnuk A, Maison D, Łyś A. Content matters. Different predictors and social consequences of general and government-related conspiracy theories on COVID-19. *Pers Individ Dif*. 2021;168. doi:10.1016/j.paid.2020.110289

158.	Olsen AL, Hjorth F. *Willingness to Distance in the COVID-19 Pandemic*. 2020.

159.	Osakwe ZT, Osborne JC, Osakwe N, Stefancic A. Facilitators of COVID-19 vaccine acceptance among Black and Hispanic individuals in New York: A qualitative study. *Am J Infect Control*. 2022;50(3):268-272. doi:10.1016/j.ajic.2021.11.004

160.	Paul E, Fancourt D, Razai M. Racial discrimination, low trust in the health system and COVID-19 vaccine uptake: a longitudinal observational study of 633 UK adults from ethnic minority groups. *J R Soc Med*. 2022;115(11):439-447. doi:10.1177/01410768221095241

161.	Peitz L, Lalot F, Douglas K, Sutton R, Abrams D. COVID-19 conspiracy theories and compliance with governmental restrictions: The mediating roles of anger, anxiety, and hope. *Journal of Pacific Rim Psychology*. 2021;15. doi:10.1177/18344909211046646

162.	Pivetti M, Paleari FG, Ertan I, Di Battista S, Ulukök E. COVID-19 conspiracy beliefs and vaccinations: A conceptual replication study in Turkey. *Journal of Pacific Rim Psychology*. *SAGE Publications Ltd*. 2023;17. doi:10.1177/18344909231170097

163.	Poduval S, Kamal A, Martin S, Islam A, Kaviraj C, Gill P. Beyond Information Provision: Analysis of the Roles of Structure and Agency in COVID-19 Vaccine Confidence in Ethnic Minority Communities. *Int J Environ Res Public Health*. 2023;20(21). doi:10.3390/ijerph20217008

164.	Poghosyan H, Ni Z, Vlahov D, Nelson LR, Nam S. COVID-19 Vaccine Hesitancy Among Medicare Beneficiaries with and Without Cancer History: A US Population-based Study. *J Community Health*. 2023;48(2):315-324. doi:10.1007/s10900-022-01174-5

165.	Powell W, Richmond J, Mohottige D, Yen I, Joslyn A, Corbie-Smith G. Medical Mistrust, Racism, and Delays in Preventive Health Screening Among African-American Men. *Behavioral Medicine*. 2019;45(2):102-117. doi:10.1080/08964289.2019.1585327

166.	Power SA, Schaeffer M, Heisig JP, Udsen R, Morton T. Why trust? A mixed-method investigation of the origins and meaning of trust during the COVID-19 lockdown in Denmark. *British Journal of Social Psychology*. 2023;62(3):1376-1394. doi:10.1111/bjso.12637

167.	Prati G, Pietrantoni L, Zani B. Compliance with recommendations for pandemic influenza H1N1 2009: The role of trust and personal beliefs. *Health Educ Res*. 2011;26(5):761-769. doi:10.1093/her/cyr035

168.	Prusaczyk E, Hodson G. Re-examining left-right differences in abortion opposition: The roles of sexism and shared reality. *TPM Test Psychom Methodol Appl Psychol*. 2019;26(3):431-445. doi:10.4473/TPM26.3.8

169.	Purvis RS, Moore R, Willis DE, Hallgren E, McElfish PA. Factors influencing COVID-19 vaccine decision-making among hesitant adopters in the United States. *Hum Vaccin Immunother*. 2022;18(6). doi:10.1080/21645515.2022.2114701

170.	Quinn SC, Parmer J, Freimuth VS, Hilyard KM, Musa D, Kim KH. Exploring communication, trust in government, and vaccination intention later in the 2009 H1N1 pandemic: Results of a national survey. *Biosecurity and Bioterrorism*. 2013;11(2):96-106. doi:10.1089/bsp.2012.0048

171.	Randall JG, Dalal DK, Dowden A. Factors associated with contact tracing compliance among communities of color in the first year of the COVID-19 pandemic. *Soc Sci Med*. 2023;322. doi:10.1016/j.socscimed.2023.115814

172.	Riad A, Jouzová A, Üstün B, et al. Covid-19 vaccine acceptance of pregnant and lactating women (Plw) in czechia: An analytical cross-sectional study. *Int J Environ Res Public Health*. 2021;18(24). doi:10.3390/ijerph182413373

173.	Riad A, Pokorná A, Antalová N, et al. Prevalence and drivers of COVID-19 vaccine hesitancy among Czech university students: National cross-sectional study. *Vaccines (Basel)*. 2021;9(9). doi:10.3390/vaccines9090948

174.	Rieger MO. What Makes Young People Think Positively About Social Distancing During the Corona Crisis in Germany? *Frontiers in Sociology*. 2020;5. doi:10.3389/fsoc.2020.00061

175.	Rinato BL. *Influence of Conspiracy Mindset, Trust in Science, and Political Affiliation on COVID-19 Reactance*. Walden University; 2023. https://scholarworks.waldenu.edu/dissertations

176.	Rivers P, Porter C, LeClair LB, et al. Longitudinal parental perception of COVID-19 vaccines for children in a multi-site, cohort study. *Vaccine*. 2024;42(7):1512-1520. doi:10.1016/j.vaccine.2024.01.016

177.	Roccato M, Russo S. A new look on politicized reticence to vaccination: Populism and COVID-19 vaccine refusal. *Psychol Med*. *Cambridge University Press*. 2023;53(8):3769-3770. doi:10.1017/S0033291721004736

178.	Debora Rola K, Russell YI. Knowledge is an Important Aspect of COVID-19 Vaccine Hesitancy. *N Am J Psychol*. Published online 2023:865-878. doi:10.6084/m9.figshare.24411805

179.	Rosenfeld DL, Tomiyama AJ. Jab my arm, not my morality: Perceived moral reproach as a barrier to COVID-19 vaccine uptake. *Soc Sci Med*. 2022;294. doi:10.1016/j.socscimed.2022.114699

180.	Samore T, Fessler DMT, Sparks AM, Holbrook C. Of pathogens and party lines: Social conservatism positively associates with COVID-19 precautions among U.S. Democrats but not Republicans. *PLoS One*. 2021;16(6 June). doi:10.1371/journal.pone.0253326

181.	Sanchez C, Dunning D. The anti-scientists bias: The role of feelings about scientists in COVID-19 attitudes and behaviors. *J Appl Soc Psychol*. 2021;51(4):461-473. doi:10.1111/jasp.12748

182.	Santavicca T, Ngov C, Frounfelker R, Miconi D, Levinsson A, Rousseau C. COVID-19 vaccine hesitancy among young adults in Canada. *Canadian Journal of Public Health*. 2023;114(1):10-21. doi:10.17269/s41997-022-00693-x

183.	Santirocchi A, Spataro P, Alessi F, Rossi-Arnaud C, Cestari V. Trust in science and belief in misinformation mediate the effects of political orientation on vaccine hesitancy and intention to be vaccinated. *Acta Psychol (Amst)*. 2023;237. doi:10.1016/j.actpsy.2023.103945

184.	Scandurra C, Bochicchio V, Dolce P, Valerio P, Muzii B, Maldonato NM. Why people were less compliant with public health regulations during the second wave of the Covid-19 outbreak: The role of trust in governmental organizations, future anxiety, fatigue, and Covid-19 risk perception. Published online 2023. doi:10.1007/s12144-021-02059-x/Published

185.	Schwartz B, Brewer J, Budigan H, et al. Factors Affecting SARS-CoV-2 Vaccination Intent and Decision Making Among African American, Native American, and Hispanic Participants in a Qualitative Study. *Public Health Reports*. 2023;138(3):422-427. doi:10.1177/00333549231160871

186.	Seale H, Heywood AE, Leask J, et al. COVID-19 is rapidly changing: Examining public perceptions and behaviors in response to this evolving pandemic. *PLoS One*. 2020;15(6 June). doi:10.1371/journal.pone.0235112

187.	Seddig D, Maskileyson D, Davidov E, Ajzen I, Schmidt P. Correlates of COVID-19 vaccination intentions: Attitudes, institutional trust, fear, conspiracy beliefs, and vaccine skepticism. *Soc Sci Med*. 2022;302. doi:10.1016/j.socscimed.2022.114981

188.	Sehgal NKR, Rader B, Gertz A, Astley CM, Brownstein JS. Parental compliance and reasons for COVID-19 Vaccination among American children. *PLOS Digital Health*. 2023;2(4 April). doi:10.1371/journal.pdig.0000147

189.	Sekimitsu S, Simon J, Lindsley MM, et al. Exploring COVID-19 Vaccine Hesitancy Amongst Black Americans: Contributing Factors and Motivators. *American Journal of Health Promotion*. 2022;36(8):1304-1315. doi:10.1177/08901171221099270

190.	Selleri P, Carugati F. Mothers and vaccinations: From personal experiences to shared representations. A challenge for healthcare authorities. *Italian Journal of Sociology of Education*. 2020;12(3):113-130. doi:10.14658/pupj-ijse-2020-3-6

191.	Serrano DM, Crone T, Williams PS. Exploring the Role of Multiplist Epistemic Beliefs on COVID-19 Conspiracies and Prevention Among Undergraduates. *Sci Educ (Dordr)*. Published online 2023. doi:10.1007/s11191-023-00447-2

192.	Simas C, Larson HJ, Paterson P. “Saint Google, now we have information!”: a qualitative study on narratives of trust and attitudes towards maternal vaccination in Mexico City and Toluca. *BMC Public Health*. 2021;21(1). doi:10.1186/s12889-021-11184-y

193.	Simione L, Vagni M, Gnagnarella C, Bersani G, Pajardi D. Mistrust and Beliefs in Conspiracy Theories Differently Mediate the Effects of Psychological Factors on Propensity for COVID-19 Vaccine. *Front Psychol*. 2021;12. doi:10.3389/fpsyg.2021.683684

194.	Smith AC, Woerner J, Perera R, Haeny AM, Cox JM. An Investigation of Associations Between Race, Ethnicity, and Past Experiences of Discrimination with Medical Mistrust and COVID-19 Protective Strategies. *J Racial Ethn Health Disparities*. 2022;9:1430-1442. doi:10.1007/s40615-021-01080-x/Published

195.	Sowa P, Kiszkiel Ł, Laskowski PP, et al. Covid-19 vaccine hesitancy in poland—multifactorial impact trajectories. *Vaccines (Basel)*. 2021;9(8). doi:10.3390/vaccines9080876

196.	Spire A, Sireyjol A, Bajos N. From intentions to practices: what drove people to get the COVID-19 vaccine? Findings from the French longitudinal socioepidemiological cohort survey. *BMJ Open*. 2023;13(12). doi:10.1136/bmjopen-2023-073465

197.	Sprengholz P, Bruckmann R, Wiedermann M, Brockmann D, Betsch C. From delta to omicron: The role of individual factors and social context in self-reported compliance with pandemic regulations and recommendations. *Soc Sci Med*. 2023;317. doi:10.1016/j.socscimed.2022.115633

198.	Stasiuk K, Polak M, Dolinski D, Maciuszek J. The credibility of health information sources as predictors of attitudes toward vaccination—the results from a longitudinal study in poland. *Vaccines (Basel)*. 2021;9(8). doi:10.3390/vaccines9080933

199.	Stoler J, Klofstad CA, Enders AM, Uscinski JE. Sociopolitical and psychological correlates of COVID-19 vaccine hesitancy in the United States during summer 2021. *Soc Sci Med*. 2022;306. doi:10.1016/j.socscimed.2022.115112

200.	Suhay E, Soni A, Persico C, Marcotte DE. Americans’ Trust in Government and Health Behaviors During the COVID-19 Pandemic. *RSF*. 2022;8(8):221-244. doi:10.7758/RSF.2022.8.8.10

201.	Šuriņa S, Martinsone K, Perepjolkina V, et al. Factors Related to COVID-19 Preventive Behaviors: A Structural Equation Model. *Front Psychol*. 2021;12. doi:10.3389/fpsyg.2021.676521

202.	Szczepańska D, Marchlewska M, Karakula A, Molenda Z, Górska P, Rogoza M. Dedicated to Nation but Against Women? National Narcissism Predicts Support for Anti-Abortion Laws in Poland. *Sex Roles*. 2022;87(1-2):99-115. doi:10.1007/s11199-022-01303-3

203.	Takamatsu A, Honda H, Miwa T, et al. Factors associated with COVID-19 booster vaccine hesitancy: a nationwide, cross-sectional survey in Japan. *Public Health*. 2023;223:72-79. doi:10.1016/j.puhe.2023.07.022

204.	Taylor CAL, Sarathchandra D, Kessler M. COVID-19 Vaccination Intake and Intention Among Black and White Residents in Southeast Michigan. *J Immigr Minor Health*. 2023;25(2):267-273. doi:10.1007/s10903-022-01401-0

205.	Teng Y, Hanibuchi T, Machida M, Nakaya T. Psychological determinants of COVID-19 vaccine acceptance: A comparison between immigrants and the host population in Japan. *Vaccine*. 2023;41(8):1426-1430. doi:10.1016/j.vaccine.2023.01.037

206.	Thorpe A, Zhong L, Scherer LD, Drews FA, Shoemaker H, Fagerlin A. Demographic, structural, and psychological predictors of risk-increasing and mask wearing behaviors among US adults between December 2020–March 2021. *Patient Educ Couns*. 2023;114. doi:10.1016/j.pec.2023.107792

207.	Tranter BK. Your money or your life? Public support for health initiatives during the COVID-19 pandemic. *Australian Journal of Social Issues*. 2022;57(3):544-561. doi:10.1002/ajs4.220

208.	Tzeng R, Huang FY, Lee J. Compliance, procrastination and refusal: American COVID-19 vaccination trust and value orientation. *Vaccine*. 2023;41(34):4950-4957. doi:10.1016/j.vaccine.2023.06.060

209.	Uddin S, Imam T, Khushi M, Khan A, Ali M. How did socio-demographic status and personal attributes influence compliance to COVID-19 preventive behaviours during the early outbreak in Japan? Lessons for pandemic management. *Pers Individ Dif*. 2021;175. doi:10.1016/j.paid.2021.110692

210.	Uluşahin Y, Mavor K, Reicher S. A political psychology of the link between populist beliefs and compliance with COVID-19 containment measures. *Front Polit Sci*. 2024;6. doi:10.3389/fpos.2024.1279798

211.	Utych SM, Navarre R, Rhodes-Purdy M. White Identity, Anti-Elitism, and Opposition to COVID-19 Restrictions in the United States. *Representation*. *Routledge*. 2022;58(2):301-310. doi:10.1080/00344893.2022.2075030

212.	Valasek CJ, Streuli SA, Pines HA, et al. COVID-19 vaccination acceptability and experiences among people who inject drugs in San Diego County. *Prev Med Rep*. 2022;30. doi:10.1016/j.pmedr.2022.101989

213.	Van Oost P, Yzerbyt V, Schmitz M, et al. The relation between conspiracism, government trust, and COVID-19 vaccination intentions: The key role of motivation. *Soc Sci Med*. 2022;301. doi:10.1016/j.socscimed.2022.114926

214.	van Prooijen JW, Etienne TW, Kutiyski Y, Krouwel APM. Just a Flu? Self-perceived infection mediates the link between conspiracy beliefs and Covid-19 health beliefs and behaviors. *J Health Psychol*. 2022;27(6):1421-1431. doi:10.1177/13591053211051816

215.	Vauclair CM. *A Serial Mediation Model Predicting Covid-19 Vaccines Acceptance in Portugal: The Critical Role of Conspiracy Theories in the Wake of Perceived Quality of Government Communication and National Stereotypes*. Vol 16. 2022. http://ijoc.org.

216.	Viskupič F, Wiltse DL, Meyer BA. Trust in physicians and trust in government predict COVID-19 vaccine uptake. *Soc Sci Q*. 2022;103(3):509-520. doi:10.1111/ssqu.13147

217.	Viskupič F, Wiltse DL. Attitudes toward annual COVID-19 boosters are highly structured by partisan self-identification and trust in government: Evidence from a longitudinal survey. *Vaccine X*. 2023;14. doi:10.1016/j.jvacx.2023.100337

218.	Viskupič F, Wiltse DL. Trust in physicians predicts COVID-19 booster uptake among older adults: Evidence from a panel survey. *Aging Health Res*. 2023;3(1). doi:10.1016/j.ahr.2023.100127

219.	Volek J, Moravec V. Undermining support for COVID-19 public health policies: an analysis of the impact of subversive conspiracy narratives on Czech General Practitioners. *Eur J Public Health*. 2024;34(1):69-74. doi:10.1093/eurpub/ckad194

220.	von Wyl V, Höglinger M, Sieber C, et al. Drivers of acceptance of COVID-19 proximity tracing apps in Switzerland: Panel survey analysis. *JMIR Public Health Surveill*. *JMIR Publications Inc.* 2021;7(1). doi:10.2196/25701

221.	Wagner AL, Wileden L, Shanks TR, Goold SD, Morenoff JD, Gorin SNS. Mediators of Racial Differences in COVID-19 Vaccine Acceptance and Uptake: A Cohort Study in Detroit, MI. *Vaccines (Basel)*. 2022;10(1). doi:10.3390/vaccines10010036

222.	Wamba SF, Guthrie C, Queiroz MM, Twinomurinzi H. Digital Technologies and COVID-19 Vaccine Acceptance: Evidence From France and South Africa. *Journal of Global Information Management*. 2023;31(1). doi:10.4018/JGIM.333611

223.	Wang J, Kim S. The paradox of conspiracy theory: The positive impact of beliefs in conspiracy theories on preventive actions and vaccination intentions during the covid-19 pandemic. *Int J Environ Res Public Health*. 2021;18(22). doi:10.3390/ijerph182211825

224.	Watts AW, Hutchison SM, Bettinger JA, et al. COVID-19 Vaccine Intentions and Perceptions Among Public School Staff of the Greater Vancouver Metropolitan Area, British Columbia, Canada. *Front Public Health*. 2022;10. doi:10.3389/fpubh.2022.832444

225.	Weinstein ER, Balise R, Metheny N, et al. Factors associated with latino sexual minority men’s likelihood and motivation for obtaining a COVID-19 vaccine: a mixed-methods study. *J Behav Med*. 2023;46(1-2):116-128. doi:10.1007/s10865-022-00315-4

226.	Williams SN, Armitage CJ, Tampe T, Dienes KA. Public perceptions of non-adherence to pandemic protection measures by self and others: A study of COVID-19 in the United Kingdom. *PLoS One*. 2021;16(10 October). doi:10.1371/journal.pone.0258781

227.	Williams SN. *Public Attitudes to COVID-19 ’booster Vaccinations and Influenza Vaccines: A Qualitative Focus Group Study*. 2021.

228.	Williams SN, Dienes K. Public attitudes to COVID-19 vaccines: A qualitative study. Preprint posted online May 18, 2021. doi:10.1101/2021.05.17.21257092

229.	Wilson RJI, Vergélys C, Ward J, Peretti-Watel P, Verger P. Vaccine hesitancy among general practitioners in Southern France and their reluctant trust in the health authorities. *Int J Qual Stud Health Well-being*. 2020;15(1). doi:10.1080/17482631.2020.1757336

230.	Woelfert FS, Kunst JR. How Political and Social Trust Can Impact Social Distancing Practices During COVID-19 in Unexpected Ways. *Front Psychol*. 2020;11. doi:10.3389/fpsyg.2020.572966

231.	Wotring AJ, Hutchins M, Johnson MK, et al. COVID-19 Vaccine Uptake Among College Students at a Midwest University. *J Community Health*. 2022;47(2):292-297. doi:10.1007/s10900-021-01051-7

232.	Wright L, Steptoe A, Fancourt D. Predictors of self-reported adherence to COVID-19 guidelines. A longitudinal observational study of 51,600 UK adults. *The Lancet Regional Health - Europe*. 2021;4. doi:10.1016/j.lanepe.2021.100061

233.	Wright D, Rune KT. Underlying motivators for anti-vaccination attitudes amongst regional Sunshine Coast parents in Australia. *Health Promotion Journal of Australia*. 2023;34(2):579-586. doi:10.1002/hpja.612

234.	Yuan S, Rui J, Peng X. Trust in scientists on COVID-19 vaccine hesitancy and vaccine intention in China and the US. *International Journal of Disaster Risk Reduction*. 2023;86. doi:10.1016/j.ijdrr.2023.103539

235.	Zarbo C, Candini V, Ferrari C, et al. COVID-19 Vaccine Hesitancy in Italy: Predictors of Acceptance, Fence Sitting and Refusal of the COVID-19 Vaccination. *Front Public Health*. 2022;10. doi:10.3389/fpubh.2022.873098

236.	Zarzeczna N, Bertlich T, Većkalov B, Rutjens BT. Spirituality is associated with Covid-19 vaccination scepticism. *Vaccine*. 2023;41(1):226-235. doi:10.1016/j.vaccine.2022.11.050

237.	Zatti A, Riva N. Bayesian subjectivism and psychosocial attitude toward COVID-19 vaccines. *F1000Res*. 2022;11. doi:10.12688/f1000research.121906.1

238.	Zidkova R, Malinakova K, van Dijk JP, Tavel P. COVID-19 Vaccination Refusal—Which Factors are Related in the Czech Republic, One of the Most Affected Countries in the World? *Int J Public Health*. 2023;68. doi:10.3389/ijph.2023.1605375

239.	Zimmermann BM, Paul KT, Araújo ER, et al. The social and socio-political embeddedness of COVID-19 vaccination decision-making: A five-country qualitative interview study from Europe. *Vaccine*. 2023;41(12):2084-2092. doi:10.1016/j.vaccine.2023.02.012

240.	Zysset AE, Schlatter N, Von Wyl A, Huber M, Volken T, Dratva J. Students’ experience and adherence to containment measures during COVID-19 in Switzerland. *Health Promot Int*. 2021;36(6):1683-1693. doi:10.1093/heapro/daab019

241.	Paul E, Fancourt D, Razai M. Racial discrimination, low trust in the health system and COVID-19 vaccine uptake: a longitudinal observational study of 633 UK adults from ethnic minority groups. *J R Soc Med*. 2022;115(11):439-447. doi:10.1177/01410768221095241

242.	Rivers P, Porter C, LeClair LB, et al. Longitudinal parental perception of COVID-19 vaccines for children in a multi-site, cohort study. *Vaccine*. 2024;42(7):1512-1520. doi:10.1016/j.vaccine.2024.01.016

243.	Spire A, Sireyjol A, Bajos N. From intentions to practices: what drove people to get the COVID-19 vaccine? Findings from the French longitudinal socioepidemiological cohort survey. *BMJ Open*. 2023;13(12). doi:10.1136/bmjopen-2023-073465

244.	Wright L, Steptoe A, Fancourt D. Predictors of self-reported adherence to COVID-19 guidelines. A longitudinal observational study of 51,600 UK adults. *The Lancet Regional Health - Europe*. 2021;4. doi:10.1016/j.lanepe.2021.100061
